# Visualizing increased uptake of [^18^F]FDG and [^18^F]FTHA in kidneys from obese high-fat diet fed C57BL/6J mice using PET/CT *ex vivo*

**DOI:** 10.1371/journal.pone.0281705

**Published:** 2023-02-14

**Authors:** Rakel Nyrén, Henrik Scherman, Jan Axelsson, Chuchun L. Chang, Gunilla Olivecrona, Madelene Ericsson

**Affiliations:** 1 Department of Medical Biosciences/Physiological Chemistry, Umeå University, Umeå, Sweden; 2 Department of Medical Biosciences/Pathology, Umeå University, Umeå, Sweden; 3 Department of Radiation Sciences, Umeå University, Umeå, Sweden; 4 Institute of Human Nutrition, Vagelos College of Physicians and Surgeons, Columbia University Irving Medical Center, New York, New York, United States of America; 5 Umeå Centre for Molecular Medicine, Umeå University, Umeå, Sweden; Midwestern University, UNITED STATES

## Abstract

It is known that high-fat diet (HFD) and/or diabetes may influence substrate preferences and energy demands in the heart preceding diabetic cardiomyopathy. They may also induce structural glomerular changes causing diabetic nephropathy. PET/CT has been utilized to examine uptake of energy substrates, and to study metabolic changes or shifts before onset of metabolic disorders. However, conventional PET/CT scanning of organs with relatively low uptake, such as the kidney, in small animals *in vivo* may render technical difficulties. To address this issue, we developed a PET/CT *ex vivo* protocol with radiolabeled glucose and fatty acid analouges, [^18^F]FDG and [^18^F]FTHA,to study substrate uptake in mouse kidneys. We also aimed to detect a possible energy substrate shift before onset of diabetic nephropathy. The *ex vivo* protocol reduced interfering background as well as interindividual variances. We found increased uptake of [^18^F]FDG and [^18^F]FTHA in kidneys after HFD, compared to kidneys from young mice on standard chow. Levels of kidney triglycerides also increased on HFD. Lipoprotein lipase (LPL) activity, the enzyme responsible for release of fatty acids from circulating lipoproteins, is normally increased in postprandial mice kidneys. After long-term HFD, we found that LPL activity was suppressed, and could therefore not explain the increased levels of stored triglycerides. Suppressed LPL activity was associated with increased expression of angiopoietin-like protein4, an inhibitor of LPL. HFD did not alter the transcriptional control of some common glucose and fatty acid transporters that may mediate uptake of [^18^F]FDG and [^18^F]FTHA. Performing PET/CT *ex vivo* reduced interfering background and interindividual variances. Obesity and insulin resistance induced by HFD increased the uptake of [^18^F]FDG and [^18^F]FTHA and triglyceride accumulation in mouse kidneys. Increased levels of [^18^F]FDG and [^18^F]FTHA in obese insulin resistant mice could be used clinically as an indicator of poor metabolic control, and a complementary test for incipient diabetic nephropathy.

## Background

Glucose and fatty acids are the most common energy substrates utilized by cells in mammals. However, different organs have different preferences and abilities to produce ATP from the available energy substrates that normally are taken up from blood by different transporter-mediated pathways. For glucose, several isoforms of glucose transporters have been reported to have different affinities for the glucose molecule [1]. Fatty acids that are delivered from the blood bound to albumin or as esterified parts of triglycerides (TG) in the core of plasma lipoproteins. The uptake of fatty acids in cells is not as well understood as that of glucose, but the uptake is supported by fatty acids binding proteins and transporters like CD36 and FATPs [2, 3]. For cells to be able to utilize fat in lipoproteins, the TG needs to be hydrolyzed to fatty acids and monoglycerides by lipoprotein lipase (LPL). This enzyme is produced by parenchymal cells in many tissues, and is transported to the luminal side of the capillary endothelium to act on plasma lipoproteins [4]. The activity of LPL is tightly regulated by several factors such as fasting/feeding, cold, exercise and obesity [5]. After fatty acids have been taken up by the cell they can either be used for immediate energy production via beta-oxidation or be stored as TG in lipid droplets for later use. In the murine heart, LPL activity is needed for TG accumulation in cardiomyocytes [6], which is needed for maintaining a balanced energy flow in the heart [7].

The activity of LPL in mouse kidneys is relatively high, and it responds to physiological regulators in a similar fashion to that in white adipose tissue. LPL activity is increased in the postprandial state, but reduced between meals and during fasting [8]. Down-regulation of LPL upon fasting is mainly post-translational and accomplished by interaction with the fasting-induced protein, angiopoietin-like protein 4 (ANGPTL4) [9, 10].

Kidneys are mitochondrial-dense and highly oxygen-consuming organs that need energy for important functions like nonstop tubular sodium reabsorption and urine production [11]. Molecules and ions are reabsorbed from the primary filtrate via ATP-dependent pumps in the tubular system. Depending on the metabolic conditions, the kidney can utilize different energy substrates such as fatty acids, glucose, lactate, and/or glutamine [12]. The substrate preference differs in different areas of thekidney parenchyma. The majority of the tubular cells in the cortex prefers fatty acids [12], whereas glucose is the preferred substrate for the tubular cells in the medulla [13]. This is due to limited oxygen supply and fewer mitochondria compared to cells in the cortex [14]. In diabetes, a greater demand for oxygen and ATP results in increased glucose metabolism in the kidney cortex, in addition to fatty acids utilization [11]. Mice given a high fat diet (HFD) for several weeks develop obesity and signs of metabolic syndrome (insulin resistance, hyperglycemia and lipid accumulation), and over time they may develop structural glomerular changes similarto diabetic nephropathy as well as diabetic cardiomyopathy [15–17]. The heart prefers fatty acids as its main energy source, although the heart utilizes a significant amount of glucose as well [18]. Energy metabolism and substrate preference is known to be disturbed in heart disease, such as heart failure [19, 20]. In heart failure, induced by long standing hypertension, a shift towards a fetal gene program and glucose metabolism is seen [21]. In the literature there are data indicating that metabolic changes occur before heart failure and morphological changes are manifested [19, 22]. Diabetic kidney disease is one of the leading causes of chronic kidney failure and end stage renal disease worldwide [23]. Also, renal contribution to gluconeogenesis is critical for systemic glucose homeostasis, mainly during fasting and conditions of stress [24].

The global obesity and diabetic epidemic is one of the leading causes behind metabolic and cardiovascular diseases [25]. If a high calorie diet is withheld for a prolonged time with a positive energy balance, obesity develops. The surplus energy is stored as TG, predominantly in adipose tissue depots, but over time non-adipose tissues may also accumulate TG [26]. If non-adipose tissues (organs) are overloaded by TG, they may suffer damage due to lipotoxicity leading to failure of important functions [26]. This risk applies to many organs including kidneys and hearts.

To visualize the substrate utilization in kidneys, and to investigate possible shifts due to diet or age that could precede the onset of disease, we have developed a protocol to study the uptake of the glucose analogue [^18^F]FDG (2-deoxy-2-[^18^F]fluoro-D-glucose) and the fatty acid analogue [^18^F]FTHA (14(R,S)-[^18^F]fluoro-6-thia-heptanodecanoic acid) in mouse kidneys using small animal hybrid positron-emission tomography and computed-tomography (PET/CT). Our first aim was to address some of the limitations [27], and develop a PET/CT protocol to detect and compare changes in uptake of FDG and [^18^F]FTHA (FTHA) in mouse kidneys. For comparison we studied uptake of the tracers in hearts, since the heart has been studied to a greater extent in the past [28, 29]. Our *ex vivo* PET/CT protocol enabled us to further study uptake of FDG and FTHA in kidneys of mice under different metabolic challenges (feeding/fasting, HFD or old age) to detect possible shifts and changes in energy substrate preferences. We also examined the potential to find correlations among the changes in uptake of FDG and FTHA, to TG accumulation, changes in expression of glucose and fatty acids transporters, as well as to the levels of LPL activity.

## Material and methods

### Study design

Male C57BL/6J mice, originally from Charles River, were bred in house for three generations, and kept at standard housing conditions (12:12 h light cycle, dark period 01:00–13:00 h; 21±1°C; 45–50% humidity). Mice had free access to water and standard (chow) rodent diet (CRM (E), SDS, Scanbur, Sweden), or a high-fat diet (D12492, Research Diets, NJ, USA), containing 60 kcal% from fat. The high-fat diet was introduced when the mice were 3 months old and was given for 5 months. For fasting, food was withdrawn 4 hours before the start of the experiments. Experiments started in the morning (around 09:00, due to the delivery of the isotopes). This was within the dark cycle and thus within the active period of the mice. Methods of sacrifice and anesthesia are described under the section experimental procedure.

All experiments were performed according to the EU Directive 2010/63/EU and approved by the Animal Review Board at the Court of Appeal for Northern Norrland in Umeå, Sweden (A2/14).

### PET/CT

#### Radiopharmaceuticals

FDG was prepared using GE Healthcare FASTlab synthesis module with a citrate buffer formulation and delivered from Norrlands University Hospital, Umeå, Sweden.

The second tracer used in the study was 14(R,S)-[^18^F]fluoro-6-thia-heptadecanoic acid (FTHA). A method to produce FTHA on the ScanSys synthesis module from Peter Larsen (ScanSys, Denmark) was developed. The synthesis method of FTHA originated from DeGrado [30] but the FTHA produced using this new method was formulated to exclude almost all ethanol from the final formulation solution. [^18^F] was produced using a PETtracer cyclotron from GE Healthcare. [^18^F] was transferred and trapped on a pre-conditioned QMA-column and eluted with a Kryptofix solution into a reaction vial. Water and acetonitrile were evaporated using azeotropic distillation at 120°C and 500 ml/min flow of helium. Two more additions of 600 μl acetonitrile were made under the same conditions before the temperature in the reaction vial was adjusted to 80°C and the ventilation needle was plugged. For production of FTHA, the precursor (Benzyl-14-(R,S)-tosyloxy-6-thiaheptadecanoate, ABX) dissolved in 500μl DMSO, was added to the reaction vial along with 500 μl acetonitrile and heated for 900 s after which 700 μl of 0.2 M KOH was added to the vial. Reaction temperature was adjusted to 95°C and the mixture was heated for additional 300 s. Heating was then turned off and 120 μl 1.0 M HCl in pH 7.4 phosphate buffer and 900μl HPLC-buffer was added to the reaction vial. The resulting reaction mixture was purified on preparative HPLC (ACE 5 C4, 150x10mm, MeCN:H_2_O (0.1% H_3_PO_4_) 60:40, 6 ml/min) and the collected fraction was transferred to a bottle containing 30 ml sodium ascorbate (15 mM). After transfer the solution was passed through a SPE-column which was washed with 2x4.5 ml of sterile water. The SPE was manually eluted with 1.5 ml 99.5% ethanol into a vial and 50 μl was taken for analytical HPLC (YMC-Triart C18, 150x4.6 mm, MeCN:H_2_O(0.1% TFA) 85:15, 1 ml/min). Ethanol was evaporated to dryness at 95°C under 200 ml/min flow of helium and then 1ml Intralipid (200mg/ml) was added to re-dissolve the product which was transferred into a new vial. The radiochemical purity was more than 95% and the shelf-life was set to two hours.

#### Experimental procedure

Food was removed at 07:00–08:00 in the morning for the fasted groups. The *ad libitum* groups were the first to receive the tracer, starting at 09:00 with 20 minutes intervals. The fasted groups were fasted for 4 hours before tracer administration. This allowed efficient use of the available tracer and increased the number of mice that could be scanned during one day at a similar nutrional state according to the daily circadian rhythm. The mice were placed on a heating pad while FDG (6.3 + 2.0 MBq) or FTHA (7.0 ± 2.0 MBq) was administered via tail vein injection (a total of 50–70 μl, diluted with saline to obtain an appropriate radioactive dose) under light isoflurane anesthesia (1–2% in 0.8 l O_2_ / min), Attane vet, VM Pharma, Sweden). A tailor-made catheter (PE20; inner diameter 0.38 mm, outer diameter 1.09 mm), with a 27G needle was used to minimize the risk for paravenous injection. The tracer was administered when blood flow was established in the catheter. To ensure that the entire dose was given, the catheter was immediately flushed with saline. After injection, the needle was removed and the remaining radiation in the catheter and syringe was measured. After injection the mice were placed in their home cages with excess amount of bedding material to avoid hypothermia. *Ad libitum* fed mice had continued access to food and water after isotope injection, while the fasted mice had access to only water until scanning. The mice were scanned 3 hours after administration of the isotope. This relatively long time was chosen to allow for unmetabolized tracer to be excreted via the urine. Thirteen animals were scanned both *in vivo* and *ex vivo*. For the *in vivo* scanning, mice were again sedated as described, and carefully placed on the integrated bed. The bed provided heating to avoid a decrease in body temperature. Mice, under continuous isoflurane anesthesia, were placed in prone position and scanned with CT and a 10-minute static PET scan (nanoScan, Mediso medical imaging system, Hungary). For *ex vivo* scanning the same acquisition protocol was used. After the *in vivo* scan (and for mice scanned only *ex vivo*) the mice were euthanized under deep (4%) isoflurane anesthesia. The anestethic depth was controlled through pain stimulation,by pinching the mouse toes with a toothed forceps.Circulating blood was then exchanged via the heart with cold PBS solution. The perfusion continued until the liver and kidneys were pale. The heart and both kidneys were carefully dissected and placed in a 15 ml plastic tube for a 10-minutes static scan. PET images were reconstructed to 0.4 x 0.4 mm resolution using the Mediso Tera-tomo 3D iterative reconstruction with 4 iterations and 4 subsets, and employing spike filter, delayed-window random correction, decay-correction, scatter and CT-based attenuation correction. Kidney and heart volumes-of-interests (VOIs) were delineated using CT Hounsfield values (50–750) in imlook4d software (https://github.com/JanAxelsson/imlook4d), and transferred to the PET images in the same software. For each VOI, the highest pixel, and the average pixel values were measured as standardized uptake values (SUV, ratio between measured PET-image activity concentration and injected whole body activity-concentration).

Mice dedicated to only have an *ex vivo* scan 3 hours after the isotope injection, were placed under deep (4%) isoflurane anaesthesia and the whole animal was perfused with buffer (PBS), to reduce the remaining tracer in the blood compartment, and all the surrounding tissues were removed. The organs were placed in a regular falcon tube for a static scan.

After the scan, tissues were rapidly excised, weighed and snap-frozen in liquid nitrogen for further analysis of TG content and LPL activity. Tissues for RT-qPCR were stored in RNA*later*^*®*^ (Invitrogen, Fisher Scientific, Sweden). LPL activity, TG content and RT-qPCR were measured after a total of 7 hours of fasting for fasted mice.

### Relative gene expression by real-time qPCR

RNA was extracted from tissue preserved in RNA*later*^*®*^ using RNeasy Kits from Qiagen (74104,74704) and treated with RNase-Free DNase (79254, Qiagen). To check for RNA quality, random samples were chosen and analyzed with Agilent RNA 6000 Pico Kit on a 2100 Bioanalyzer from Agilent Technologies, Waldbronn, Germany, according to manufacturer’s instructions. All samples had a RIN value from 9.40–10.0. cDNA was prepared with RevertAid H minus Reverse Transcriptase and Random hexamer primer (Thermo Fischer Scientific) in a ThermoCycler from Biometra. TaqMan assays were from ThermoFischer (Table 1). Gene expression was quantified by real time PCR using Maxima^™^ Probe/ROX qPCR master mix (Thermo Scientific) and the 7900HT Fast Real-Time PCR System, Applied Biosystems, with the software SDS 2.4. Relative gene expression was calculated according to the 2^-ΔΔCT^ method with *Rn18S* as the housekeeping gene.

**Table 1:** Summary of the RT-qPCR gene expression assays.

Gene	Description (protein abbreviation)	TaqMan assay
*Rn18S*	18s rRNA	Mm03928990_g1
*Slc5a2*	Sodium glucose co-transporter 2 (SGLT2)	Mm00453831_m1
*Slc2a1*	Glucose transporter 1 (GLUT1)	Mm00441480_m1
*Slc2a2*	Glucose transporter 2 (GLUT2)	Mm00446229_m1
*Slc2a4*	Glucose transporter 4 (GLUT4)	Mm00436615_m1
*Slc27a2*	Fatty acid transport protein 2 (FATP2)	Mm00449517_m1
*Slc27a6*	Fatty acid transport protein 6 (FATP6)	Mm01258609_m1
*Cd36*	Cluster of differentiation 36 (CD36)	Mm00432403_m1
*Ldhb*	Lactate dehydrogenase (LDH)	Mm01267402_m1
m*Lpl*	Mouse lipoprotein lipase (mLPL)	Mm00434764_m1
*Angptl4*	Angiopoietin-like protein 4 (ANGPTL4)	Mm00480431_m1

TaqMan gene expression assays (FAM-MGB) for RT-qPCR included in the study. All primers were purchased from Applied Biosciences/ThermoFischer.

### Triglyceride measurements

Samples of heart and kidney were homogenized in PBS and lipids were extracted using chloroform/methanol, as previously described [16]. TG content was measured using the Triglyceride GPO-PAP method (Roche Diagnostics, IN, USA).

### Measurement of LPL activity

All tissues were homogenized in 9 volumes of buffer containing protease inhibitors and detergents (0.025 M ammonia, pH 8.2, 5 IU heparin/ml, 1% Triton X-100, 0.1% SDS and 1 protease inhibitor pill (Complete^™^ Mini Protease Inhibitor, Sigma-Aldrich) per 50 ml using a BULLET BLENDER^™^ 24 (Next Advance, NY, USA). The homogenates were centrifuged at 2 C and aliquoted on ice. For measurements of lipase activity, radiolabeled substrate emulsions were prepared by sonicating triolein [9,10,3-^3^H(N)] (Perkin Elmer, MA, USA) and Intralipid^®^ (Fresenius Kabi, Uppsala, Sweden, 200 mg/ml) with 2.2% glycerol solution to reach a final TG concentration of 5 mg/ml. LPL activity in the tissue homogenates was measured as previously described at 25°C, pH 8.5 [31], but with human serum (5% vol/vol) as source of apolipoprotein CII. LPL activity is expressed as mU/g tissue or as mU/mg total protein, where one mU corresponds to 1 nmol fatty acids released per min. The amount of protein in the homogenates was measured using Markwell’s modified Lowry method [32].

### Glucose, insulin and NEFA measurements

Mice were fasted for 4 hours. Blood glucose was measured using a handheld Accuchek Aviva glucometer (Roche Diagnostics, Bromma, Sweden). Tail vein blood was collected in EDTA-coated tubes. The blood was centrifuged for 10 minutes at 4000 rpm at +4°C and the plasma was frozen and kept at -80°C. Insulin was measured using an ultra-sensitive mouse insulin ELISA kit (Crystal Chem, Zaandam, The Netherlands). Insulin resistance was calculated using HOMA-IR, (fasting glucose (mmol/l) x fasting insulin (mU/l))/22.5. NEFA was measured using the NEFA-HR(2) kit (Wako Chemicals, Neuss, Germany). Mouse plasma was used and measured according to manufactures protocol, but with a reduction of sample and reagent volumes by 50%.

### Statistics

All statistics were calculated using IBM SPSS Statistics 24 or Graph Pad Prism 8.1.1. Data were analyzed with Students paired or unpaired t-test or One-way ANOVA with Tukey’s *post hoc* test. Data are presented as mean ± standard deviation (SD), p<0.05 was considered significant.

## Results and discussion

### PET/CT imaging of mice kidneys *ex vivo*

**Fig 1:**
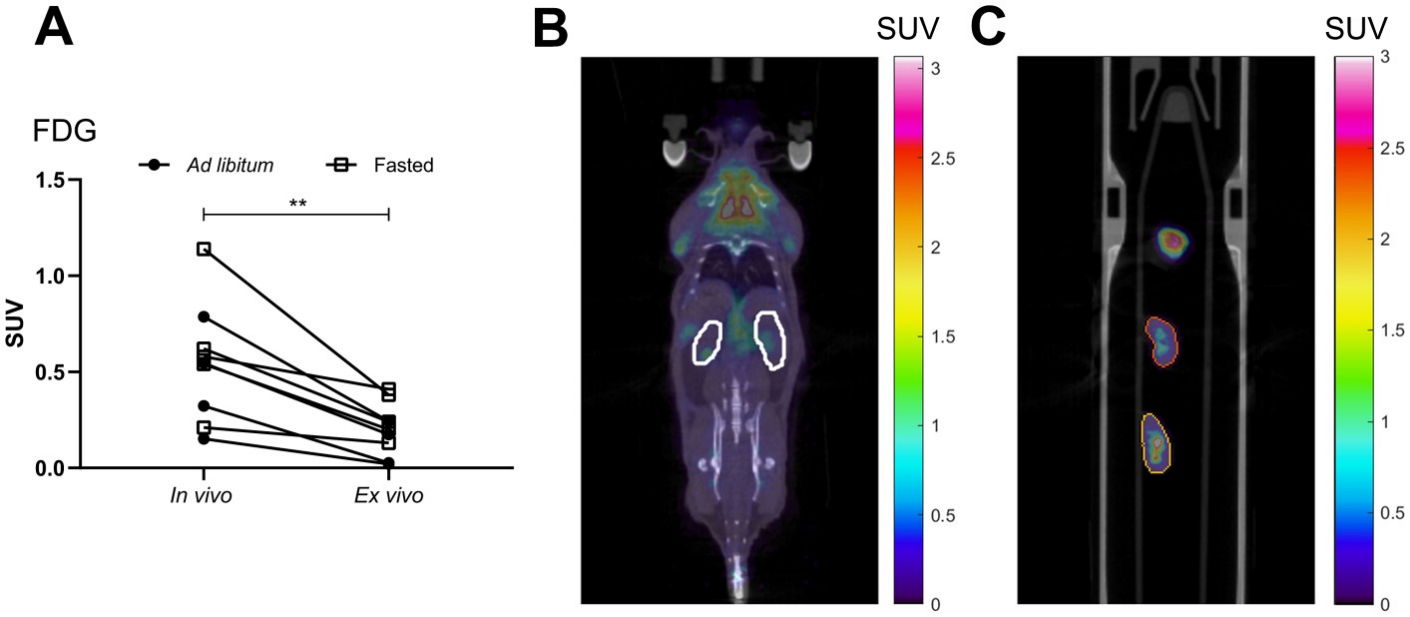
*In vivo* vs *ex vivo* PET/CT of mouse kidney. Old mice on chow diet, *ad libitum* fed or fasted (*n* = 4–5). (A) FDG SUV values for *in vivo* and *ex vivo* scans, (B) a representative *in vivo* scan of a fasted mouse and (C) *ex vivo* scan of organs from the same mouse. Data are presented as individual values. Students paired t-test. **p<0.01.

To reduce background radioactivity due to remaining tracer in urine, the radiolabeled substrate was injected 3 hours prior to scanning. After the injection, mice were able to move freely in their cages to maintain normal urine production. Despite our efforts to reduce remaining tracer in the urinary pool we noticed a 62% ± 56% higher (p<0.01) SUV-value *in vivo* compared to *ex vivo* (Fig 1). The thirteen animals investigated were scanned both *in vivo* and *ex vivo* (25 minutes apart). Before the *ex vivo* scan, the whole animal was perfused with buffer (PBS), to reduce the remaining tracer in the blood compartment, and all the surrounding tissues were removed. The organs were placed in a regular falcon tube for the static scan. The *ex vivo* scan also reduced the difficulties to determine the kidney border by CT. Therefore, we decided to perform scanning of the remaining mice only *ex vivo*.

### Uptake of FDG and FTHA in kidney

To study uptake of the tracers in an animal model with incipient metabolic dysfunction, we used C57BL/6J male mice on HFD. Five months of HFD induced obesity with elevated fasting glucose and insulin levels (Table 2).

**Table 2:** Weight and blood parameters from male C57BL/6J mice on chow or HFD.

	Chow young	Chow old	HFD	p-value
***n***	6	5	4	
**Age (months)**	3	10	9	
**Weight (g)**	26.4 ± 1.4	38.0 ± 2.9^a^	58.4 ± 4.0^a,b^	^a^p<0.0001, ^b^p<0.0001
**Glucose (mM)**	9.1 ± 0.4	9.7 ± 1.2	12.3 ± 1.1^a,b^	^a^p<0.001, ^b^p<0.01
**Insulin (ng/ml)**	0.6 ± 0.2	0.3 ± 0.1	2.3 ± 2.3^a,b^	^a^p<0.05, ^b^p<0.01
**HOMA-IR**	6.1 ± 1.5	3.8 ± 1.5	31.1 ± 22.1^a,b^	^a^p<0.05, ^b^p<0.01
**NEFA (mM)**	0.9 ± 0.1	1.1 ± 0.1	0.5 ± 0.1^a,b^	^a^p<0.01, ^b^p<0.001

Blood parameters were measured in mice fed either standard chow diet (young and old) or HFD. HFD was introduced at 3 months of age. All animals were fasted 4 hours before blood sampling. Significant difference compared to chow young (a) or chow old (b). Data are presented as mean ± SD. One-Way ANOVA, Tukey´s post hoc test.

Control mice of similar age on standard chow diet also gained weight but were not as obese as the mice on HFD, and were less insulin resistant. Data on bodyweight, age, organ weights and group sizes for mice scanned with PET/CT are found in Table 3.

**Table 3:** Age, body and organ weights from mice scanned with PET/CT.

	Chow young		Chow old		HFD	
	*Ad libitum*	Fasted	*Ad libitum*	Fasted	*Ad libitum*	Fasted
***n***	12	11	6	7	11	10
**Age (months)**	3	3	10±2	10±2	8	8
**Bodyweight (g)**	29.4±3.1	26.9±1.4	41.1±3.3	40.9±3.1	50.6±4.6	50.1±3.5
**Kidney (mg)**	164±16.2	150.1±17.4	220.8±38.8	229.1±13.1	198.3±37.6	186.9±28.7
**Heart (mg)**	136.0±17.7	121.6±10.9	166.3±16.8	167.4±18.7	149.2±20.8	146.8±21.6

Male C57BL/6J mice were either subjected to chow or high-fat diet (HFD). Chow fed animals were either young or of similar age as the HFD group. Mice on HFD started around 3 months of age and continued with HFD for the next 5 months. At the end of the experiment the mice were either fasted for 4 hours or remained ad libitum fed. Mice received either FDG or FTHA. All data are presented as mean ± SD.

**Fig 2:**
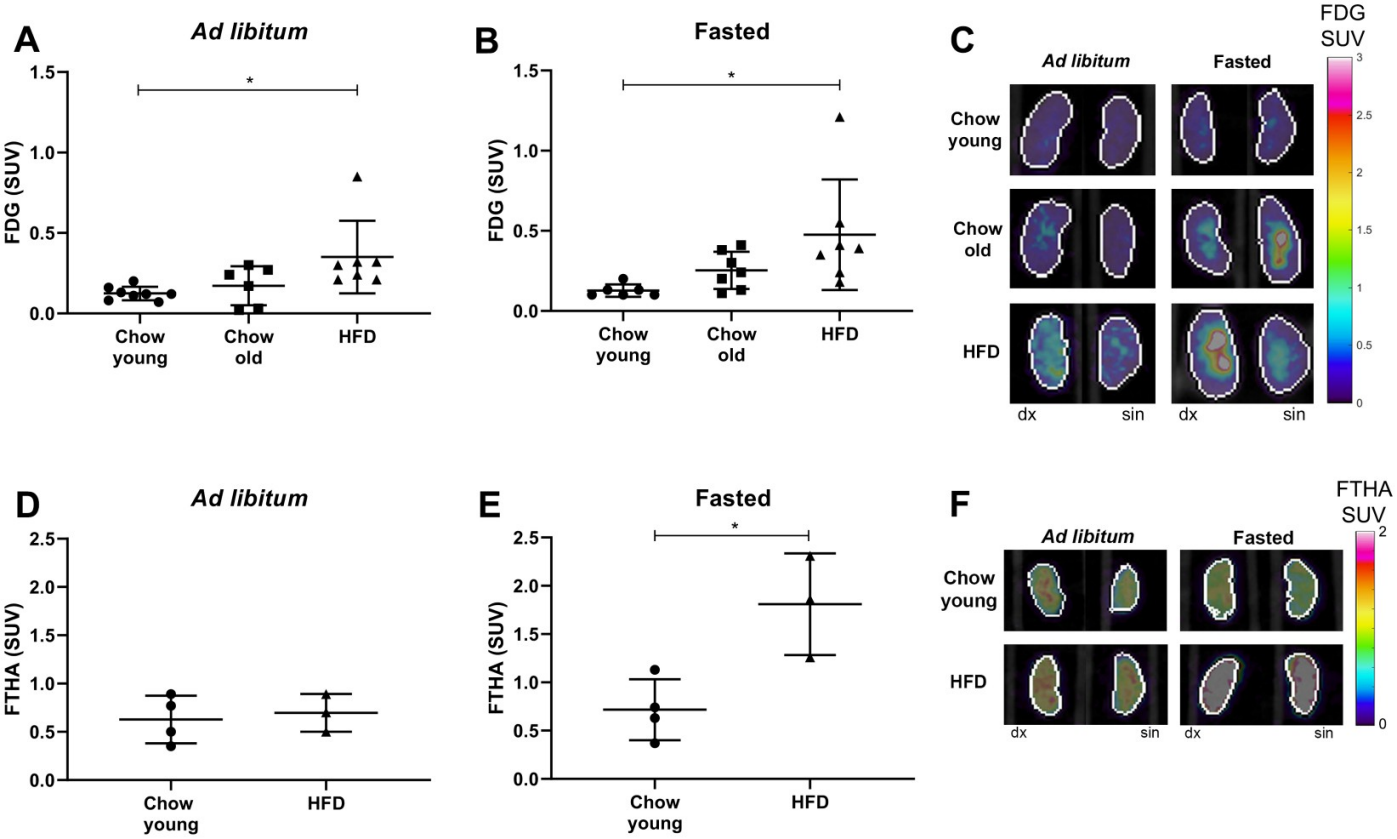
Effects on uptake of FDG or FTHA in mouse kidney by HFD or age. (A) Mean value of SUV in kidneys from *ad libitum* fed (*n* = 6–8) and (B) fasted mice (*n* = 6–7) injected with FDG. (C) Representative images of FDG uptake in kidney (both kidneys from the same individual). (D) Mean values of SUV in kidneys from *ad libitum* fed (*n* = 3–4) and (E) fasted (*n* = 3–4) mice injected with FTHA. (F) Representative images of FTHA uptake in kidney. Chow fed mice were either young (3 months old) or old (8 months), and high fat diet fed mice (HFD) were given HFD for 5 months starting from 3 months of age. Within each diet group, mice were either *ad libitum* fed or fasted (4 hours) before i.v. injection of FDG or FTHA. Data are presented as mean ± SD. One-Way ANOVA, Tukey´s post hoc test; *p<0.05. (dx = dexter; sin = sinister).

We aimed to investigate whether the metabolic changes induced by HFD would affect the uptake of FDG. To study if possible changes were due to diet or simply an effect of natural ageing, we included a group of young mice (3 months old) and used data from them as baseline. Since the nutritional state may influence substrate uptake, we studied both fasted and *ad libitum* fed mice. We found that FDG uptake in kidneys was at the same level in both fasted and *ad libitum* fed mice (Fig 2A and 2B). However, when the three groups with the same nutritional state were compared, we observed increased FDG uptake in kidneys from the obese HFD fed mice (Fig 2A and 2B). Old age was not enough to effect the uptake of FDG. In a previous pilot experiment we had investigated mice (*n* = 3–4) on a short-term HFD (for one month). There were no differences in the uptake of FDG in kidneys of these mice compared to young mice on chow in neither the fasted nor the *ad libitum* fed groups, demonstrating that longer exposure to HFD was needed to cause metabolic changes (kidney SUV mean ± SD for young chow fasted, 0.13 ± 0.04; young chow *ad libitum*, 0.12 ± 0.04; HFD (short-term) fasted, 0.12 ± 0.02; HFD (short-term) *ad libitum*, 0.14 ± 0.09). In several individuals we noticed differences in tracer uptake between the right and the left kidney. Therefore, the mean SUV-value for the right and the left kidney in PBS perfused animals, is presented for each individual.

Since natural ageing did not affect the uptake of FDG in kidneys, and uptake was increased only in mice on HFD for 5 months, we decided to examine the uptake of the fatty acid analogue FTHA only in mice fed HFD. Both fasted and *ad libitum* fed mice were scanned and compared to young mice on chow diet. For the *ad libitum* fed mice, the long-term HFD did not change the FTHA uptake (Fig 2D), but, for fasted mice, we observed an increased uptake of FTHA in the HFD group (Fig 2E). It appears that after a long-time on HFD the kidneys increase their FTHA uptake, but only in the fasted state. This is in contrast to uptake of FDG where HFD increased the uptake independently of nutritional state.

### Uptake of FDG and FTHA in heart

**Fig 3:**
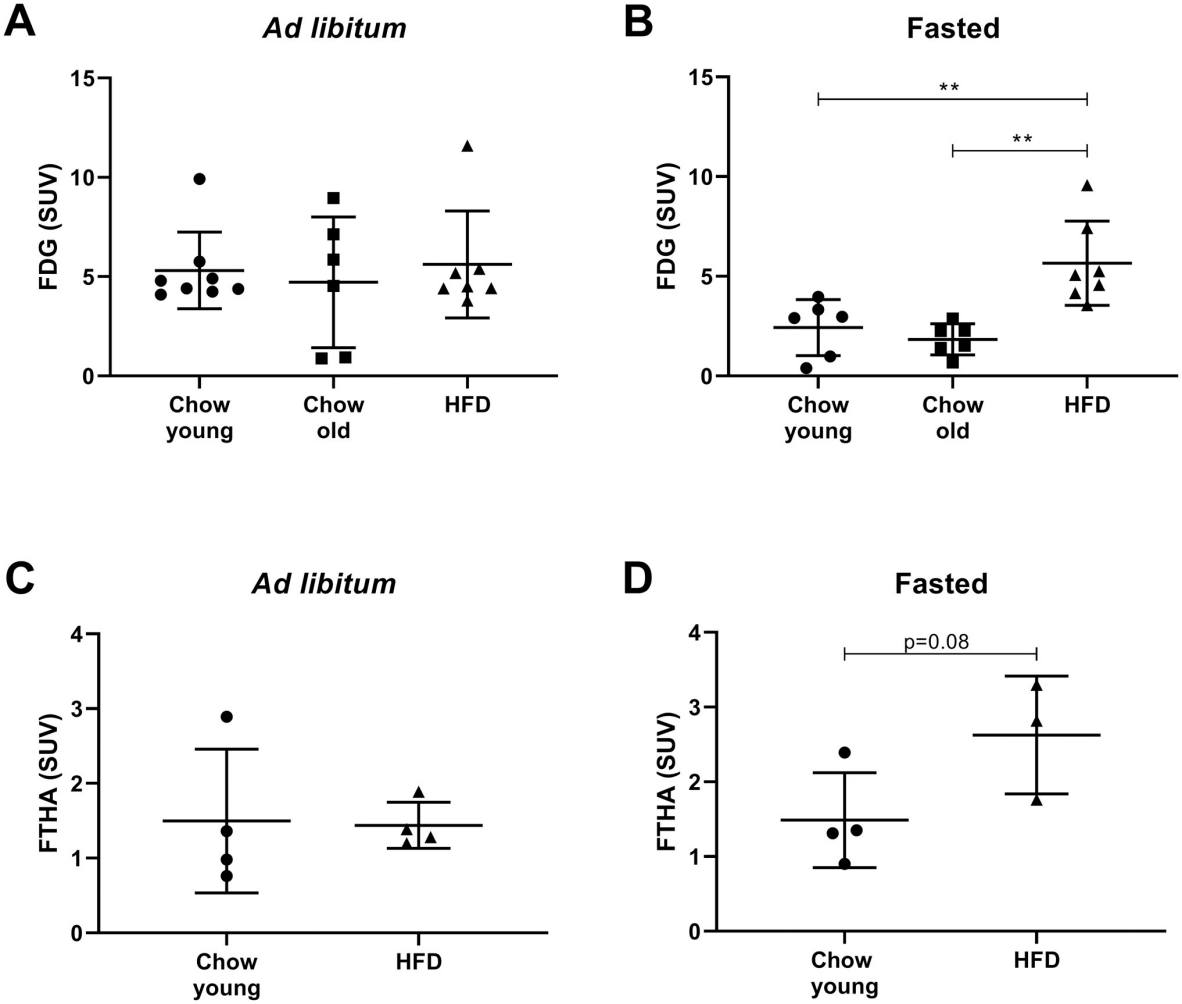
Effects on uptake of FDG and FTHA in mouse heart by HFD or age. Same mice as in Fig 2. (A) Mean value of SUV in heart from *ad libitum* fed (*n* = 6–9) and (B) fasted mice (*n* = 6–7) injected with FDG. (C) Mean values of SUV in heart from *ad libitum* fed (*n* = 4) and (D) fasted (*n* = 3–4) mice injected with FTHA. Chow fed mice were either young (3 months old) or old (8 months), and high fat diet fed mice (HFD) were given HFD for 5 months starting from 3 months of age. Within each diet group, mice were either *ad libitum* fed or fasted (4 hours) before i.v. injection of FDG or FTHA.Data are presented as mean ± SD. One-Way ANOVA, Tukey´s post hoc test or Students unpaired t-test; **p<0.01.

Since uptake of FDG in the mouse heart has been more thoroughly studied [28], we used hearts from the same animals for comparison. When comparing FDG uptake in hearts between *ad libitum* fed (Fig 3A), and fasted (Fig 3B) young mice, a significantly higher SUV-value was found in the *ad libitum* fed state (2.2 times higher, p<0.01). In our pilot experiment, described above, with short-term HFD no changes were noticed on FDG uptake in kidneys. However, in the hearts, only one month on HFD was enough to abolish the effect of nutritional state, meaning that the uptake was the same in both fasted and *ad libitum* fed mice (heart SUV mean ± SD for young chow fasted, 2.03 ± 1.41; young chow *ad libitum*, 5.48 ± 2.00, p = 0.004; HFD (short-term) fasted, 3.95 ± 1.86; HFD (short-term) *ad libitum*, 3.85 ± 2.30, p = 0.96). To compare the effect of long-term HFD or age, FDG uptake was compared within the same nutritional state. Neither old age nor HFD influenced FDG uptake in *ad libitum* fed mice (Fig 3A). However, in hearts from fasted mice, uptake of FDG was increased in the HFD group (Fig 3B).

The FTHA uptake in hearts was similar in both the young and the HFD group fed *ad libitum* (Fig 3C). In the fasted state, the FTHA SUV value for hearts was 1.8 times higher (p = 0.08) after HFD compared to the young mice on chow (Fig 3D).

For both kidney and heart, the effect of HFD on uptake of FDG or FTHA was mainly seen in the fasted state. Our data also show that obesity and reduced glucose handling induced by natural ageing did not influence the uptake of FDG and FTHA.

### Relative gene expression of glucose and fatty acid transporters

To investigate the mechanisms behind the observed increase in the uptake of FDG and FTHA after HFD, we measured the relative mRNA expression of the most abundant glucose and fatty acid transporters present in kidney and heart (Table 1). We included both *ad libitum* fed and fasted mice. In mice fed *ad libitum* the expression of *Slc2a2* (GLUT2) was increased in kidney after HFD (Table 4A).

**Table 4:** Relative gene expression of glucose and fatty acid transporters in kidney.

**A**	***Ad libitum***		
**Gene (protein)**	**Chow young**	**Chow old**	**HFD**
*Slc2a1* (GLUT1)	1.02 ± 0.22	0.99 ± 0.14	1.04 ± 0.20
*Slc2a2* (GLUT2)	1.01 ± 0.17	0.92 ± 0.15	1.33 ± 0.29^a^ ↑
*Slc5a2* (SGLT2)	1.01 ± 0.20	0.87 ± 0.13	0.90 ± 0.08
*Slc27a2* (FATP2)	1.02 ± 0.22	1.06 ± 0.14	1.20 ± 0.22
*Cd36* (CD36)	1.05 ± 0.33	1.09 ± 0.18	0.88 ± 0.19
*Ldhb* (LDH)	1.05 ± 0.39	0.86 ± 0.23	1.02 ± 0.25
**B**	**Fasted**		
**Gene (protein)**	**Chow young**	**Chow old**	**HFD**
*Slc2a1* (GLUT1)	1.02 ± 0.19	1.01 ± 0.26	1.18 ± 0.07
*Slc2a2* (GLUT2)	1.01 ± 0.12	0.97 ± 0.10	1.16 ± 0.36
*Slc5a2* (SGLT2)	1.01 ± 0.12	0.85 ± 0.14	0.79 ± 0.07^a^ ↓
*Slc27a2* (FATP2)	1.00 ± 0.09	1.05 ± 0.09	0.95 ± 0.14
*Cd36* (CD36)	1.01 ± 0.16	0.91 ± 0.13	0.75 ± 0.13^a^ ↓
*Ldhb* (LDH)	1.07 ± 0.45	1.11 ± 0.38	0.95 ± 0.26

Levels of mRNA were determined by RT-qPCR on tissue from young and old male mice fed chow or HFD. The mice were either ad libitum fed or fasted for 7 hours. Gene expression is relative to the levels in young mice on chow. Rn18s was used as housekeeping gene. Data are presented as mean ± SD. One-Way ANOVA with Tukey´s post hoc test. Significantly different from chow young (a). ↑/↓p<0.05.

HFD or old age did not change the expression levels of any of the other glucose or fatty acid transporters. In the fasted state there was no upregulation of neither glucose nor fatty acid transporters in old mice on chow or after HFD (Table 4B), despite increased FDG and FTHA uptake. In contrast, we noticed downregulation of the expression of *Cd36* and *Slc5a2* (SGLT2).

Pronounced effects were seen on the expression of glucose and fatty acid transporters in the hearts (Table 5).

**Table 5:** Relative gene expression of glucose and fatty acid transporters in heart.

**A**	***Ad libitum***		
**Gene (protein)**	**Chow young**	**Chow old**	**HFD**
*Slc2a1* (GLUT1)	1.01 ± 0.17	1.00 ± 0.09	0.76 ± 0.18^a,b^ ↓
*Slc2a4* (GLUT4)	1.00 ± 0.09	0.96 ± 0.06	0.75 ± 0.07^a,b^ ↓↓↓
*Slc27a6* (FATP6)	1.03 ± 0.26	0.69 ± 0.19	0.83 ± 0.22
*Cd36* (CD36)	1.00 ± 0.04	1.24 ± 0.09^a^ ↑↑	1.38 ± 0.16^a^ ↑↑↑
**B**	**Fasted**		
**Gene (protein)**	**Chow young**	**Chow old**	**HFD**
*Slc2a1* (GLUT1)	1.01 ± 0.18	0.77 ± 0.13^a^ ↓	0.66 ± 0.08^a,b^ ↓↓
*Slc2a4* (GLUT4)	1.01 ± 0.10	0.96 ± 0.16	0.71 ± 0.06^a,b^ ↓↓
*Slc27a6* (FATP6)	1.02 ± 0.23	0.88 ± 0.12	1.22 ± 0.39
*Cd36* (CD36)	1.00 ± 0.06	1.03 ± 0.07	1.08 ± 0.08

Levels of mRNA were determined by RT-qPCR on tissue from young and old male mice fed chow or HFD. The mice were either ad libitum fed or fasted for 7 hours. Gene expression is relative to the levels in young mice on chow. Rn18s was used as housekeeping gene. Data are presented as mean ± SD. One-Way ANOVA with Tukey´s post hoc test. Significantly different from chow young (a) or chow old (b). ↑/↓p<0.05, ↑↑/↓↓p<0.01, ↑↑↑/↓↓↓p<0.001.

The expression levels of *Slc2a1* (GLUT1) and *Slc2a4* (GLUT4) were decreased after HFD in both *ad libitum* fed and fasted mice (Table 5A and 5B) compared to the levels in young mice. The expression levels of the fatty acid transporter *Cd36* was increased in hearts of the group on HFD, but only in *ad libitum* fed mice (Table 5A).

### Triglyceride accumulation in kidney and heart

**Fig 4:**
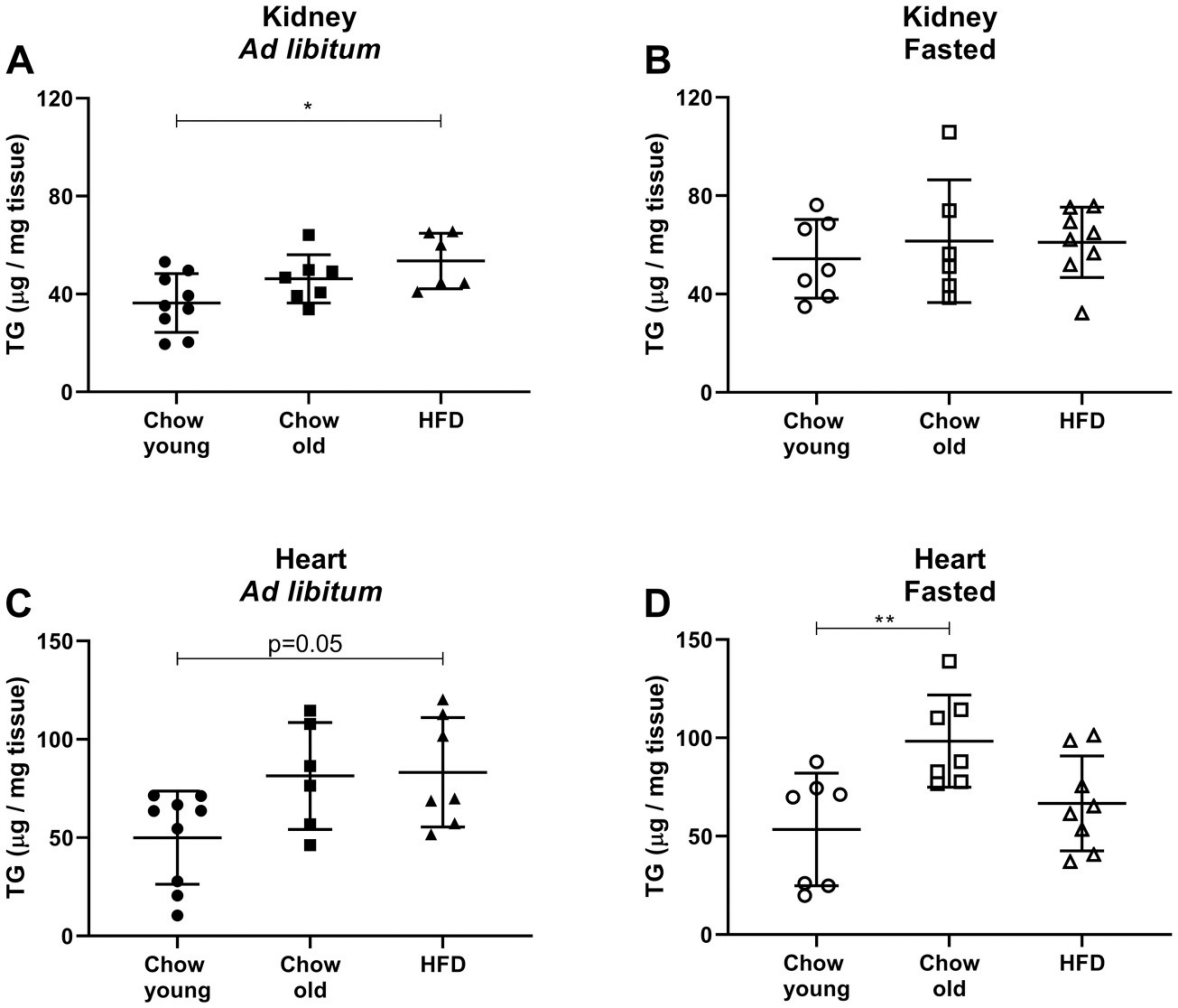
Triglyceride levels in mouse kidney and heart. Triglyceride (TG) (μg TG per mg wet tissue) was measured in kidneys from (A) *ad libitum* fed mice and (B) fasted mice (4+3 hours). TG in hearts from (C) *ad libitum* fed mice and (D) fasted mice (4+3 hours). Chow fed mice were either young (3 months old) or old (8 months), and high fat diet fed mice (HFD) were given HFD for 5 months from 3 months of age. Data are presented as mean ± SD. One-Way ANOVA with Tukey´s post hoc test; *p<0.05, **p<0.001.

The increased uptake of glucose and fatty acid analogues led us to investigate if the levels of TG in kidney and heart were affected by the HFD. The nutritional state has been previously shown to affect TG accumulation in the murine kidney. In the fasted state renal TG levels increase, compared to the fed state [33]. In our study we found the same pattern for the young mice only (data presented in separate panels, Fig 4A and 4B). Natural ageing and HFD abolished this nutritional effect on TG accumulation in kidney. In *ad libitum* fed mice, HFD inflicted an increase in total TG compared to young mice (Fig 4A), while no effect was seen for the fasted animals (Fig 4B).

In hearts, no nutritional effect was seen on TG levels in young mice. Elevated TG levels were only seen in hearts from fasted old mice (Fig 4C and 4D).

**Fig 5:**
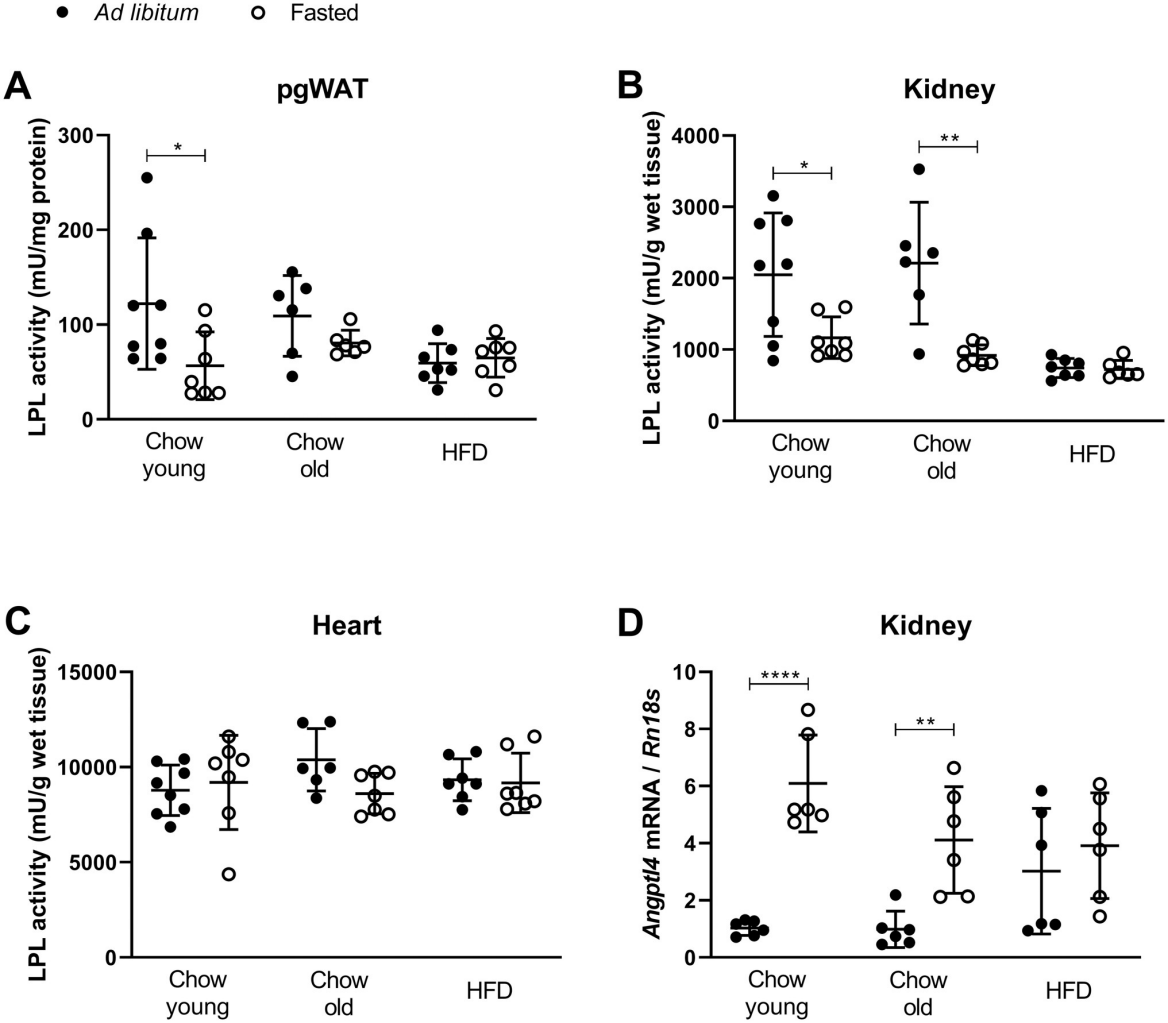
Effects of HFD and age on the nutritional regulation of LPL activity. LPL activity was measured in fasted (4+3 hours) and *ad libitum* fed mice (*n =* 6–8). (A) LPL activity in perigonadal white adipose tissue (pgWAT) is presented as mU/mg total protein. (B) LPL activity in kidney, expressed as mU/g wet tissue. (C) LPL activity in heart as mU/g wet tissue. (D) mRNA expression of *Angptl4* in kidneys, relative to *Rn18s*. Chow fed mice were either young (3 months old) or old (8 months), and high fat diet fed mice (HFD) were given HFD for 5 months from 3 months of age. Data are presented as mean ± SD. Students unpaired t-test; *p<0.05, **p<0.01, ****p<0.0001.

Previous studies had shown that LPL is important for accumulation of TG in the murine heart [6], and that murine kidneys contain high levels of LPL activity [8, 34]. We therefore measured LPL activity in kidneys and hearts in the present study. For comparison, LPL activity was measured in perigonadal white adipose tissue (pgWAT) to illustrate the well-known decrease of the activity of this enzyme on fasting [5, 35], and to record the effect of HFD and/or age on the regulation of the activity level. The expected downregulation of LPL activity by the 7-hour fasting was found in the young group on chow (Fig 5A), while this regulation was lost in the old group on chow, as well as in mice on HFD. A previous study from our lab found that LPL activity in murine kidneys is regulated by feeding-fasting in a similar fashion as that in white adipose tissue [8]. In our present groups of mice, down-regulation of LPL activity in kidneys was seen on fasting in young mice and in old mice on chow, as well as in mice on short-term HFD (experiment with HFD for one month, data not shown). After HFD for 5 months, the nutritional effect on the levels of LPL activity was lost (Fig 5B). As expected from many previous studies, LPL activity in homogenates of the hearts did not differ much in any of the groups (Fig 5C).

To investigate the possible reason for the blunted regulation of LPL activity in mouse kidneys after HFD, we measured the expression of *Angptl4*, a central factor for control of LPL activity. The mRNA levels for *Angptl4* were upregulated in the fasted state in the kidneys of all groups, except for the group on HFD (Fig 5D). On HFD the *Angptl4* expression was significantly higher (p<0.05) in the fed state than in the other groups (2.9 times higher compared to young *ad libitum-*fed mice).

In a previous study others had reported problems to define the mouse kidney using CT *in vivo* [27]. We also encountered difficulties delineating the true kidney volume. Our solution to this was to scan kidneys *ex vivo* after removal of surrounding tissue, like perirenal white adipose tissue. Other problems that were solved by our *ex vivo* protocol was that interference from tracer in blood was avoided, as well as from tracer in the urine of the tubular system, the renal pelvis, and the bladder. These sources may otherwise decrease the image quality and disturb the interpretation [36]. We optimized the experiments with the intent to avoid as much unbound residual tracer as possible. This was done by waiting for three hours for tracer-containing urine to be excreted, by perfusion of the blood compartment with PBS, and by dissecting out the intact kidneys and heart for scanning *ex vivo*.

*Ex vivo* scanning using FDG in the mouse has previously been described to distinguish different muscle types [37]. This method also allows to increase the number of animals that can be scanned during one batch of FDG delivery, although ruling out longitudinal studies using the same animals. By using our *ex vivo* protocol we documented changes in substrate uptake in mouse kidneys due to metabolic challenges using the PET/CT method for small animals. To induce metabolic changes (obesity and insulin resistance) we fed mice a HFD for 5 months. They were compared to mice of similar age on chow and to young chow fed mice (3 months of age). We chose male mice because they are known to be more sensitive to diet-induced metabolic changes compared to female mice [38].

Changes in the uptake of the glucose analogue FDG were measurable after HFD, with increased levels both in the fasted and the *ad libitum* fed mice. Increased uptake of the fatty acid analogue FTHA was found in kidneys of fasted mice that had been on HFD for 5 months, compared to the other groups. For comparison we investigated uptake of the tracers in the hearts of the same mice. FDG uptake was increased after HFD in the fasted state, but there was no significant increase in uptake of FTHA. We could, however, detect a trend and only few animals were included for the FTHA experiment. Our data goes in line with previous results in the literature that, in early stages of diabetic kidney disease, increased uptake of fatty acids and glucose occurs to satisfy the increased demand of ATP [11]. In the heart, similar changes are found in the pre-diabetic milieu [39, 40]. It appears that obesity and moderately reduced glucose handling induced by old age is not enough to inflict changes in FDG and FTHA uptake in mice, meaning that a heavier strain on the metabolic system (like long-term HFD) is needed for inflicting organ specific metabolic changes. The PET/CT-method is a non-invasive imaging technique, which is an advantage in the clinical situation. Increased uptake of e.g., the tracer FDG in an individual’s heart or kidneys could support that the organs are affected by manifested metabolic disease and that increased risk for cardiovascular and kidney disease may exist. By extending the knowledge on *in vivo* kidney glucose and fatty acid metabolism and its correletaion to development and/or manifestation of diabetic kidney disease, more precise/accurate treatment can be developed. It could also provide more information on how to interpret changes in background uptake due to metabolic disease when using PET/CT for other diagnostic purposes. One could also speculate that the level of FDG-uptake could be used as tool to evaluate the progress of diabetic kidney disease but also to see potential regress after a life-style intervention. Like glucose, FDG enters the cell via glucose transporters (GLUT) [1]. We analyzed the expression of the most abundant transporters in the kidney, GLUT1, 2 and SGLT2 [41]. In the proximal tubules, GLUT2 is present on the basolateral membrane while SGLT2 is on the luminal/apical side. SGLT2 reabsorbs glucose from the filtered primary urine and GLUT2 transports glucose back to blood [41]. PET analysis indicated the strongest signal for uptake of FDG corresponded to the central part of the kidney, the glycolytic medulla. SGLT2 has low affinity for FDG, which means that only little FDG is reabsorbed by this glucose transporter after filtration [42, 43]. This may contribute to the central accumulation of FDG found in our studies. In order to study if more FDG was taken up or accumulated in the glycolytic medulla due to metabolic changes/challenges, we attempted to quantify the cortical vs medullary FDG ratio. Despite scanning the kidneys *ex vivo*, we found it difficult to delineate the cortex-medullary border on the CT-images. With higher resolution from the CT-scan and possibly with an injected iodine contrast agent it might be possible to reach this goal in the future. Another approach could be to use a phosphor-imaging technique for a more detailed quantification, but that would mean injection of a very high dose of radioactive tracer.

A search in the literature unveiled that little is published on uptake of FTHA in mice, especially in mouse kidney. The majority of articles on uptake of FTHA and PET are from experiments performed on pigs or humans. Ci et al. analyzed the effect of insulin on uptake of FTHA in rats during a hyperinsulinemic clamp. They found a trend for increased levels of FTHA in rat kidneys [44]. This goes in line with our results in the obese, hyperinsulinemic mice after 5 months of HFD. Ci et al. extracted the FTHA from tissue homogenates, while we were able to visualize the increased uptake of FTHA by using PET. We found that the uptake of FTHA was evenly distributed over the whole kidney.

FTHA has been shown to be incorporated by esterification into complex lipids, but a relatively small fraction is found in TG [44, 45]. The FTHA was in our case administered intravenously to resemble free fatty acids. In preliminary experiments, when FTHA was given as an oral gavage in order to incorporate the tracer in chylomicron TG [46], we noticed that the majority of the tracer was trapped in the abdominal space and was only slowly transferred into the blood stream (data not shown). Furthermore, as earlier mentioned, LPL appears to play a minor role for providing kidneys with fatty acids derived from TG in lipoproteins. This means that the major uptake of fatty acids must originate from the pool of albumin-bound free fatty acids in blood. For these two reasons the fatty acid analogue FTHA was administered by intravenous injections in the tail vein in the present study.

Since we found an increased uptake of FDG and FTHA after long-term HFD we aimed to investigate mechanisms that could explain our findings. An increased uptake of FDG could be due to an increase in the amount of glucose transporters and/or their efficiency (greater influx of FDG into the cell). We found only small differences in the levels of mRNA that could correlate with the increased uptake of FDG in kidneys (increased expression of *Slc2a2* (GLUT2) in *ad libitum* fed mice on HFD). In the fasted HFD mice we found a decreased expression of *Slc5a2* (SGLT2). As mentioned earlier, SGLT2 has low affinity for FDG [42, 43], implying that changes in SGLT2 may not affect the uptake of FDG to the same extent as uptake of regular glucose. No changes in the expression of *Ldhb* was seen, indicating that there was no marked shift towards anaerobic metabolism in the kidneys of mice on HFD. For comparison we studied expression levels of some of the transporters in the hearts. Despite an increase in the uptake of FDG after HFD, both genes for GLUT1 and 4 were downregulated, while the fatty acid receptor *Cd36* was increased in the *ad libitum* HFD fed mice. Downregulation of *Slc2a4* (GLUT4) and increased *Cd36* expression has previously been found in hearts from mouse models for type 2 diabetes [47, 48]. Fatty acids are transported into the cardiomyocytes either through protein-mediated transport (via CD36 or FATP) or through a spontaneous “flip-flop” action [49]. The kidney expresses CD36 and FATP, but the renal uptake of fatty acids has been shown to be independent on these receptors/transporters [33]. Of note is, however, that differences in the levels of mRNA expression do not always correspond to differences in the levels of the translated protein. A limitation in our study is therefore that we did not measure protein levels or performed cell experiments to investigate the amount of transporters that were available on the cells surfaces.

Increased uptake of energy substrates can either be used to cover increased energy demands in the organ or be used for storage to cover future needs in the tissue. Glucose and fatty acids are stored as glycogen or as TG in lipid droplets, respectively. Accumulation of TG in tissues that are not specialized for lipid storage may have serious effects on several cell functions due to lipotoxicity [26]. We measured the amounts of TG in kidneys and hearts after HFD. In the kidneys the level was increased in the *ad libitum-*fed mice but was unchanged in fasted mice compared to mice on chow. Previous studies had shown that HFD causes increased storage of TG in heart [16, 50]. In our experiment, a tendency for increased levels of TG in the hearts were seen after HFD, but only in the *ad libitum-*fed mice. As mentioned earlier, accumulation of TG in the murine heart is dependent on LPL [6]. However, the total LPL activity measured in the tissue homogenates did not correlate with the increased TG levels. The explanation is most likely that the functional pool of active LPL that resides on the luminal side of capillaries is only a small fraction of the total tissue LPL activity, and cannot be easily quantified [51–53].

## Conclusions

Scanning of mouse kidneys *in vivo* may result in falsely high SUV-values for tracer uptake due to contributions of remaining unbound tracer and problems to define organ borders. This can be avoided by using the described *ex vivo* protocol and can be applied to any labeled substrate of interest. With contrast enhanced CT it should have been possible to define the kidney cortex and medulla and to study changes within these individual organs *in vivo*.

To our knowledge, this is the first PET/CT study to evaluate uptake of glucose and fatty acid analogues in mouse kidneys under different metabolic challenges. Significant differences in uptake of both tracers after HFD were observed in obese and insulin resistant mice, but not in old mice on chow diet. Potential changes in FDG and FTHA uptake seen in the pre-diabetic milieu could be an indicator that life-style changes are needed in patients to prevent heart or kidney disease from manifesting.

The responses to HFD for uptake of FDG and FTHA, as well as for stored TG, differed depending on the nutritional state of the animals, emphasizing the importance of clearly addressing the feeding status in this kind of experiments.

Disease caused by poor metabolic control, due to obesity and a sedentary lifestyle, is a great health threat and economic burden worldwide. To be able to study metabolic changes or shifts before the onset of disease, as here with PET/CT, could give possibilities to follow progression of both disease and treatment. Even more importantly, such information could be used to introduce lifestyle changes at early stages to avoid renal dysfunction.

## Supporting information

S1 Data Data corresponding to Figs 1–3. (PDF)

S2 Data Data corresponding to Fig 4. (PDF)

S3 Data Data corresponding to Fig 5. (PDF)

S4 Data Data corresponding to Table 2. (PDF)

S5 Data Data corresponding to Tables 4 and 5. (PDF)
