# Supplementary material for: Visualizing increased uptake of [18F]FDG and [18F]FTHA in kidneys from obese high-fat diet fed C57BL/6J mice using PET/CT ex vivo
Source: PLoS One. 2023 Feb 14;18(2):e0281705. doi: 10.1371/journal.pone.0281705 (PMC9928095; doi:10.1371/journal.pone.0281705)
Supplement: S1 Data — (PDF) [file pone.0281705.s001.pdf]

Kidney (sin + dx)/2

Applied ROI i CT-coronary view. Treshold within ROI Hounsfield (50-750). Import ROI to merged PET/CT view.

**Kidney**							
**Young**							
**Chow**			**n=7**				
**ad lib**							
**ID**	**SUV**	**SUVmax**	**ROI volume**	**Activity/ volume**	**BW**	**Inj dose (MBq)**	
**4470 (kid 25)**	0,13	0,65	0,11	1,21	25,90	4,10	
**4475 (kid 21)**	0,08	0,51	0,13	0,61	25,20	6,10	
**4476 (kid 22)**	0,16	0,60	0,10	1,58	26,60	5,20	
**4478 (kid 24)**	0,11	0,37	0,12	0,94	26,8	6,5	
**4910 (kid 50)**	0,12	0,58	0,15	0,77	31,9	5,6	
**4911 (kid 51)**	0,13	0,93	0,16	0,78	32,0	4,9	
**4913 (kid 52)**	0,20	1,58	0,17	1,17	32,6	8,7	
**4479 (kid30)**	0,07	0,43	0,20	0,33	26,6	5,8	
**Mean**	0,12	0,71	0,14	0,92	28,45	5,86	
**SD**	0,04	0,36	0,03	0,37	2,92	1,28	
	**0,33**	**0,52**	**0,23**	**0,40**	**0,10**	**0,22**	
**t-test ad lib vs fas**	**0,65**	**0,36**	**0,31**	**0,98**	**0,10**	**0,27**	
**4477 (kid 23)**	0,10	1,65	0,12	0,84	24,60	0,90	Injected dose correct? Exclud

**FTHA**	**SUV**	**SUVmax**	**ROI volume**	**Activity/ volume**	**BW**	**Inj dose (MBq)**
**5056 (kid 67)**	0,50	1,67	0,14	3,53	32,8	2,5
**5057 (kid 68)**	0,35	1,14	0,16	2,19	30,9	9,3
**5199 (Kid-72)**					30,5	8,3
**5200 (Kid-73)**						
**5203 (Kid-74)**	0,89	1,95	0,12	7,51	28,5	7,2
**5180 (Kid-75)**	0,77	1,90	0,12	6,30	33,1	6,7
**Mean**	0,63	1,67	0,14	4,88	31,16	6,80
**SD**	0,21	0,32	0,02	2,12	1,68	2,33

**Heart**							
**Young**							
**Chow**			**n=7**				
**ad lib**							
**ID**	**SUV**	**SUVmax**	**ROI volume**	**Activity/ volume**	**BW**	**Inj dose (MBq)**	
**4470 (kid 25)**	4,38	10,06	0,12	38,04	25,90	4,10	
**4475 (kid 21)**	4,24	7,98	0,09	45,91	25,20	6,10	
**4476 (kid 22)**	4,09	7,86	0,10	40,19	26,60	5,20	
**4478 (kid 24)**	5,75	12,52	0,15	38,83	26,8	6,5	
**4910 (kid 50)**	10,54	19,56	0,13	80,37	31,90	5,60	
**4911 (kid 51)**	5,62	10,05	0,13	42,81	32,00	4,90	
**4913 (kid 52)**	4,80	10,08	0,17	28,94	32,6	8,7	
**4479 (kid30)**	4,40	10,71	0,14	30,70	26,60	5,80	
**Mean**	5,48	11,10	0,13	43,22	28,45	5,86	
**SD**	2,00	3,49	0,02	15,01	2,92	1,28	
t-test ad lib vs fast	0,004	0,002	0,170	0,017	0,103	0,271	
**4477 (kid 23)**					24,60	0,90	Injected dose correct? Exclud

**FTHA**	**SUV**	**SUVmax**	**ROI volume**	**Activity/ volume**	**BW**	**Inj dose (MBq)**
**5056 (kid 67)**	1,36	4,02	0,12	11,08	32,8	2,5
**5057 (kid 68)**	0,76	1,71	0,12	6,56	30,9	9,3
**5199 (Kid-72)**				#DIVISION/0!	30,5	
**5200 (Kid-73)**				#DIVISION/0!		
**5203 (Kid-74)**	2,89	5,58	0,10	29,15	28,50	7,20
**5180 (Kid-75)**	0,98	1,90	0,11	8,76	33,1	6,7
**Mean**	**1,50**	**3,30**	**0,11**	**#DIVISION/0!**	**31,16**	**6,43**
**SD**	**0,83**	**1,60**	**0,01**	**#DIVISION/0!**	**1,68**	**2,47**

Kidney						
Young						
Chow			n=7			
fasted						
ID	SUV	SUVmax	ROI volume (c	Activity/ volur	BW	Inj dose (MBq)
4409 (kid 28)	0,13	0,57	0,13	0,99	27	6,5
4468 (kid 29)	0,10	0,60	0,11	0,91	25,9	6,1
4487 (kid 26)	0,13	0,52	0,10	1,22	24,7	5,3
4488 (kid 27)	0,10	0,54	0,11	0,90	25,7	5,4
4930 (kid 53)	0,10	0,57	0,18	0,58	27,6	9,2
4932 (kid 54)	0,20	0,80	0,13	1,58	26,8	6,4
4931 (kid55)	0,04	0,32	0,13	0,33	26,3	7,9
Mean	**0,11**	**0,56**	**0,13**	**0,93**	**26,29**	**6,69**
SD	**0,04**	**0,13**	**0,02**	**0,38**	**0,89**	**1,30**
CV%	**0,38**	**0,23**	**0,19**	**0,41**	**0,03**	**0,19**

FTHA						
5051 (Kid 60)	0,37	0,87	0,13	2,92	26,9	6,8
5052 (Kid 61)	1,13	2,81	0,16	7,19	26,3	7,3
5202 (Kid-76)	0,74	1,42	0,12	6,34	28,4	11
5181 (Kid-77)	0,63	1,19	0,13	4,99	29,9	9,5
Mean	**0,72**	**1,57**	**0,13**	**5,36**	**27,88**	**8,65**
SD	**0,27**	**0,74**	**0,02**	**1,61**	**1,40**	**1,69**

Heart						
Young						
Chow			n=7			
fasted						
ID	SUV	SUVmax	ROI volume (c	Activity/ volur	BW	Inj dose (MBq)
4409 (kid 28)	0,98	1,83	0,10	9,55	27	6,5
4468 (kid 29)	3,33	6,74	0,11	31,42	25,9	6,1
4487 (kid 26)	2,39	5,45	0,14	16,86	24,7	5,3
4488 (kid 27)	0,39	1,22	0,13	3,02	25,7	5,4
4930 (kid 53)	3,01	6,68	0,12	25,10	27,6	9,2
4932 (kid 54)	3,97	6,80	0,08	50,39	26,8	6,4
4931 (kid55)	0,14	0,40	0,10	1,34	26,3	7,9
Mean	**2,03**	**4,16**	**0,11**	**19,67**	**26,29**	**6,69**
SD	**1,41**	**2,67**	**0,02**	**16,19**	**0,89**	**1,30**

FTHA	SUV	SUVmax	ROI volume (c	Activity/ volur	BW	Inj dose (MBq)
5051 (Kid60)	1,31	3,01	0,11	12,17	26,90	6,80
5052 (Kid 61)	2,39	5,74	0,11	21,90	26,3	7,3
5202 (Kid-76)	0,90	2,15	0,10	8,81	28,4	11
5181 (Kid-77)	1,35	2,56	0,11	12,59	29,9	
Mean	**1,49**	**3,37**	**0,11**	**13,87**	**27,88**	**8,37**
SD	**0,55**	**1,41**	**0,00**	**4,86**	**1,40**	**1,87**

Kidney						
Young						
HFD	4 wks		n=3			
fasted						
ID	SUV	SUVmax	ROI volume (c	Activity/ volu	BW	Inj dose (MBq)
4367 (kid 34)	0,12	0,85	0,13	0,93	39,5	7,0
4366 (kid 33)	0,14	0,58	0,11	1,19	33,8	4,0
4354 (kid41)	0,10	0,53	0,12	0,85	32,5	3,0
4353 (kid 40)	0,06	0,54	0,14	0,42	33,8	0,7 Injected dose correct? Exclud
	0,12	0,65	0,12	0,99	35,27	4,67
	0,01	0,14	0,01	0,15	3,04	1,70
	0,11	0,22	0,06	0,15	0,09	0,36
t-test vs ad lib	0,77	0,45	0,81	0,84	0,45	0,92

Heart						
Young						
HFD	4 wks		n=3			
fasted						
ID	SUV	SUVmax	ROI volume (c	Activity/ volu	BW	Inj dose (MBq)
4367 (kid 34)	6,30	12,99	0,14	46,37	39,5	7,0
4366 (kid 33)	3,80	9,11	0,15	25,08	33,8	4,0
4354 (kid41)	1,74	4,56	0,11	15,27	32,5	3,0
4353 (kid 40)						0,7 Injected dose correct? Exclud
Mean	3,95	8,89	0,13	28,91	35,27	4,67
SD	1,86	3,44	0,02	12,98	3,04	1,70
t-test vs ad lib	0,96	0,94	0,74	0,96	0,45	0,92

Kidney						
Young						
HFD	4 wks		n=4			
ad lib						
ID	SUV	SUVmax	ROI volume	Activity/ volume	BW	Inj dose (MBq)
4302 (kid 37)	0,04	0,48	0,11	0,36	35,5	3,8
4352 (kid 35)	0,25	1,36	0,13	1,89	39,5	7,1
4364 (kid 38)	0,14	0,68	0,11	1,24	34,7	5,5
4365 (kid 39)	0,11	0,86	0,14	0,80	38,8	2,9
	0,14	0,85	0,12	1,07	37,13	4,83
	0,08	0,33	0,01	0,57	2,06	1,61
	0,56	0,38	0,10	0,53	0,06	0,33

Heart						
Young						
HFD	4 wks		n=4			
ad lib						
ID	SUV	SUVmax	ROI volume	Activity/ volume	BW	Inj dose (MBq)
4302 (kid 37)	0,39	1,48	0,08	4,92	35,5	3,8
4352 (kid 35)	6,68	13,90	0,13	51,61	39,5	7,1
4364 (kid 38)	4,86	11,64	0,17	29,31	34,7	5,5
4365 (kid 39)	3,47	7,31	0,13	27,13	38,8	2,9
Mean	3,85	8,58	0,13	28,24	37,13	4,83
SD	2,30	4,74	0,03	16,53	2,06	1,61

Kidney						
**Old**						
**Chow**		**n=5**				
**ad lib**						
**ID**	**SUV**	**SUVmax**	**ROI volume**	**Activity/ volum**	**BW**	**Inj dose (MBq)**
**3931 (kid 4-1)**	0,27	0,70	0,26	1,01	39,1	6,1
**3933 (kid 5-1)**	0,03	0,30	0,25	0,11	42,1	6
**4459 (kid 13-1)**	0,24	1,36	0,25	0,98	45	9,8
**4460 (kid 18-1)**	0,02	0,28	0,23	0,09	38,4	2,8
**4466 (kid14-1)**	0,17	0,82	0,18	0,96	37,3	7,4
**B83 (Kid56)**	0,30	1,67	0,13	2,29	44,7	6,2
**4458 (kid12-1)**	Too much spillover in PBS to make a clear ROI					
Mean	0,17	0,85	0,22	0,91	41,10	6,38
SD	0,11	0,51	0,05	0,73	3,02	2,07
	**0,65**	**0,60**	**0,22**	**0,81**	**0,07**	**0,32**
**t-test vs fasted**	**0,232**	**0,134**	**0,704**	**0,539**	**0,932**	**0,224**

Heart						
**Old**						
**Chow**		**n=5**				
**ad lib**						
**ID**	**SUV**	**SUVmax**	**ROI volume**	**Activity/ volum**	**BW**	**Inj dose (MBq)**
**3931 (kid 4-1)**	4,53	11,82	0,21	21,60	39,1	6,1
**3933 (kid 5-1)**	0,93	2,76	0,25	3,75	42,1	6
**4459 (kid 13-1)**	8,96	26,82	0,31	28,67	45	9,8
**4460 (kid 18-1)**	0,88	3,30	0,20	4,51	38,4	2,8
**4466 (kid14-1)**	7,13	14,47	0,18	39,86	37,3	7,4
**B83 (Kid56)**	5,86	11,26	0,12	50,69	44,7	6,2
**4458 (kid12-1)**	Too much spillover in PBS to make a clear ROI					
**Mean**	4,71	11,74	0,21	24,85	41,10	6,38
**SD**	3,01	8,03	0,06	17,21	3,02	2,07
t-test vs fasted	0,036	0,087	0,583	0,034	0,932	0,224

Kidney							
Old							
Chow		n=5					
fasted							
ID	SUV	SUVmax	ROI volume	Activity/ volume	BW	Inj dose (MBq)	
3935 (kid 6-1)	0,41	1,44	0,26	1,57	39	7,5	
3938 (kid 7-1)	0,13	0,68	0,29	0,45	37,3	7,8	
4461 (kid 19-1)	0,38	3,19	0,22	1,74	37,8	6	
4462 (kid 15-1)	0,24	3,43	0,25	0,96	40,3	8,9	
4464 (kid 16-1)	0,20	1,14	0,18	1,12	43,6	9,9	
B71 (kid58)	0,30	1,44	0,19	1,54	43,2	11,1	
B79 (kid 59)	0,11	0,55	0,20	0,58	45,4	4,7	
4463 (kid 20-1)	0,25	1,70	0,22	1,18	39,5	1,6	Injected dose correct? Exclude
4465 (kid 17-1)	0,77	5,27	0,18	4,27	44	10,9	Hydronephros
mean	0,25	1,69	0,23	1,14	40,94	7,99	
SD	0,11	1,07	0,04	0,47	2,91	2,04	
	**0,42**	**0,63**	**0,16**	**0,41**	**0,07**	**0,26**	

Heart							
Old							
Chow		n=5					
fasted							
ID	SUV	SUVmax	ROI volume	Activity/ volume	BW	Inj dose (MBq)	
3935 (kid 6-1)	2,88	9,67	0,31	9,33	39	7,5	
3938 (kid 7-1)	0,68	3,53	0,37	1,83	37,3	7,8	
4461 (kid 19-1)	2,27	5,89	0,20	11,42	37,8	6	
4462 (kid 15-1)	1,41	3,65	0,19	7,42	40,3	8,9	
4464 (kid 16-1)	2,26	8,18	0,29	7,83	43,6	9,9	
B71 (kid58)	1,52	3,76	0,15	10,49	43,2	11,1	
B79 (kid 59)	0,44	1,33	0,14	3,14	45,4	4,7	
4463 (kid 20-1)	0,25	1,70	0,22	1,18	39,5	1,6	Injected dose correct? Exclude
4465 (kid 17-1)	2,55	5,62	0,14	18,42	44	10,9	hydronefros
**Mean**	1,64	5,14	0,23	7,35	40,94	7,99	
**SD**	0,82	2,71	0,08	3,35	2,91	2,04	

Kidney						
	Old					
	HFD		n=7			
	ad lib					
	SUV	SUVmax	ROI volume (c	Activity/ volum	BW	Inj dose (MBq)
3939 (kid 8-1)	0,24	1,38	0,25	0,96	43,7	3,6
4167 (kid 31)	0,85	2,37	0,14	5,94	49,7	4,4
4169 (kid 36)	0,21	1,40	0,14	1,55	43,6	2,3
4472 (kid 42)	0,21	1,05	0,16	1,34	49,2	7,2
4413 (kid44)	0,32	1,07	0,14	2,32	53	8,2
4414 (kid45)	0,30	1,59	0,16	1,85	51	5,9
4412 (kid43)	0,32	1,58	0,15	2,10	51,4	4
Mean	0,35	1,49	0,16	2,30	48,80	5,09
SD	0,21	0,41	0,04	1,55	3,45	1,94
	0,60	0,28	0,22	0,68	0,07	0,38
t-test fed vs fasted	0,442	0,171	0,252	0,699	0,841	0,043

FTHA	SUV	SUVmax	ROI volume (c	Activity/ volum	BW	Inj dose (MBq)
4778 (Kid 63)	0,89	2,31	0,17	5,15	51,6	7,7
4782 (Kid 64)	0,70	2,27	0,15	4,61	49,3	6,3
4813 (kid 65)	0,50	1,76	0,18	2,73	55	5,8
4814 (kid 66)	2,60	15,94	0,26	9,93	59,8	6,6
Mean	0,70	2,11	0,17	4,17	51,97	6,60
SD	0,16	0,25	0,01	1,04	2,34	0,80

Heart						
	Old					
	HFD					
	ad lib					
	SUV	SUVmax	ROI volume (c	Activity/ volum	BW	Inj dose (MBq)
3939 (kid 8-1)	4,51	18,74	0,27	16,73	43,7	3,6
4167 (kid31)	4,42	10,41	0,15	28,57	49,7	4,4
4169 (kid 36)	5,39	11,45	0,14	38,71	43,6	2,3
4472 (kid42)	4,41	9,84	0,13	33,92	49,2	7,2
4413 (kid44)	5,18	10,16	0,13	38,94	53,0	8,2
4414 (kid45)	11,60	21,60	0,15	76,94	51,0	5,9
4412 (kid43)	3,79	6,60	0,13	30,01	51,4	4
Mean	5,61	12,68	0,16	37,69	48,80	5,09
SD	2,49	4,99	0,05	17,48	3,45	1,94
t-test	0,8759741	0,9794218	0,95029609	0,678870433	0,862203	0,085980565

FTHA	SUV	SUVmax	ROI volume (c	Activity/ volum	BW	Inj dose (MBq)
4778 (Kid 63)	1,39	2,73	0,12	11,74	51,6	7,7
4782 (Kid 64)	1,28	3,63	0,10	12,72	49,3	6,3
4813 (kid 65)	1,20	2,58	0,14	8,58	55	5,8
4814 (kid 66)	1,89	4,54	0,19	10,14	59,8	6,6
Mean	1,44	3,37	0,14	10,80	53,9	6,6

	Kidney<br>Old<br>HFD<br>fasted		n=6			
	SUV	SUVmax	ROI volume (c	Activity/ volur	BW	Inj dose (MBq)
**3929 (kid 3-1)**	0,41	3,75	0,27	1,52	53	10,1
**3934 (kid 10-1)**	0,24	1,66	0,45	0,52	45,2	6,1
**4166 (kid 32)**	0,35	1,41	0,13	2,75	45,3	5,5
**4484 (kid 46)**	0,18	1,00	0,16	1,12	48,8	6,5
**4485 (kid 47)**	0,39	2,72	0,14	2,72	51,2	6,3
**4486 (kid 48)**	1,21	7,94	0,15	8,23	51,4	8,2
**3930 (kid9)**	0,55	1,50	0,23	2,41	49,3	9,4
**Mean**	0,48	2,85	0,22	2,75	49,17	7,44
**SD**	0,32	2,25	0,11	2,37	2,79	1,66
	**0,67**	**0,79**	**0,49**	**0,86**	**0,06**	**0,22**

**FTHA**	**SUV**	**SUVmax**	**ROI volume (c**	**Activity/ volur**	**BW**	**Inj dose (MBq)**
**4777 (kid 69)**	1,26	3,39	0,17	7,29	54,9	7,8
**4815 (kid 70)**	1,86	3,85	0,14	13,10	54,7	4,9
**4781 (kid 71)**	2,31	5,18	0,14	16,91	48	4,5
**Mean**	1,81	4,14	0,15	12,44	52,5	5,7
**SD**	0,43	0,76	0,02	3,96	3,2	1,5

	Heart<br>Old<br>HFD<br>fasted					
	SUV	SUVmax	ROI volume (c	Activity/ volur	BW	Inj dose (MBq)
**3929 (kid 3-1)**	4,15	7,72	0,17	24,41	53	10,1
**3934 (kid 10-1)**	3,56	12,69	0,28	12,83	45,2	6,1
**4166 (kid 32)**	5,07	12,83	0,13	39,35	45,3	5,5
**4484 (kid 46)**	7,42	16,82	0,17	44,36	48,8	6,5
**4485 (kid 47)**	5,25	9,51	0,10	54,63	51,2	6,3
**4486 (kid 48)**	9,57	16,13	0,12	80,72	51,4	8,2
**3930 (Kid 9)**	4,57	10,49	0,21	21,71	49,3	9,4
**Mean**	5,66	12,31	0,17	39,72	49,17	7,44
**SD**	1,96	3,11	0,06	21,40	2,79	1,66

**FTHA**	**SUV**	**SUVmax**	**ROI volume (c**	**Activity/ volur**	**BW**	**Inj dose (MBq)**
**4777 (kid 69)**	1,76	4,49	0,13	13,29	54,9	7,8
**4815 (kid 70)**	2,82	5,65	0,10	27,04	54,7	4,9
**4781 (kid 71)**	3,30	8,51	0,09	37,41	48	4,5
**Mean**	2,63	6,22	0,11	25,91	52,5	5,7

FDG

	ad lib	fast	ad lib	fast
n	*7*	*7*	*6*	*7*
SUV	0.13 ± 0.04	0.14 ± 0.05	0.17 ± 0.11	0.27 ± 0.10
SUVmax	0.75 ± 0.37	0.56 ± 0.13	0.85 ± 0.51	1.98 ± 1.12

	HFD			
	Young		Old	
	ad lib	fast	ad lib	fast
n	*4*	*3*	*7*	*6*
SUV	0.14 ± 0.08	0.12 ± 0.01	0.35 ± 0.21	0.46 ± 0.34
SUVmax	0.85 ± 0.33	0.65 ± 0.14	1.49 ± 0.41	3.08 ± 2.36

FTHA

Kidney

	Chow			
	Young		Old	
	ad lib	fast	ad lib	fast
n	*2*	*2*		
SUV	0.43 ± 0.07	0.75 ± 0.38		
SUVmax	1.40 ± 0.26	1.84 ± 0.97		

	HFD			
	Young		Old	
	ad lib	fast	ad lib	fast
n			*3*	*3*
SUV			0.70 ± 0.16	1.81 ± 0.43
SUVmax			2.11 ± 0.25	4.14 ± 0.76

FDG

	ad lib	fast	ad lib	fast
n	*7*	*7*	*5*	*5*
SUV	5.44 ± 1.88	2.10 ± 1.43	4.49 ± 3.25	1.95 ± 0.80
SUVmax	11.17 ± 3.72	4.16 ± 2.67	11.83 ± 8.79	6.02 ± 2.32

	HFD			
	Young		Old	
	ad lib	fast	ad lib	fast
n	*4*	*3*	*7*	*6*
SUV	3.96 ± 2.36	3.89 ± 1.87	5.61 ± 2.49	5.56 ± 2.36
SUVmax	8.58 ± 4.74	8.89 ± 3.44	12.68 ± 4.99	12.62 ± 3.26

FTHA

Heart

	Chow			
	Young		Old	
	ad lib	fast	ad lib	fast
n	*2*	*2*		
SUV	1.06 ± 0.30	1.85 ± 0.54		
SUVmax	2.87 ± 1.16	4.38 ± 1.37		

	HFD			
	Young		Old	
	ad lib	fast	ad lib	fast
n			*3*	*3*
SUV			1.44 ± 0.27	2.63 ± 0.64
SUVmax			3.37 ± 0.79	6.22 ± 1.69
