# Supplementary material for: Visualizing increased uptake of [18F]FDG and [18F]FTHA in kidneys from obese high-fat diet fed C57BL/6J mice using PET/CT ex vivo
Source: PLoS One. 2023 Feb 14;18(2):e0281705. doi: 10.1371/journal.pone.0281705 (PMC9928095; doi:10.1371/journal.pone.0281705)
Supplement: S2 Data — (PDF) [file pone.0281705.s002.pdf]

							Kidney											Heart							
Mouse nr	Age	Nut	Organ Weights mg				0				TG							TG							
	Diet	state	KID dx	Heart	KID dx	Heart (B)	A1	A2	mean	mean-blank	TG (curve)	tot TG (x10) mmol/L	TG mg/dl	TG mg/ml	mg TG/tissue mg	ug TG/tissue mg	A1	A2	mean	mean-blank	TG (curve)	tot TG (dil (1/5 or 1/10) mmol/L	TG mg/dl	TG mg/ml	mg TG/mg tissue
3930		Fasted	204	166	73	28,2	0,202	0,22	0,211	0,1635	0,581603	5,816032	515,126	5,15126	5,527302	55,27302	0,166	0,171	0,1685	0,1205	0,61114	3,055699	270,6432	2,706432	0,098679
3934		Fasted	206	154	95	22,3	0,26	0,27	0,265	0,2175	0,737313	7,373126	653,0377	6,530377	7,150763	71,50763	0,099	0,103	0,101	0,053	0,263022	1,315111	116,4794	1,164794	0,053398
4166		Fasted	168	128	53	49,7	0,121	0,121	0,121	0,0735	0,322088	3,220877	285,273	2,85273	3,003925	30,03925	0,259	0,261	0,26	0,212	1,083032	5,415162	479,6209	4,796209	0,101299
4484		Fasted	158	151	75	47	0,129	0,144	0,1365	0,087	0,409156	4,091559	362,3894	3,623894	3,895686	38,95686	0,113	0,119	0,116	0,0665	0,311397	3,113972	275,8045	2,758045	0,06144
4485		Fasted	146	107	78	27	0,166	0,173	0,1695	0,12	0,566524	5,665236	501,77	5,0177	5,40908	54,0908	0,073	0,079	0,076	0,0265	0,120649	1,206485	106,8584	1,068584	0,040646
4486		Fasted	174	134	96	42	0,173	0,186	0,1795	0,13	0,614211	6,142108	544,0065	5,440065	5,962311	59,62311	0,113	0,113	0,113	0,0635	0,297091	2,970911	263,1336	2,631336	0,065282
4471	HFD	Fasted	157	145	80	50	0,162	0,167	0,1645	0,115	0,54268	5,4268	480,6517	4,806517	5,191038	51,91038	0,091	0,094	0,0925	0,043	0,199332	1,993324	176,5487	1,765487	0,037075
3939		Adlib	223	130	69	18	0,094	0,095	0,0945	0,047	0,245675	2,456747	217,5941	2,175941	2,326081	23,26081	0,089	0,091	0,09	0,042	0,206292	1,03146	91,35637	0,913564	0,051667
4167		Adlib	197	130	84	47,4	0,131	0,134	0,1325	0,085	0,355248	3,55248	314,6431	3,146431	3,410732	34,10732	0,164	0,163	0,1635	0,1155	0,585353	2,926766	259,2237	2,592237	0,057281
4169		Adlib	185	142	86	53,3	0,166	0,162	0,164	0,1165	0,446078	4,460744	395,0917	3,950917	4,290696	42,90696	0,171	0,175	0,173	0,1255	0,581766	5,817656	515,2698	5,152698	0,101826
4472		Adlib	174	146	93	24	0,148	0,152	0,15	0,1005	0,473534	4,735336	419,4087	4,194087	4,584137	45,84137	0,09	0,089	0,0895	0,04	0,185026	1,850262	163,8777	1,638777	0,069921
4412		Adlib	188	145	98	35	0,142	0,157	0,1495	0,1	0,471149	4,711493	417,2969	4,172969	4,58192	45,8192	0,137	0,145	0,141	0,0915	0,430615	4,306152	381,3959	3,813959	0,112784
4413		Adlib	155	133	58	42	0,132	0,136	0,134	0,0845	0,397234	3,972341	351,8303	3,518303	3,722364	37,22364	0,161	0,17	0,1655	0,116	0,547449	5,474487	484,8753	4,848753	0,120295
4414		Adlib	180	170	92	84	0,129	0,129	0,129	0,0795	0,373391	3,733906	330,712	3,30712	3,611375	36,11375	0,173	0,181	0,177	0,1275	0,602289	6,02289	533,4474	5,334474	0,06884
3931		Adlib	297	180	78	20	0,19	0,198	0,194	0,1465	0,532584	5,325836	471,7093	4,717093	5,085026	50,85026	0,102	0,108	0,105	0,0575	0,253739	2,537385	224,7362	2,247362	0,114615
3933		Adlib	248	181	84	24,2	0,2	0,217	0,2085	0,161	0,574394	5,743945	508,7412	5,087412	5,514754	55,14754	0,157	0,166	0,1615	0,1135	0,575039	2,875193	254,6559	2,546559	0,107776
4466		Adlib	217	156	74	22	0,172	0,171	0,1715	0,124	0,467705	4,677047	414,2461	4,142461	4,449003	44,49003	0,134	0,129	0,1315	0,0835	0,42032	2,101599	186,1386	1,861386	0,08647
4460		Adlib	235	159	81	29,6	0,145	0,136	0,1405	0,093	0,378316	3,78316	335,0745	3,350745	3,622155	36,22155	0,119	0,124	0,1215	0,0735	0,368747	1,843734	163,2995	1,632995	0,056802
4458		Adlib	252	169	76	29,7	0,133	0,132	0,1325	0,085	0,355248	3,55248	314,6431	3,146431	3,38556	33,8556	0,14	0,153	0,1465	0,0985	0,497679	2,488396	220,3972	2,203972	0,076412
4459		Adlib	214	181	71	36,9	0,107	0,124	0,1155	0,068	0,306228	3,062284	271,2265	2,712265	2,904836	29,04836	0,118	0,126	0,122	0,074	0,371325	1,856627	164,4415	1,644415	0,046208
3938	Chow old	Fasted	249	180	86	17,9	0,328	0,347	0,3375	0,29	0,946367	9,463668	838,1971	8,381971	9,10282	91,0282	0,107	0,111	0,109	0,061	0,304281	1,521403	134,7506	1,347506	0,076627
4461		Fasted	255	163	92	33,2	0,135	0,138	0,1365	0,089	0,366782	3,66782	324,8588	3,248588	3,547458	35,47458	0,164	0,169	0,1665	0,1185	0,600825	3,004126	266,0754	2,660754	0,082804
4462		Fasted	249	162	95	32,3	0,175	0,191	0,183	0,1355	0,500865	5,008651	443,6162	4,436162	4,857597	48,57597	0,173	0,168	0,1705	0,1225	0,621454	3,107272	275,2111	2,752111	0,087957
4463		Fasted	258	177	92	29	0,144	0,161	0,1525	0,105	0,412918	4,129181	365,7216	3,657216	3,99368	39,9368	0,191	0,191	0,191	0,143	0,727179	3,635895	322,0312	3,220312	0,114266
4464		Fasted	210	131	63	17,9	0,136	0,144	0,14	0,0925	0,376874	3,768743	333,7975	3,337975	3,548268	35,48268	0,153	0,161	0,157	0,109	0,551831	2,759154	244,3783	2,443783	0,138968
4465		Fasted	200	147	65	47	0,18	0,192	0,186	0,1385	0,509516	5,095156	451,2779	4,512779	4,80611	48,0611	0,132	0,136	0,134	0,0865	0,393632	3,936324	348,6402	3,486402	0,077665
3935		Fasted	268	190	93	21,5	0,307	0,337	0,322	0,2745	0,901672	9,016724	798,6113	7,986113	8,728821	87,28821	0,152	0,151	0,1515	0,1035	0,523466	2,617329	231,8168	2,318168	0,11014
4475		Adlib	138	105	65	38,8	0,126	0,13	0,128	0,0805	0,342272	3,422722	303,1505	3,031505	3,228553	32,28553	0,154	0,154	0,154	0,106	0,536359	2,681795	237,5266	2,375266	0,063593
4476		Adlib	155	129	95	23,8	0,126	0,132	0,129	0,0815	0,345156	3,451557	305,7044	3,057044	3,347463	33,47463	0,108	0,103	0,1055	0,0575	0,28623	1,43115	126,757	1,26757	0,054527
4477		Adlib	135	110	76	36,1	0,12	0,116	0,118	0,0705	0,313437	3,134371	277,6113	2,776113	2,987097	29,87097	0,095	0,09	0,0925	0,0445	0,219185	1,095926	97,06614	0,970661	0,027859
4478		Adlib	151	148	92	26,1	0,191	0,178	0,1845	0,0475	0,50519	5,051903	447,4471	4,474471	4,886122	48,86122	0,132	0,127	0,1295	0,0815	0,410005	2,050026	181,5708	1,815708	0,071383
4910		Adlib	204	146	97	39	0,144	0,15	0,147	0,0975	0,459227	4,592275	406,7378	4,067378	4,461913	44,61913	0,165	0,163	0,164	0,1145	0,540296	2,701478	239,2699	2,392699	0,063744
4911		Adlib	212	137	94	52	0,093	0,091	0,092	0,0425	0,196948	1,96948	174,4369	1,744369	1,908339	19,08339	0,075	0,075	0,075	0,0255	0,11588	0,579399	51,31738	0,513174	0,010382
4913	Chow	Adlib	179	143	89	47	0,089	0,088	0,0885	0,039	0,180258	1,802575	159,6541	1,596541	1,738633	17,38633	0,096	0,093	0,0945	0,045	0,20887	1,044349	92,498	0,92498	0,020605
4479	young	Adlib	163	112	55	32,1	0,072	0,069	0,0705	0,023	0,176471	1,764706	156,3	1,563	1,648965	16,48965	0,144	0,15	0,147	0,099	0,500258	2,501289	221,5392	2,215392	0,071231
4487		Fasted	131	114	67	22,4	0,094	0,096	0,095	0,137	0,247116	2,471165	218,8711	2,188711	2,335354	23,35354	0,113	0,121	0,117	0,069	0,345539	1,727695	153,0219	1,530219	0,069844
4488		Fasted	139	116	70	22,2	0,184	0,175	0,1795	0,132	0,490773	4,907728	434,6775	4,346775	4,651049	46,51049	0,139	0,128	0,1335	0,0855	0,430634	2,153172	190,7064	1,907064	0,087811
4468		Fasted	145	114	79	30	0,118	0,125	0,1215	0,074	0,323529	3,235294	286,55	2,8655	3,091875	30,91875	0,138	0,152	0,145	0,097	0,489943	2,449716	216,9714	2,169714	0,074494
4930		Fasted	146	112	92	47	0,143	0,14	0,1415	0,092	0,433	4,329995	383,5077	3,835077	4,187904	41,87904	0,108	0,104	0,106	0,0565	0,26371	1,31855	116,784	1,16784	0,026015
4932		Fasted	136	120	57	38	0,149	0,147	0,148	0,0985	0,463996	4,639962	410,9614	4,109614	4,343862	43,43862	0,086	0,084	0,085	0,0355	0,163567	0,817835	72,43565	0,724356	0,019786
4931		Fasted	135	110	81	31	0,17	0,175	0,1725	0,123	0,58083	5,808298	514,4409	5,144409	5,561106	55,61106	0,085	0,087	0,086	0,0365	0,168336	0,841679	74,54747	0,745475	0,024793
4409		Fasted	169	119	76	20,3	0,14	0,154	0,147	0,0995	0,397059	3,970588	351,675	3,51675	3,784023	37,84023	0,114	0,11	0,112	0,064	0,319752	1,598762	141,6024	1,416024	0,071171
