# Supplementary material for: Visualizing increased uptake of [18F]FDG and [18F]FTHA in kidneys from obese high-fat diet fed C57BL/6J mice using PET/CT ex vivo
Source: PLoS One. 2023 Feb 14;18(2):e0281705. doi: 10.1371/journal.pone.0281705 (PMC9928095; doi:10.1371/journal.pone.0281705)
Supplement: S3 Data — (PDF) [file pone.0281705.s003.pdf]

2018-12-04 Kidney 2 ul sample, incubation 2h

LPL activity

Group	Sample	DPM1	Average	SD	CV%	dpm/nmol / (-) blank	Activity mU/ml	mU/g
						**dpm/nmol**		
	20/198	84061,80	87916,07	3412,528	4	**257,2**		
		90553,00						
		89133,40						
	Bl	1087,50	1166,93	120,6771	10			
		1305,80					Activity	
		1107,50				(-) blank	mU/ml	mU/g
	St 22	7437,30	7128,20	496,41	7	5961,27	231,8	
		6555,60						
		7391,70						
	St 23	7297,00	6264,93	930,4543	15	5098,00	198,2	
		6007,50						
		5490,30						
	4470	9641,90	9280,30	817,2759	9	8113,37	315,4	3154,5
		8344,60						
		9854,40						
	4475	5075,50	6769,17	1471,097	22	5602,24	217,8	2178,2
		7728,90						
		7503,10						
	4476	4020,00	3874,50	148,2211	4	2707,57	105,3	1052,7
		3879,80						
		3723,70						
	4478	9447,10	8391,20	1454,62	17	7224,27	280,9	2808,8
		6732,00						
		8994,50						
Adlib	4910	4677,00	4741,93	325,8884	7	3575,00	139,0	1390,0
		4453,40						
		5095,40						

chow young		4911	7164,70	6822,27	399,2762	6	5655,34	219,9	2198,8
			6383,70						
			6918,40						
		4913	2971,30	3337,40	488,5898	15	2170,47	84,4	843,9
			3892,20						
			3148,70						
		4479	7350,80	8281,07	851,5355	10	7114,14	276,6	2766,0
			9022,00						
			8470,40						
	Fasted	4409	5380,30	5180,83	232,8249	4	4013,90	156,1	1560,6
			5237,20						
			4925,00						
		4468	4131,80	3996,17	172,6621	4	2829,24	110,0	1100,0
			3801,80						
			4054,90						
		4487	3279,00	3541,63	242,7586	7	2374,70	92,3	923,3
			3757,80						
			3588,10						
		4488	3653,20	3517,53	168,37	5	2350,60	91,4	913,9
			3329,10						
			3570,30						
		4930	5223,90	5271,50	70,3443	1	4104,57	159,6	1595,9
			5238,30						
			5352,30						
		4932	3628,20	3682,83	48,79962	1	2515,90	97,8	978,2
			3722,10						
			3698,20						
		4931	3885,50	3966,37	105,7972	3	2799,44	108,8	1088,4
			3927,50						
			4086,10						
		4367	3045,70	2874,83	154,3495	5	1707,90	66,4	664,0
			2745,50						
			2833,30						

HFD short	Fasted	4366	2743,60	2799,27	77,51499	3	1632,34	63,5	634,7
			2887,80						
			2766,40						
		4354	3787,10	3923,03	120,3887	3	2756,10	107,2	1071,6
			4016,20						
			3965,80						
	Adlib	4302	4221,50	4163,27	127,0849	3	2996,34	116,5	1165,0
			4017,50						
			4250,80						
		4352	5310,40	4813,20	430,6325	9	3646,27	141,8	1417,7
			4570,80						
			4558,40						
		4364	6851,40	6775,80	117,7749	2	5608,87	218,1	2180,7
			6835,90						
			6640,10						
		4365	5386,50	5517,20	267,2985	5	4350,27	169,1	1691,4
			5824,70						
			5340,40						
	Adlib	3931	7652,40	7479,60	982,8595	13	6312,67	245,4	2454,4
			6421,80						
			8364,60						
		3933	7486,70	6895,93	517,1963	8	5729,00	222,7	2227,5
			6524,80						
			6676,30						
		4459	6424,00	5713,13	615,9665	11	4546,20	176,8	1767,6
			5337,30						
			5378,10						
		4460	6721,90	7225,83	593,148	8	6058,90	235,6	2355,7
			7076,10						
			7879,50						
		4466	11301,40	10244,93	915,3194	9	9078,00	353,0	3529,6
			9689,90						
			9743,50						

chow old		B83		3359,80	3581,67	275,3297	8	2414,74	93,9	938,9
				3889,80						
				3495,40						
	Fasted		3935	4143,20	3961,37	179,5936	5	2794,44	108,6	1086,5
				3956,80						
				3784,10						
			3938	3278,10	3263,03	116,036	4	2096,10	81,5	815,0
				3370,80						
				3140,20						
			4461	4289,60	4080,93	333,5873	8	2914,00	113,3	1133,0
				4257,00						
				3696,20						
			4462	3447,50	3214,63	340,7789	11	2047,70	79,6	796,2
				2823,50						
				3372,90						
			4464	3279,70	3169,87	253,4739	8	2002,94	77,9	778,7
				2880,00						
				3349,90						
		B71		3563,20	3385,50	303,4656	9	2218,57	86,3	862,6
				3558,20						
				3035,10						
		B79		3548,50	3653,30	91,13819	2	2486,37	96,7	966,7
				3714,00						
				3697,40						
			3939	3587,80	3557,13	145,4453	4	2390,20	92,9	929,3
				3684,80						
				3398,80						
			4167	3100,10	3243,03	143,1003	4	2076,10	80,7	807,2
				3242,70						
				3386,30						
			4169	3247,40	3357,87	100,9087	3	2190,94	85,2	851,8
				3445,20						
				3381,00						

HFD old	adlib	4472	3174,10	3118,13	50,54823	2	1951,20	75,9	758,6
			3104,50						
			3075,80						
		4413	2528,50	2610,13	87,55412	3	1443,20	56,1	561,1
			2702,60						
			2599,30						
		4414	2654,00	2817,47	142,2569	5	1650,54	64,2	641,7
			2885,20						
			2913,20						
		4412	2857,10	2803,20	141,2839	5	1636,27	63,6	636,2
			2642,90						
			2909,60						
	Fasted	3934	3940,10	3622,43	285,9239	8	2455,50	95,5	954,7
			3385,70						
			3541,50						
		4166	3143,30	2907,67	213,348	7	1740,74	67,7	676,8
			2852,10						
			2727,60						
		4484	3179,30	3184,00	119,6193	4	2017,07	78,4	784,2
			3066,80						
			3305,90						
		4485	3022,10	2739,43	245,0391	9	1572,50	61,1	611,4
			2587,20						
			2609,00						
		4486	2830,60	2836,60	36,2741	1	1669,67	64,9	649,2
			2803,70						
			2875,50						
		3930	2829,90	2849,60	39,61225	1	1682,67	65,4	654,2
			2823,70						
			2895,20						

2018-12-04 pgWAT 2 ul sample, incubation 1h
LPL activity

			DPM1	Average	SD	CV%	dpm/nmol					
		20/198	94351,30	97981,93	3151,063	3	**286,6**					
			100004,80									
			99589,70									
		Bl	940,10	1077,00	163,0614	15						
			1257,40					Activity				
			1033,50				(-) blank	mU/ml	mU/g			
		St 22	5395,10	4928,53	587,3265	12	3851,53	268,8				
			4269,00									
			5121,50									
		St 23	3829,80	4335,73	617,556	14	3258,73	227,4			mg/ml	LPL activity
			5023,90							Protein	Corr dil	
			4153,50							ug/ml	1/50	mU/mg protein
		4470	3732,80	3456,87	295,249	9	2379,87	166,1	1660,8	46,532	2,33	71,38134
			3145,50									
			3492,30									
		4475	3245,70	4033,60	694,345	17	2956,60	206,3	2063,2	69,341	3,47	59,5095
			4299,00									
			4556,10									
		4476	6589,80	5664,23	877,2874	15	4587,23	320,1	3201,1	42,978	2,15	148,9664
			5558,00									
			4844,90									
		4478	10218,80	9777,47	434,7088	4	8700,47	607,2	6071,5	56,011	2,80	216,7969
			9763,90									
			9349,70									
	Adlib	4910	4974,10	4877,83	107,6479	2	3800,83	265,2	2652,4	26,093	1,30	203,3006
			4897,80									
			4761,60									

		4911	3955,20	4225,90	307,1502	7	3148,90	219,7	2197,4	31,425	1,57	139,8516
			4162,80									
			4559,70									
		4913	2495,20	3110,10	681,1674	22	2033,10	141,9	1418,8	38,534	1,93	73,6374
			3842,30									
			2992,80									
		4479	4566,70	4697,87	118,9077	3	3620,87	252,7	2526,8	77,043	3,85	65,59385
chow young			4798,60									
			4728,30									
		4409	3283,00	2970,00	275,1104	9	1893,00	132,1	1321,0	60,454	3,02	43,70281
			2860,50									
			2766,50									
		4468	3322,70	3278,77	166,9921	5	2201,77	153,6	1536,5	138,952	6,95	22,11517
			3094,20									
			3419,40									
		4487	4287,00	4211,10	398,3111	9	3134,10	218,7	2187,1	55,123	2,76	79,35308
			3780,30									
			4566,00									
		4488	5361,80	5033,83	440,6891	9	3956,83	276,1	2761,2	45,94	2,30	120,21
	Fasted		4532,90									
			5206,80									
		4930	2329,60	2432,60	116,9278	5	1355,60	94,6	946,0	68,749	3,44	27,52003
			2559,70									
			2408,50									
		4932	4116,90	3998,37	247,6747	6	2921,37	203,9	2038,6	36,165	1,81	112,7409
			3713,70									
			4164,50									
		4931	2605,60	2466,10	129,0771	5	1389,10	96,9	969,4	60,751	3,04	31,91272
			2441,80									
			2350,90									
		4367	2437,50	2574,37	147,4471	6	1497,37	104,5	1044,9	32,018	1,60	65,27062
			2730,50									
			2555,10									

HFD short	Fasted	4366	2185,90	2106,10	136,2307	6	1029,10	71,8	718,1	32,314	1,62	44,44784
			1948,80									
			2183,60									
		4354	2990,80	2892,80	231,7032	8	1815,80	126,7	1267,1	28,167	1,41	89,9728
			2628,20									
			3059,40									
	Adlib	4302	2544,80	2452,37	249,3487	10	1375,37	96,0	959,8	31,425	1,57	61,08394
			2642,30									
			2170,00									
		4352	2969,70	2834,73	219,7194	8	1757,73	122,7	1226,6	32,61	1,63	75,22912
			2581,20									
			2953,30									
		4364	2266,90	2243,67	128,4358	6	1166,67	81,4	814,1	29,055	1,45	56,0415
			2105,20									
			2358,90									
		4365	2173,00	2279,07	112,7598	5	1202,07	83,9	838,8	36,165	1,81	46,38995
			2397,50									
			2266,70									
	Adlib	3931	3838,90	3893,57	48,03065	1	2816,57	196,6	1965,5	28,463	1,42	138,1094
			3929,00									
			3912,80									
		3933	2987,30	2887,23	242,5124	8	1810,23	126,3	1263,2	36,165	1,81	69,86021
			2610,70									
			3063,70									
		4459	2691,80	2767,90	68,02448	2	1690,90	118,0	1180,0	20,405	1,02	115,6552
			2822,80									
			2789,10									
		4460	3303,30	3186,70	228,3412	7	2109,70	147,2	1472,2	64,798	3,24	45,44048
			2923,60									
			3333,20									
		4466	4507,20	4124,67	705,1631	17	3047,67	212,7	2126,8	27,341	1,37	155,5739
			3310,90									
			4555,90									

		B83	2514,20	2704,53	168,1438	6	1627,53	113,6	1135,8	17,399	0,87	130,5538
			2832,90									
chow old			2766,50									
		3935	3563,00	3283,80	251,9748	8	2206,80	154,0	1540,0	36,821	1,84	83,64716
			3073,30									
			3215,10									
		3938	3160,40	3189,80	28,91297	1	2112,80	147,4	1474,4	41,445	2,07	71,1492
			3190,80									
			3218,20									
		4461	3105,80	3252,83	331,021	10	2175,83	151,8	1518,4	38,902	1,95	78,06161
			3631,90									
	Fasted		3020,80									
		4462	2763,80	2526,03	223,7124	9	1449,03	101,1	1011,2	26,416	1,32	76,5588
			2319,70									
			2494,60									
		B71	2496,10	2573,73	68,05515	3	1496,73	104,4	1044,5	19,711	0,99	105,9789
			2602,00									
			2623,10									
		B79	2666,70	2566,53	113,9896	4	1489,53	103,9	1039,5	30,347	1,52	68,50437
			2590,40									
			2442,50									
		3939	3139,80	2876,07	228,7809	8	1799,07	125,5	1255,5	48,15	2,41	52,14765
			2757,40									
			2731,00									
		4167	3003,70	2827,00	158,6198	6	1750,00	122,1	1221,2	53,468	2,67	45,68019
			2696,90									
			2780,40									
		4169	2850,70	2831,43	216,145	8	1754,43	122,4	1224,3	78,902	3,95	31,03364
			3037,30									
			2606,30									
		4472	2905,00	2885,83	68,87992	2	1808,83	126,2	1262,3	34,277	1,71	73,65115
	adlib		2943,10									
			2809,40									

		4413	2433,20	2449,43	86,69742	4	1372,43	95,8	957,7	29,191	1,46	65,61847
			2372,00									
			2543,10									
		4414	3588,30	3565,43	92,54363	3	2488,43	173,7	1736,5	65,491	3,27	53,0308
			3644,40									
			3463,60									
		4412	3706,10	3436,83	374,7826	11	2359,83	164,7	1646,8	34,971	1,75	94,17965
			3008,80									
HFD old			3595,60									
		3929	3528,20	3246,27	244,3272	8	2169,27	151,4	1513,8	40,058	2,00	75,5801
			3096,30									
			3114,30									
		3934	3182,50	3163,57	94,53287	3	2086,57	145,6	1456,1	31,272	1,56	93,12374
			3247,20									
			3061,00									
		4166	3235,10	3517,63	281,2095	8	2440,63	170,3	1703,2	47,457	2,37	71,77713
			3797,50									
			3520,30									
		4484	3087,30	3108,40	257,4992	8	2031,40	141,8	1417,6	50	2,50	56,70342
	Fasted		2862,10									
			3375,80									
		4485	2677,20	2614,03	73,27403	3	1537,03	107,3	1072,6	42,139	2,11	50,90763
			2533,70									
			2631,20									
		4486	3050,20	3515,03	424,894	12	2438,03	170,1	1701,3	44,913	2,25	75,76199
			3611,50									
			3883,40									
		3930	2332,00	2236,90	83,88927	4	1159,90	80,9	809,4	52,543	2,63	30,80984
			2205,30									
			2173,40									

milky homogenate

Protein mg/ml new	LPL mU/mg new		Protein 2 runs	LPL mU/mg 2 runs protein
2,82845	58,7162		2,577525	64,43229
2,97145	69,43492		3,21925	64,09021
3,17575	100,7995		2,662325	120,2385
3,38	179,6303		3,090275	196,4713
0,7755	342,0195		1,040075	255,0163

2,07265	106,0197	1,82195	120,608
1,7458	81,26772	1,83625	77,26463
2,4812	101,8368	3,166675	79,79264
3,62515	36,44001	3,323925	39,74232
4,23795	36,25511	5,592775	27,47247
4,09495	53,40944	3,42555	63,84639
3,6047	76,60065	2,95085	93,57384
3,3596	28,15774	3,398525	27,83524
1,72535	118,1579	1,7668	115,3858
3,809	25,44933	3,423275	28,31689

Total protein pgWAT

	Result	MeanResul	New av	ug/ml Dil 50	protein mg/ml		Mean
4470	59,021	56,569		2828,45	2,828	2,3266	2,578
	60,246						
	50,441						
4475	55,344	59,429		2971,45	2,971	3,46705	3,219
	61,472						
	61,472						
4476	52,892	63,515		3175,75	3,176	2,1489	2,662
	70,051						
	67,6						
4478	72,503	67,6		3380	3,380	2,80055	3,090
	66,375						
	63,923						
4910	19,8	12,854	15,51	775,5	0,776	1,30465	1,040
	7,543						
	11,22						
4911	55,344	41,453		2072,65	2,073	1,57125	1,822
	33,282						
	35,733						
4913	40,636	34,916		1745,8	1,746	1,9267	1,836
	36,959						
	27,154						
4479	51,667	49,624		2481,2	2,481	3,85215	3,167
	45,538						
	51,667						
4409	65,149	72,503		3625,15	3,625	3,0227	3,324
	73,728						
	78,631						
4468	85,985	84,759		4237,95	4,238	6,9476	5,593

	82,308						
	85,985						
4487	78,631	81,899		4094,95	4,095	2,75615	3,426
	79,857						
	87,211						
4488	77,405	72,094		3604,7	3,605	2,297	2,951
	60,246						
	78,631						
4930	68,826	67,192		3359,6	3,360	3,43745	3,399
	61,472						
	71,277						
4932	28,379	34,507		1725,35	1,725	1,80825	1,767
	41,861						
	33,282						
4931	87,211	87,619	76,18	3809	3,809	3,03755	3,423
	110,498						
	65,149						

Heart 2 ul sample, incubation 1 h
LPL activity

			DPM1	Average	SD	CV%	dpm/nmol		
		20/198	79440,80	69405,13	8734,787	13	**203,0**		
			65259,40						
			63515,20						
		Bl	4695,60	1390,15	263,6801	19			
			1576,60					Activity	Activity
			1203,70				(-) blank	mU/ml	mU/g
		St 22	2952,20	3204,87	683,8529	21	1814,72	178,8	
			2683,30						
			3979,10						
		St 23	2389,50	2704,30	371,456	14	1314,15	129,5	
			3114,00						
			2609,40						
		4470	8898,40	8346,93	1181,616	14	6956,78	685,4	6853,974
			6990,40						
			9152,00						
		4475	8014,50	9045,57	893,2108	10	7655,42	754,2	7542,282
			9583,50						
			9538,70						
		4477	7813,80	9296,20	1285,73	14	7906,05	778,9	7789,212
			9966,90						
			10107,90						
		4478	11434,80	10041,60	1699,648	17	8651,45	852,4	8523,596
			8147,90						
			10542,10						
	Adlib	4910	10967,80	11963,37	883,273	7	10573,22	1041,7	10416,96
			12653,00						
			12269,30						
		4911	10977,10	10694,13	780,0429	7	9303,98	916,6	9166,486

chow young			9812,10						
			11293,20						
		4913	10942,70	11211,80	496,6258	4	9821,65	967,7	9676,502
			11784,90						
			10907,80						
		4479	11586,40	11850,50	991,2963	8	10460,35	1030,6	10305,76
			11018,00						
			12947,10						
		4409	9347,40	10997,10	1428,756	13	9606,95	946,5	9464,975
			11836,50						
			11807,40						
		4468	12687,50	12341,10	609,0983	5	10950,95	1078,9	10789,11
			11637,80						
			12698,00						
		4487	8844,60	9087,90	997,1654	11	7697,75	758,4	7583,99
			10184,20						
			8234,90						
		4488	6202,90	5824,67	368,0611	6	4434,52	436,9	4368,982
	Fasted		5467,70						
			5803,40						
		4930	11185,90	11925,83	647,967	5	10535,68	1038,0	10379,98
			12391,90						
			12199,70						
		4932	12824,60	13170,93	307,6562	2	11780,78	1160,7	11606,68
			13412,60						
			13275,60						
		4931	11856,30	11737,30	660,1933	6	10347,15	1019,4	10194,24
			12329,90						
			11025,70						
		4367	11115,70	11073,33	424,937	4	9683,18	954,0	9540,082
			10628,80						
			11475,50						
		4366	9531,50	9702,40	889,0045	9	8312,25	818,9	8189,409

	Fasted		9448,50						
			10127,20						
		4354	13685,00	12723,20	889,0045	7	11333,05	1116,6	11165,57
			11931,60						
			12553,00						
		4302	8472,90	8450,13	1092,228	13	7059,98	695,6	6955,649
HFD short			7346,70						
			9530,80						
		4352	6453,40	7569,07	1463,18	19	6178,92	608,8	6087,603
			9225,70						
	Adlib		7028,10						
		4364	10388,80	10611,00	296,8259	3	9220,85	908,5	9084,581
			10496,10						
			10948,10						
		4365	9833,10	10334,93	711,5851	7	8944,78	881,3	8812,594
			11149,30						
			10022,40						
		3931	10280,80	9879,77	384,4858	4	8489,62	836,4	8364,154
			9844,20						
			9514,30						
		3933	9539,90	11491,17	1751,022	15	10101,02	995,2	9951,741
			12925,60						
			12008,00						
		4459	14454,90	13955,47	772,7376	6	12565,32	1238,0	12379,62
			14346,10						
	Adlib		13065,40						
		4460	12534,80	11475,30	1106,073	10	10085,15	993,6	9936,108
			10327,90						
			11563,20						
		4466	12659,20	10864,27	1588,315	15	9474,12	933,4	9334,105
			9640,60						
			10293,00						
		B83	13413,70	13903,53	691,1486	5	12513,38	1232,8	12328,46

				14694,10						
				13602,80						
			3935	11376,80	11290,97	667,6012	6	9900,82	975,4	9754,499
chow old				11911,50						
				10584,60						
			3938	9497,50	9022,47	571,3275	6	7632,32	752,0	7519,524
				8388,50						
				9181,40						
			4461	9765,80	9280,30	505,85	5	7890,15	777,4	7773,547
				8756,30						
				9318,80						
			4462	12142,70	11107,97	1132,686	10	9717,82	957,4	9574,204
	Fasted			11283,40						
				9897,80						
			4464	8475,80	8904,63	391,9645	4	7514,48	740,3	7403,432
				9244,40						
				8993,70						
		B71		12099,20	11241,90	1309,963	12	9851,75	970,6	9706,158
				11892,50						
				9734,00						
		B79		11099,30	10016,50	1177,225	12	8626,35	849,9	8498,867
				8763,40						
				10186,80						
			3939	10972,00	10650,63	331,0407	3	9260,48	912,4	9123,629
				10669,20						
				10310,70						
			4167	10492,80	10645,63	273,1586	3	9255,48	911,9	9118,703
				10961,00						
				10483,10						
			4169	10943,70	10951,30	290,9744	3	9561,15	942,0	9419,852
				11246,00						
				10664,20						
			4472	13514,20	12355,77	1026,847	8	10965,62	1080,4	10803,56

	Adlib		11557,60						
			11995,50						
		4413	9381,70	9960,10	590,7657	6	8569,95	844,3	8443,3
			9936,10						
			10562,50						
		4414	13105,50	12208,07	777,99	6	10817,92	1065,8	10658,05
			11724,30						
			11794,40						
		4412	7985,70	9268,43	1341,925	14	7878,28	776,2	7761,856
			10662,60						
HFD old			9157,00						
		3929	12496,10	12752,83	264,4622	2	11362,68	1119,5	11194,76
			13024,40						
			12738,00						
		3934	9441,20	9724,30	425,9776	4	8334,15	821,1	8210,985
			9517,50						
			10214,20						
		4166	10384,40	10124,80	375,773	4	8734,65	860,6	8605,567
			10296,10						
			9693,90						
		4484	9915,80	9593,67	328,7908	3	8203,52	808,2	8082,282
	Fasted		9606,60						
			9258,60						
		4485	9265,80	9301,90	259,4406	3	7911,75	779,5	7794,828
			9062,40						
			9577,50						
		4486	13446,40	13176,47	442,6611	3	11786,32	1161,2	11612,13
			12665,60						
			13417,40						
		3930	10732,70	10163,30	494,7508	5	8773,15	864,3	8643,498
			9918,80						
			9838,40						
