# Supplementary material for: Visualizing increased uptake of [18F]FDG and [18F]FTHA in kidneys from obese high-fat diet fed C57BL/6J mice using PET/CT ex vivo
Source: PLoS One. 2023 Feb 14;18(2):e0281705. doi: 10.1371/journal.pone.0281705 (PMC9928095; doi:10.1371/journal.pone.0281705)
Supplement: S4 Data — (PDF) [file pone.0281705.s004.pdf]

Insulin

	abs 450nm		ng/ml		
	load 1µl	load 5µl	load 1µl	load 5µl	
#1	0,3613	1,6726	1,35	6,08	
#2	0,0669	0,2414	0,22	0,89	Is very low compared to cage mates! Also low in weight and in BG?
#3	1,2119	3,6957	4,42	15,18	
#4	0,5947	2,3363	2,20	8,63	
#5	0,2848	1,2237	1,06	4,46	
#6	0,0594	0,2397	0,20	0,89	
#7	0,1178	0,5239	0,42	1,95	
#8	0,1259	0,5526	0,45	2,05	
#9	0,0787	0,3851	0,27	1,43	
#10	0,1044	0,4996	0,37	1,86	

NEFA

Brunn	sample	Diet	546 nm	546 nm	(-) blank Mean 546	660 nm	660 nm	(-) blank Mean 660	Final nm	mmol/l
1	1	HFD	0,141	0,143	0,082	0,051	0,051	0,008	0,074	0,560234
2	2		0,152		0,092	0,051		0,008	0,084	0,637117
3	3		0,139	0,139	0,079	0,052	0,051	0,0085	0,0705	0,533325
4	4		0,125	0,13	0,0675	0,049	0,052	0,0075	0,06	0,452598
5	5		0,122	0,142	0,072	0,048	0,054	0,008	0,064	0,483351
6	6	Chow old								
7	7		0,204	0,22	0,152	0,059	0,062	0,0175	0,1345	1,025376
8	8		0,237	0,264	0,1905	0,06	0,067	0,0205	0,17	1,298311
9	9		0,203		0,143	0,056		0,013	0,13	0,990779
10	10		0,231	0,232	0,1715	0,064	0,064	0,021	0,1505	1,148389
11	7029	Chow you	0,159	0,149	0,094	0,063	0,052	0,0145	0,0795	0,60252
12	7030		0,172	0,176	0,114	0,054	0,057	0,0125	0,1015	0,771662
13	7031		0,202	0,201	0,1415	0,058	0,058	0,015	0,1265	0,96387
14	7038		0,23	0,228	0,169	0,062	0,061	0,0185	0,1505	1,148389
15	7039		0,185	0,188	0,1265	0,057	0,057	0,014	0,1125	0,856234
16	7040		0,188	0,186	0,127	0,056	0,055	0,0125	0,1145	0,87161

40 weeks old mice

22 wks on HFD

Diet	Nr	27-jan BW (g)	B-Glc	ng/ml load 1µl	load 5µl
HFD	1	63,1	10,7	1,345358	6,079661
	2	44,3	10,9	0,224956	0,894793
	3	54	12,8	4,416148	15,17518
	4	59,9	12,7	2,204458	8,633828
	5	56,5	13	1,058717	4,458266
Chow	6	33,9	7,7	0,195748	0,888352
	7	37,9	10	0,422231	1,94602
	8	40,4	10,3	0,453475	2,050985
	9	41	9,5	0,270836	1,433973
	10	37	10,9	0,370454	1,856919

Diet	Nr	insulin ng/	insulin mi	glucose m	HOMA-IR	ug/ml NEFA
HFD	1	1,345358	33,39011	10,7	15,87885	157,2
	2	0,224956	5,583133	10,9	2,704718	178,6
	3	4,416148	109,6033	12,8	62,3521	151,3
	4	2,204458	54,71191	12,7	30,88183	127,7
	5	1,058717	26,27604	13	15,18171	134
Chow	6	0,195748	4,858226	7,7	1,662593	
	7	0,422231	10,47924	10	4,657442	291,1
	8	0,453475	11,25468	10,3	5,152143	367
	9	0,270836	6,721822	9,5	2,838102	280,3
	10	0,370454	9,194215	10,9	4,454086	325,7
