# Supplementary material for: Visualizing increased uptake of [18F]FDG and [18F]FTHA in kidneys from obese high-fat diet fed C57BL/6J mice using PET/CT ex vivo
Source: PLoS One. 2023 Feb 14;18(2):e0281705. doi: 10.1371/journal.pone.0281705 (PMC9928095; doi:10.1371/journal.pone.0281705)
Supplement: S5 Data — (PDF) [file pone.0281705.s005.pdf]

Kidney 2018-12-04

cDNA-Number	samples: Mouse nr	Age	Diet	Nut.stat	conc. [µg/µl] RNA [ng/µl]	4000 ng motsvarar:	H_2O add 10 µl
1	4409	Young	Chow	Fasted	803,926	**4,98**	**5,02**
2	4468				712,263	**5,62**	**4,38**
3	4487				744,095	**5,38**	**4,62**
4	4478			Adlib	814,813	**4,91**	**5,09**
5	4910				747,291	**5,35**	**4,65**
6	4911				1069,662	**3,74**	**6,26**
7	4470	Young	Chow	Adlib	1009,099	**3,96**	**6,04**
8	4475				1252,438	**3,19**	**6,81**
9	4476				657,76	**6,08**	**3,92**
10	4488			Fasted	1033,027	**3,87**	**6,13**
11	4930				877,944	**4,56**	**5,44**
12	4932				985,928	**4,06**	**5,94**
13	3935	Old	Chow	Fasted	1033,238	**3,87**	**6,13**
14	3938				801,361	**4,99**	**5,01**
15	4461				1141,271	**3,50**	**6,50**
16	4462				1113,093	**3,59**	**6,41**
17	4464				1171,95	**3,41**	**6,59**
18	B71				1049,231	**3,81**	**6,19**
19	3931	Old	Chow	Adlib	952,272	**4,20**	**5,80**
20	3933				1068,981	**3,74**	**6,26**
21	4459				875,437	**4,57**	**5,43**
22	4460				1151,639	**3,47**	**6,53**
23	4466				848,669	**4,71**	**5,29**
24	B83				1098,852	**3,64**	**6,36**
25	4366	Short	HFD	Fasted	1227,931	**3,26**	**6,74**
26	4367				765,613	**5,22**	**4,78**
27	4354				1311,969	**3,05**	**6,95**
28	4302	Short	HFD	Adlib	1008,115	**3,97**	**6,03**
29	4352				1235,625	**3,24**	**6,76**
30	4364				944,512	**4,23**	**5,77**
31	4365				858,15	**4,66**	**5,34**
32	3929	Long	HFD	Fasted	844,948	**4,73**	**5,27**
33	3934				768,998	**5,20**	**4,80**
34	4166				946,854	**4,22**	**5,78**
35	4484				992,545	**4,03**	**5,97**
36	4485				1760,456	**2,27**	**7,73**
37	4486				1234,723	**3,24**	**6,76**
38	3939	Long	HFD	Adlib	727,317	**5,50**	**4,50**
39	4167				558,085	**7,17**	**2,83**
40	4169				1046,141	**3,82**	**6,18**
41	4472				983,505	**4,07**	**5,93**
42	4413				816,669	**4,90**	**5,10**
43	4414				1242,475	**3,22**	**6,78**

Date:2018-12-04

**Reverse transcription:**	**RT+**	
Number of samples	43	
Number of control RNA	0	
Margin of safety	1,04	
Total of tubes to prepare	44,72	
	volume / tube	**total volume to prepare**
5xRT buffer (5X)	4,00 µl	**178,88 µl**
dNTPs Mix (10mM)	2,00 µl	**89,44 µl**
Random primers (p(dN)6)	1,00 µl	**44,72 µl**
transcriptase (200U/µL)	1,00 µl	**44,72 µl**
Rnase Inhibitor [Rnasin], 40 U/µl	0,20 µl	**8,94 µl**
Nuclease-free (DEPC-treated) H_2O	1,80 µl	**80,50 µl**
Sub-total	**10,00 µl**	**447,20 µl**
Sample RNAs	**10 µl**	
**Total**	**20 µl**	
**Programm.Nummer:**	10 min	25 °C
**Direction 5, Programm 1**	60 min	42 °C
	10 min	70 °C
		4 °C

2018-12-05

AoD	assay-#	Lot
18S	Mm03928990_g1	

	1	2	3	4	5	6	7	8	9	10	11	12
A	1	1	9	9	17	17	25	25	33	33	41	41
B	2	2	10	10	18	18	26	26	34	34	42	42
C	3	3	11	11	19	19	27	27	35	35	43	43
D	4	4	12	12	20	20	28	28	36	36	nc	nc
E	5	5	13	13	21	21	29	29	37	37		
F	6	6	14	14	22	22	30	30	38	38		
G	7	7	15	15	23	23	31	31	39	39		
H	8	8	16	16	24	24	32	32	40	40		

Preparation of mastermix for duplicates (18S)

		MM
No samples	44,3	32
Sample copy	2	
universal mix [2x]	10	886
AoD	1	88,6
Nuclease free-H2O	8	708,8
50ng cDNA/well	1	
Total (ul)	20	1683,4

Kidney

18S

	Diet	Nutstat	Mouse	Sample	Ct	Mean	SD mean	Group mea	SD group
			4409	1	5,432269	5,60	0,23	5,4	0,15

				1	5,760146				
			4468	2	5,630312	5,61	0,03		
		Fasted		2	5,588641				
			4487	3	5,312489	5,42	0,16		
				3	5,535698				
			4478	4	5,305687	5,38	0,10		
				4	5,452591				
		Adlib	4910	5	5,539769	5,44	0,14		
				5	5,345171				
			4911	6	4,990819	5,22	0,32		
Young	Chow			6	5,449184				
			4470	7	5,531734	5,50	0,04	5,4	0,20
				7	5,469159				
			4475	8	4,910907	5,03	0,17		
		Adlib		8	5,156791				
			4476	9	5,672322	5,64	0,05		
				9	5,607431				
			4488	10	5,143156	5,34	0,27		
				10	5,529506				
		Fasted	4930	11	5,253589	5,36	0,16		
				11	5,47638				
			4932	12	5,391278	5,33	0,09		
				12	5,265903				
			3935	13	5,49576	5,34	0,22	5,4	0,13
				13	5,181608				
			3938	14	5,474171	5,46	0,03		
				14	5,436412				
			4461	15	5,139677	5,20	0,08		
		Fasted		15	5,250623				
			4462	16	5,579175	5,60	0,02		
				16	5,61167				
			4464	17	5,422245	5,44	0,03		
				17	5,464609				

Old	Chow		B71	18	5,416392	5,41	0,00		
				18	5,412723				
		Adlib	3931	19	5,350445	5,47	0,16	5,3	0,12
				19	5,57977				
			3933	20	5,258956	5,30	0,06		
				20	5,340473				
			4459	21	4,773988	5,14	0,51		
				21	5,498133				
			4460	22	5,548725	5,45	0,14		
				22	5,346066				
			4466	23	5,354516	5,24	0,16		
				23	5,127038				
			B83	24	5,244211	5,34	0,13		
				24	5,427874				
Short	HFD	Fasted	4366	25	5,505247	5,55	0,06	5,6	0,1
				25	5,588733				
			4367	26	5,760902	5,61	0,22		
				26	5,451983				
			4354	27	5,546674	5,68	0,20		
				27	5,822748				
		Adlib	4302	28	5,583893	5,51	0,10	5,4	0,1
				28	5,435683				
			4352	29	5,334853	5,42	0,12		
				29	5,50142				
			4364	30	5,64744	5,50	0,20		
				30	5,360463				
			4365	31	5,150175	5,19	0,06		
				31	5,236461				
			3929	32	5,357338	5,36	0,00	5,5	0,15
				32	5,353753				
			3934	33	5,082292	5,31	0,33		
				33	5,542487				
			4166	34	5,506898	5,51	0,01		

Long	HFD	Fasted		34	5,517481				
			4484	35	5,644696	5,62	0,04		
				35	5,588171				
			4485	36	5,561972	5,68	0,16		
				36	5,794336				
			4486	37	5,604755	5,56	0,06		
				37	5,524317				
		Adlib	3939	38	5,594411	5,51	0,11	5,5	0,18
				38	5,433467				
			4167	39	5,294457	5,48	0,26		
				39	5,662885				
			4169	40	5,14567	5,22	0,11		
				40	5,29426				
			4472	41	5,481658	5,42	0,09		
				41	5,360525				
			4413	42	5,940207	5,75	0,27		
				42	5,557223				
			4414	43	5,47714	5,60	0,18		
				43	5,72817				

2018-12-06

AoD	assay-#	Lot
Slc2a1	Mm00441480_m1	

	1	2	3	4	5	6	7	8	9	10	11	12
A	1	1	9	9	17	17	25	25	33	33	41	41
B	2	2	10	10	18	18	26	26	34	34	42	42
C	3	3	11	11	19	19	27	27	35	35	43	43
D	4	4	12	12	20	20	28	28	36	36	nc	nc
E	5	5	13	13	21	21	29	29	37	37		
F	6	6	14	14	22	22	30	30	38	38		
G	7	7	15	15	23	23	31	31	39	39		
H	8	8	16	16	24	24	32	32	40	40		

Preparation of mastermix for duplicates

		MM
No samples	44,3	32
Sample copy	2	
universal mix [2x]	10	886
AoD	1	88,6
Nuclease free-H2O	8	708,8
50ng cDNA/well	1	
Total (ul)	20	1683,4

Relative to young chow fasted

Kidney

GLUT1										GLUT1					
	Diet	Nutstat	Sample	Mouse	Ct	Mean	SD mean	Group mea	SD group	18S	dCt	mean of d(	DDct	2^DDct	mean of 2^DDct
Young	Chow	Fasted	1	4409	21,82124	21,9	0,1	21,8	0,4	5,60	16,3	16,5348	-0,23	1,18	1,02
			1		21,97481										
			2	4468	22,25664	22,3	0,1			5,61	16,7		0,17	0,89	
			2		22,3676										
			3	4487	21,78278	21,8	0,0			5,42	16,4		-0,16	1,12	
			3		21,82019										
		Adlib	4	4478	21,69145	21,7	0,1			5,38	16,4		-0,18	1,13	
			4		21,77701										
			5	4910	21,78581	21,8	0,0			5,44	16,3		-0,21	1,15	
			5		21,75427										
			6	4911	21,12734	21,2	0,1			5,22	16,0		-0,55	1,46	
			6		21,2822										
		Adlib	7	4470	21,73385	21,8	0,1	21,8	0,3	5,50	16,3	16,3	-0,25	1,19	1,23
			7		21,83849										
			8	4475	21,61982	21,8	0,3			5,03	16,8		0,26	0,84	
			8		22,02979										
			9	4476	21,4878	21,5	0,0			5,64	15,9		-0,67	1,59	
			9		21,51687										
		Fasted	10	4488	22,3121	22,3	0,1			5,34	16,9		0,40	0,76	
			10		22,2376										
			11	4930	22,09625	22,1	0,1			5,36	16,7		0,15	0,90	
			11		22,00857										
			12	4932	21,48148	21,5	0,1			5,33	16,2		-0,33	1,26	
			12		21,57722										
Old	Chow	Fasted	13	3935	22,30957	22,4	0,1	22,0	0,3	5,34	17,0	16,6	0,50	0,71	1,01
			13		22,43765										
			14	3938	21,74469	21,7	0,1			5,46	16,3		-0,28	1,22	
			14		21,66692										
			15	4461	22,21491	22,2	0,0			5,20	17,0		0,51	0,70	
			15		22,26953										
			16	4462	21,91777	22,0	0,1			5,60	16,4		-0,13	1,09	
			16		22,0901										
			17	4464	21,6555	21,6	0,1			5,44	16,1		-0,40	1,32	
			17		21,50923										
			18	B71	21,89524	21,9	0,1			5,41	16,5		-0,01	1,00	
			18		21,99331										
		Adlib	19	3931	21,35187	21,4	0,1	21,6	0,1	5,47	15,9	16,3	-0,59	1,50	1,19
			19		21,47567										
			20	3933	21,74227	21,7	0,0			5,30	16,4		-0,09	1,06	
			20		21,75542										
			21	4459	21,38799	21,6	0,2			5,14	16,4		-0,11	1,08	
			21		21,7312										
			22	4460	21,60069	21,7	0,2			5,45	16,3		-0,27	1,20	
			22		21,83036										
			23	4466	21,64879	21,7	0,0			5,24	16,4		-0,11	1,08	
			23		21,67415										
			24	B83	21,56243	21,6	0,0			5,34	16,2		-0,30	1,23	
			24		21,57987										
Short	HFD	Fasted	25	4366	21,63519	21,6	0,0	21,7	0,1	5,55	16,1	16,1	-0,48	1,39	1,40
			25		21,57457										
			26	4367	21,76543	21,8	0,0			5,61	16,1		-0,39	1,31	
			26		21,73892										
			27	4354	21,59689	21,6	0,1			5,68	16,0		-0,57	1,49	
			27		21,6926										
		Adlib	28	4302	21,8781	21,9	0,0	21,8	0,1	5,51	16,4	16,4	-0,15	1,11	1,10
			28		21,92004										
			29	4352	21,6911	21,7	0,0			5,42	16,3		-0,27	1,21	
			29		21,6707										
			30	4364	21,69186	21,7	0,1			5,50	16,2		-0,30	1,23	
			30		21,78058										
			31	4365	21,93253	22,0	0,0			5,19	16,8		0,23	0,86	
			31		21,97563										
Long	HFD	Fasted	32	3929	21,96277	21,7	0,3	21,8	0,2	5,36	16,4	16,3	-0,15	1,11	1,18
			32		21,51355										
			33	3934	21,65873	21,6	0,0			5,31	16,3		-0,21	1,15	
			33		21,62406										
			34	4166	21,73371	21,7	0,0			5,51	16,2		-0,30	1,23	
			34		21,76162										
			35	4484	21,84871	21,9	0,0			5,62	16,2		-0,30	1,23	
			35		21,85244										
			36	4485	22,15951	22,1	0,1			5,68	16,4		-0,11	1,08	
			36		22,04579										
			37	4486	21,75958	21,8	0,0			5,56	16,2		-0,33	1,26	
			37		21,77716										
		Adlib	38	3939	21,72126	21,8	0,0	21,7	0,3	5,51	16,2	16,2	-0,29	1,23	1,25
			38		21,78697										
			39	4167	21,93088	21,9	0,0			5,48	16,5		-0,08	1,05	
			39		21,94263										
			40	4169	21,38464	21,5	0,1			5,22	16,2		-0,29	1,23	
			40		21,53626										
			41	4472	21,52897	21,6	0,1			5,42	16,2		-0,38	1,30	
			41		21,62588										
			42	4413	22,22485	22,3	0,1			5,75	16,5		0,00	1,00	
			42		22,33553										
			43	4414	21,42402	21,4	0,1			5,60	15,8		-0,75	1,69	
			43		21,34388										

2018-12-06

AoD	assay-#	Lot
GLUT2	Mm00453831_m1	

	1	2	3	4	5	6	7	8	9	10	11	12
A	1	1	9	9	17	17	25	25	33	33	41	41
B	2	2	10	10	18	18	26	26	34	34	42	42
C	3	3	11	11	19	19	27	27	35	35	43	43
D	4	4	12	12	20	20	28	28	36	36	nc	nc
E	5	5	13	13	21	21	29	29	37	37		
F	6	6	14	14	22	22	30	30	38	38		
G	7	7	15	15	23	23	31	31	39	39		
H	8	8	16	16	24	24	32	32	40	40		

Preparation of mastermix for duplicates (GLUT2)

		MM
No samples	44,3	32
Sample copy	2	
universal mix [2x]	10	886
AoD	1	88,6
Nuclease free-H2O	8	708,8
50ng cDNA/well	1	
Total (ul)	20	1683,4

Relative to young chow fasted

Kidney

GLUT2										GLUT2					
	Diet	Nutstat	Sample	Mouse	Ct	Mean	SD mean	Group mea	SD group	18S	dCt	mean of d(	DDct	2^DDct	mean of 2^DDct
			1	4409	23,40872	23,4	0,0	23,4	0,2	5,60	17,8	17,8494	-0,02	1,01	1,01
			1		23,44345										
		Fasted	2	4468	23,19561	23,3	0,2			5,61	17,7		-0,13	1,10	
			2		23,45898										
			3	4487	23,60178	23,6	0,0			5,42	18,2		0,34	0,79	
			3		23,61934										
			4	4478	22,95088	23,0	0,0			5,38	17,6		-0,26	1,20	
			4		22,98174										
		Adlib	5	4910	23,24904	23,5	0,4			5,44	18,1		0,22	0,86	
			5		23,77709										
			6	4911	23,42531	23,4	0,0			5,22	18,2		0,36	0,78	
Young	Chow		6		23,4346										
			7	4470	23,12007	23,2	0,1	23,2	0,1	5,50	17,7	17,9	-0,15	1,11	1,00
			7		23,2777										
		Adlib	8	4475	22,92923	23,0	0,1			5,03	18,0		0,14	0,91	
			8		23,10896										
			9	4476	23,1391	23,3	0,3			5,64	17,7		-0,17	1,12	
			9		23,49969										
			10	4488	23,29171	23,2	0,1			5,34	17,9		0,03	0,98	

			10		23,148										
		Fasted	11	4930	23,01783	23,0	0,0			5,36	17,7		-0,18	1,13	
			11		23,04832										
			12	4932	23,07897	23,1	0,1			5,33	17,8		-0,04	1,03	
			12		23,19918										
			13	3935	23,32088	23,4	0,1	23,3	0,2	5,34	18,0	17,9	0,18	0,88	0,97
			13		23,41251										
			14	3938	23,46939	23,5	0,0			5,46	18,0		0,15	0,90	
			14		23,43482										
			15	4461	23,06842	23,0	0,1			5,20	17,8		-0,04	1,03	
		Fasted	15		22,94529										
			16	4462	23,18272	23,2	0,1			5,60	17,7		-0,20	1,15	
			16		23,31037										
			17	4464	23,44066	23,4	0,1			5,44	17,9		0,10	0,93	
			17		23,34579										
			18	B71	23,42721	23,4	0,1			5,41	18,0		0,12	0,92	
Old	Chow		18		23,33654										
			19	3931	23,72567	23,7	0,0	23,3	0,2	5,47	18,3	18,0	0,43	0,74	0,90
			19		23,76172										
			20	3933	23,25773	23,2	0,1			5,30	17,9		0,05	0,97	
			20		23,14184										
			21	4459	23,30519	23,5	0,2			5,14	18,3		0,49	0,71	
		Adlib	21		23,63852										
			22	4460	23,20569	23,2	0,0			5,45	17,8		-0,09	1,07	
			22		23,20509										
			23	4466	23,21621	23,2	0,1			5,24	17,9		0,08	0,94	
			23		23,1275										
			24	B83	23,13695	23,2	0,1			5,34	17,9		0,01	0,99	
			24		23,25563										
			25	4366	23,10319	23,2	0,1	22,9	0,2	5,55	17,6		-0,24	1,18	1,48
			25		23,21463										
		Fasted	26	4367	22,81377	22,9	0,1			5,61	17,2		-0,61	1,52	
			26		22,88726										
			27	4354	22,76431	22,7	0,0			5,68	17,1		-0,80	1,74	
			27		22,7084										
Short	HFD		28	4302	22,83459	23,1	0,3	23,0	0,2	5,51	17,6		-0,29	1,22	1,18
			28		23,31165										
			29	4352	22,79325	22,8	0,0			5,42	17,4		-0,46	1,38	
		Adlib	29		22,81444										
			30	4364	23,15447	23,2	0,1			5,50	17,7		-0,15	1,11	
			30		23,26033										
			31	4365	23,00161	23,0	0,1			5,19	17,8		0,00	1,00	
			31		23,08238										
			32	3929	23,60888	23,6	0,1	23,2	0,3	5,36	18,2	17,7	0,37	0,78	1,16
			32		23,53599										
			33	3934	23,22529	23,3	0,1			5,31	18,0		0,14	0,91	
			33		23,37787										
			34	4166	23,30611	23,3	0,0			5,51	17,8		-0,07	1,05	
		Fasted	34		23,28053										
			35	4484	22,65483	22,6	0,0			5,62	17,0		-0,84	1,78	

			35		22,60669										
			36	4485	23,39689	23,4	0,0			5,68	17,7		-0,15	1,11	
			36		23,35749										
			37	4486	23,0253	23,0	0,0			5,56	17,4		-0,40	1,32	
Long	HFD		37		23,00184										
			38	3939	23,33638	23,3	0,1	23,0	0,3	5,51	17,8	17,5	-0,07	1,05	1,30
			38		23,24222										
			39	4167	23,20133	23,2	0,0			5,48	17,7		-0,14	1,10	
			39		23,16997										
			40	4169	22,92819	22,9	0,0			5,22	17,7		-0,15	1,11	
		Adlib	40		22,9029										
			41	4472	22,62032	22,6	0,1			5,42	17,2		-0,69	1,61	
			41		22,54438										
			42	4413	22,73981	22,8	0,1			5,75	17,1		-0,79	1,73	
			42		22,86836										
			43	4414	23,22519	23,2	0,1			5,60	17,6		-0,28	1,21	
			43		23,12859										

AoD	assay-#	Lot
SGLT2	Mm00453831_m1	

	1	2	3	4	5	6	7	8	9	10	11	12
A	1	1	9	9	17	17	25	25	33	33	41	41
B	2	2	10	10	18	18	26	26	34	34	42	42
C	3	3	11	11	19	19	27	27	35	35	43	43
D	4	4	12	12	20	20	28	28	36	36	nc	nc
E	5	5	13	13	21	21	29	29	37	37		
F	6	6	14	14	22	22	30	30	38	38		
G	7	7	15	15	23	23	31	31	39	39		
H	8	8	16	16	24	24	32	32	40	40		

Preparation of mastermix for duplicates (SGLT2)

		MM
No sample:	44,3	32
Sample cop	2	
niversal mix [2x	10	886
AoD	1	88,6
uclease free-H2	8	708,8
50ng cDNA/well	1	
Total (ul)	20	1683,4

Relative to young chow fasted

Kidney

SGLT2										SGLT2					
	Diet	Nutstat	Sample	Mouse	Ct	Mean	SD mean	Group mea	SD group	18S	dCt	mean of d(	DDct	2^DDct	mean of 2^DDct
			1	4409	21,38129	21,4	0,0	21,3	0,2	5,60	15,8	15,7068	0,10	0,93	1,01
			1		21,42243										
		Fasted	2	4468	20,96818	21,0	0,1			5,61	15,4		-0,28	1,21	
			2		21,10656										
			3	4487	21,22157	21,2	0,0			5,42	15,8		0,09	0,94	
			3		21,21927										
			4	4478	21,15418	21,1	0,0			5,38	15,8		0,04	0,97	
			4		21,1065										
		Adlib	5	4910	21,37002	21,6	0,3			5,44	16,1		0,40	0,76	
			5		21,73704										
			6	4911	21,25962	21,3	0,1			5,22	16,1		0,39	0,76	
Young	Chow		6		21,38014										
			7	4470	20,79066	20,9	0,1	21,0	0,3	5,50	15,4	15,8	-0,33	1,26	0,95
			7		20,95851										
		Adlib	8	4475	20,46505	20,7	0,4			5,03	15,7		-0,03	1,02	
			8		20,9612										
			9	4476	21,43253	21,5	0,1			5,64	15,8		0,12	0,92	
			9		21,50536										
			10	4488	20,91669	20,9	0,0			5,34	15,6		-0,11	1,08	

			10		20,9576										
		Fasted	11	4930	21,11688	21,2	0,1			5,36	15,8		0,12	0,92	
			11		21,268										
			12	4932	21,08799	21,1	0,0			5,33	15,8		0,08	0,95	
			12		21,13487										
			13	3935	21,49786	21,5	0,0	21,4	0,2	5,34	16,2	16,0	0,46	0,73	0,85
			13		21,50552										
			14	3938	21,43658	21,5	0,0			5,46	16,0		0,29	0,82	
			14		21,47097										
			15	4461	21,16436	21,3	0,2			5,20	16,1		0,38	0,77	
		Fasted	15		21,40108										
			16	4462	21,12441	21,2	0,1			5,60	15,6		-0,13	1,10	
			16		21,21537										
			17	4464	21,50718	21,5	0,0			5,44	16,1		0,39	0,76	
			17		21,56872										
			18	B71	21,196	21,2	0,0			5,41	15,8		0,10	0,93	
Old	Chow		18		21,24124										
			19	3931	21,7953	21,8	0,0	21,3	0,3	5,47	16,4	16,0	0,65	0,64	0,81
			19		21,85022										
			20	3933	21,12765	21,1	0,0			5,30	15,9		0,14	0,91	
			20		21,17227										
			21	4459	21,10141	21,2	0,2			5,14	16,1		0,37	0,77	
		Adlib	21		21,3341										
			22	4460	21,16719	21,2	0,0			5,45	15,7		0,01	0,99	
			22		21,16761										
			23	4466	21,2606	21,3	0,1			5,24	16,1		0,35	0,79	
			23		21,3325										
			24	B83	21,18144	21,4	0,3			5,34	16,0		0,32	0,80	
			24		21,54923										
			25	4366	21,99266	22,0	0,0	21,8	0,3	5,55	16,4		0,74	0,60	0,75
			25		21,9913										
		Fasted	26	4367	21,82119	21,9	0,0			5,61	16,2		0,54	0,69	
			26		21,88779										
			27	4354	21,4135	21,4	0,0			5,68	15,7		0,04	0,97	
			27		21,45437										
			28	4302	21,35336	21,4	0,0	21,4	0,0	5,51	15,9		0,16	0,90	0,82
Short	HFD		28		21,39341										
			29	4352	21,48784	21,5	0,0			5,42	16,0		0,33	0,79	
		Adlib	29		21,42881										
			30	4364	21,42001	21,4	0,0			5,50	15,9		0,20	0,87	
			30		21,4039										
			31	4365	21,33164	21,4	0,1			5,19	16,2		0,47	0,72	
			31		21,40559										
			32	3929	21,72674	21,6	0,2	21,6	0,1	5,36	16,2	16,1	0,51	0,70	0,79
			32		21,4208										
			33	3934	21,32687	21,4	0,2			5,31	16,1		0,42	0,75	
			33		21,54554										
			34	4166	21,47572	21,5	0,0			5,51	16,0		0,27	0,83	
			34		21,50974										
		Fasted	35	4484	21,36903	21,5	0,1			5,62	15,8		0,14	0,91	

			35		21,55966										
			36	4485	21,81555	21,8	0,0			5,68	16,1		0,42	0,75	
			36		21,79576										
			37	4486	21,5841	21,6	0,0			5,56	16,0		0,32	0,80	
Long	HFD		37		21,59012										
			38	3939	21,5105	21,5	0,0	21,5	0,2	5,51	16,0	16,0	0,30	0,81	0,84
			38		21,52965										
			39	4167	21,4632	21,5	0,0			5,48	16,0		0,30	0,81	
			39		21,50485										
			40	4169	21,08496	21,1	0,1			5,22	15,9		0,20	0,87	
		Adlib	40		21,16869										
			41	4472	21,31846	21,3	0,0			5,42	15,9		0,18	0,88	
			41		21,3064										
			42	4413	21,5484	21,6	0,0			5,75	15,8		0,10	0,93	
			42		21,56136										
			43	4414	21,73392	21,8	0,0			5,60	16,2		0,45	0,73	
			43		21,79224										

2018-12-06

AoD	assay-#	Lot
Slc27a2	Mm00449517_m1	

	1	2	3	4	5	6	7	8	9	10	11	12
A	1	1	9	9	17	17	25	25	33	33	41	41
B	2	2	10	10	18	18	26	26	34	34	42	42
C	3	3	11	11	19	19	27	27	35	35	43	43
D	4	4	12	12	20	20	28	28	36	36	nc	nc
E	5	5	13	13	21	21	29	29	37	37		
F	6	6	14	14	22	22	30	30	38	38		
G	7	7	15	15	23	23	31	31	39	39		
H	8	8	16	16	24	24	32	32	40	40		

Preparation of mastermix for duplicates

		MM
No samples	44,3	32
Sample copy	2	
universal mix [2x]	10	886
AoD	1	88,6
Nuclease free-H2O	8	708,8
50ng cDNA/well	1	
Total (ul)	20	1683,4

Relative to young chow fasted

Kidney

			FATP2								FATP2					
	Diet	Nutstat	Sample	Mouse	Ct	Mean	SD mean	Group mea	SD group	18S	dCt	mean of d(	DDct	2^DDct	mean of 2^DDct
Young	Chow	Fasted	1	4409	14,08294	14,3	0,2	14,2	0,2	5,60	8,7	8,6086	0,05	0,97	1,00
			1		14,42868										
			2	4468	14,15122	14,2	0,1			5,61	8,6		0,01	0,99	
			2		14,30241										
			3	4487	13,95598	14,0	0,1			5,42	8,6		-0,04	1,02	
			3		14,03845										
		Adlib	4	4478	14,25142	14,1	0,2			5,38	8,7		0,12	0,92	
			4		13,95416										
			5	4910	14,45399	14,5	0,0			5,44	9,0		0,42	0,75	
			5		14,49613										
			6	4911	14,15881	14,2	0,1			5,22	9,0		0,40	0,76	
			6		14,29619										
		Adlib	7	4470	14,14732	14,3	0,2	14,1	0,5	5,50	8,8	8,9	0,17	0,89	0,83
			7		14,40214										
			8	4475	13,05491	13,5	0,6			5,03	8,5		-0,15	1,11	
			8		13,93029										
			9	4476	14,93221	15,0	0,1			5,64	9,4		0,78	0,58	
			9		15,13275										

			10	4488	14,10901	14,1	0,0			5,34	8,8		0,16	0,89	
			10		14,10193										
		Fasted	11	4930	13,92935	13,7	0,3			5,36	8,4		-0,23	1,18	
			11		13,54886										
			12	4932	13,78288	14,0	0,3			5,33	8,7		0,05	0,97	
			12		14,1914										
			13	3935	13,85698	13,9	0,1	13,9	0,2	5,34	8,6	8,5	-0,04	1,03	1,05
			13		13,95983										
			14	3938	14,21632	14,1	0,2			5,46	8,6		0,03	0,98	
			14		13,97083										
			15	4461	13,66819	13,7	0,0			5,20	8,5		-0,13	1,10	
		Fasted	15		13,67328										
			16	4462	14,03107	14,0	0,1			5,60	8,4		-0,22	1,17	
			16		13,93209										
			17	4464	13,78226	13,9	0,1			5,44	8,4		-0,18	1,13	
			17		13,96306										
			18	B71	14,13661	14,2	0,0			5,41	8,7		0,14	0,91	
Old	Chow		18		14,18604										
			19	3931	14,2874	14,4	0,2	14,1	0,2	5,47	8,9	8,8	0,33	0,80	0,87
			19		14,51232										
			20	3933	14,19791	14,2	0,0			5,30	8,9		0,29	0,82	
			20		14,19883										
			21	4459	13,03685	13,7	1,0			5,14	8,6		0,01	1,00	
		Adlib	21		14,46296										
			22	4460	13,88158	14,1	0,2			5,45	8,6		0,00	1,00	
			22		14,23083										
			23	4466	14,38609	14,3	0,1			5,24	9,1		0,48	0,72	
			23		14,26466										
			24	B83	13,90808	14,1	0,3			5,34	8,8		0,20	0,87	
			24		14,38384										
			25	4366	13,82804	13,9	0,0	13,7	0,3	5,55	8,3	8,1	-0,30	1,23	1,46
			25		13,88608										
		Fasted	26	4367	13,31859	13,4	0,1			5,61	7,8		-0,82	1,76	
			26		13,47928										
			27	4354	13,86641	13,8	0,0			5,68	8,1		-0,46	1,38	
			27		13,79591										
Short	HFD		28	4302	13,7674	14,0	0,3	14,1	0,2	5,51	8,5	8,7	-0,11	1,08	0,94
			28		14,25823										
			29	4352	14,63103	14,5	0,2			5,42	9,1		0,44	0,73	
		Adlib	29		14,3115										
			30	4364	14,13797	13,9	0,3			5,50	8,4		-0,20	1,15	
			30		13,68703										
			31	4365	14,21296	14,1	0,1			5,19	8,9		0,32	0,80	
			31		14,03378										
			32	3929	13,93037	13,9	0,0	14,2	0,3	5,36	8,6	8,7	-0,03	########	########
			32		13,93912										
			33	3934	13,82654	13,9	0,1			5,31	8,6		0,01	0,99	
			33		14,03507										
			34	4166	13,92911	14,0	0,0			5,51	8,5		-0,16	1,11	
		Fasted	34		13,99921										

		Fasted	35	4484	13,83862	14,2	0,5			5,62	8,6		-0,05	1,03	
			35		14,51451										
			36	4485	14,74504	14,8	0,0			5,68	9,1		0,48	0,72	
			36		14,78727										
			37	4486	14,4541	14,4	0,1			5,56	8,8		0,23	0,85	
Long	HFD		37		14,35742										
			38	3939	14,58721	14,5	0,2	14,1	0,3	5,51	9,0	8,7	0,34	0,79	0,98
			38		14,34284										
			39	4167	14,61588	14,5	0,2			5,48	9,0		0,37	0,77	
			39		14,30604										
			40	4169	13,75785	13,9	0,2			5,22	8,7		0,08	0,95	
		Adlib	40		14,05253										
			41	4472	14,00167	14,0	0,0			5,42	8,6		-0,05	1,04	
			41		13,95588										
			42	4413	13,8686	14,1	0,4			5,75	8,4		-0,23	1,17	
			42		14,38095										
			43	4414	14,12035	14,0	0,2			5,60	8,4		-0,25	1,19	
			43		13,80939										

2018-12-05

AoD	assay-#	Lot
Cd36	Mm00432403_m1	

	1	2	3	4	5	6	7	8	9	10	11	12
A	1	1	9	9	17	17	25	25	33	33	41	41
B	2	2	10	10	18	18	26	26	34	34	42	42
C	3	3	11	11	19	19	27	27	35	35	43	43
D	4	4	12	12	20	20	28	28	36	36	nc	nc
E	5	5	13	13	21	21	29	29	37	37		
F	6	6	14	14	22	22	30	30	38	38		
G	7	7	15	15	23	23	31	31	39	39		
H	8	8	16	16	24	24	32	32	40	40		

Preparation of mastermix for duplicates (Cd36)

		MM
No samples	44,3	32
Sample copy	2	
universal mix [2x]	10	886
AoD	1	88,6
Nuclease free-H2O	8	708,8
50ng cDNA/well	1	
Total (ul)	20	1683,4

Relative to young chow fasted

Kidney

Cd36

	Diet	Nutstat	Sample	Mouse	Ct	Mean	SD mean	Group mea	SD group	18S	dCt	mean of d(	DDct	2^DDct	mean of 2^DDct
Young	Chow	Fasted	1	4409	20,566	20,6	0,0	20,8	0,3	5,60	15,0	15,3230	-0,33	1,25	1,02
			1		20,62144										
			2	4468	20,56557	20,7	0,3			5,61	15,1		-0,19	1,14	
			2		20,92121										
			3	4487	20,95408	21,0	0,0			5,42	15,5		0,21	0,86	
			3		20,95934										
		Adlib	4	4478	21,09866	21,2	0,1			5,38	15,8		0,47	0,72	
			4		21,23827										
			5	4910	20,62045	20,6	0,0			5,44	15,2		-0,14	1,11	
			5		20,62223										
			6	4911	20,4477	20,5	0,0			5,22	15,2		-0,08	1,06	
			6		20,48119										
		Adlib	7	4470	20,6941	20,8	0,1	21,0	0,6	5,50	15,3	15,6	-0,07	1,05	0,85
			7		20,81939										
			8	4475	20,55329	20,7	0,1			5,03	15,6		0,30	0,81	
			8		20,75287										
			9	4476	22,15156	22,2	0,1			5,64	16,6		1,28	0,41	
			9		22,33962										

			10	4488	20,82933	20,8	0,0			5,34	15,5		0,18	0,88	
			10		20,85324										
		Fasted	11	4930	20,58254	20,6	0,0			5,36	15,2		-0,08	1,06	
			11		20,62581										
			12	4932	20,84543	20,9	0,0			5,33	15,5		0,21	0,87	
			12		20,87121										
			13	3935	21,02289	21,0	0,0	20,9	0,3	5,34	15,7	15,5	0,36	0,78	0,91
			13		21,01392										
			14	3938	21,04251	21,1	0,1			5,46	15,6		0,32	0,80	
			14		21,14571										
			15	4461	20,28748	20,4	0,2			5,20	15,2		-0,12	1,09	
		Fasted	15		20,50166										
			16	4462	20,83022	20,8	0,0			5,60	15,3		-0,07	1,05	
			16		20,86743										
			17	4464	20,9155	20,9	0,0			5,44	15,5		0,13	0,91	
			17		20,88014										
			18	B71	20,965	21,0	0,0			5,41	15,6		0,25	0,84	
Old	Chow		18		21,01428										
			19	3931	20,83955	20,9	0,1	20,8	0,2	5,47	15,4	15,5	0,10	0,93	0,89
			19		20,93169										
			20	3933	20,72004	20,7	0,0			5,30	15,4		0,09	0,94	
			20		20,71362										
			21	4459	20,71189	20,8	0,2			5,14	15,7		0,36	0,78	
		Adlib	21		20,92836										
			22	4460	20,69088	20,7	0,0			5,45	15,3		-0,07	1,05	
			22		20,70869										
			23	4466	20,57373	20,6	0,0			5,24	15,3		0,02	0,99	
			23		20,58634										
			24	B83	21,34747	21,3	0,1			5,34	15,9		0,61	0,65	
			24		21,19454										
			25	4366	20,499	20,6	0,1	20,7	0,3	5,55	15,0		-0,31	1,24	1,17
			25		20,61699										
		Fasted	26	4367	20,49148	20,5	0,0			5,61	14,9		-0,41	1,33	
			26		20,54603										
			27	4354	21,0963	21,1	0,0			5,68	15,4		0,10	0,93	
			27		21,12346										
			28	4302	20,70186	20,7	0,0	20,8	0,3	5,51	15,2		-0,14	1,10	0,98
Short	HFD		28		20,69214										
			29	4352	21,11184	21,2	0,1			5,42	15,8		0,43	0,74	
			29		21,22452										
		Adlib	30	4364	20,44309	20,5	0,1			5,50	15,0		-0,31	1,24	
			30		20,59551										
			31	4365	20,73296	20,8	0,1			5,19	15,6		0,26	0,84	
			31		20,81735										
			32	3929	21,16893	21,1	0,0	21,3	0,3	5,36	15,8	15,8	0,47	0,72	0,74
			32		21,13095										
			33	3934	21,09683	21,1	0,0			5,31	15,8		0,46	0,73	
			33		21,09307										
			34	4166	20,93989	21,0	0,1			5,51	15,5		0,20	0,87	
		[illegible]	34		21,1332										

		Fasted	35	4484	20,96358	21,0	0,1			5,62	15,4		0,10	0,93	
			35		21,11999										
			36	4485	21,72774	21,6	0,1			5,68	16,0		0,63	0,65	
			36		21,53842										
			37	4486	21,66753	21,7	0,0			5,56	16,1		0,81	0,57	
Long	HFD		37		21,73269										
			38	3939	21,00287	21,0	0,0	21,3	0,4	5,51	15,5	15,8	0,18	0,88	0,72
			38		21,02528										
			39	4167	21,21908	21,2	0,1			5,48	15,7		0,36	0,78	
			39		21,10399										
			40	4169	21,09272	21,1	0,0			5,22	15,9		0,53	0,69	
		Adlib	40		21,05176										
			41	4472	21,05337	21,1	0,0			5,42	15,6		0,31	0,81	
			41		21,05909										
			42	4413	21,51469	21,6	0,1			5,75	15,8		0,48	0,72	
			42		21,59138										
			43	4414	22,12498	22,1	0,0			5,60	16,5		1,20	0,43	
			43		22,13148										

2018-12-17

AoD	assay-#	Lot
Ldhb	Mm01267402_m1	

	1	2	3	4	5	6	7	8	9	10	11	12
A	1	1	9	9	17	17	25	25	33	33	41	41
B	2	2	10	10	18	18	26	26	34	34	42	42
C	3	3	11	11	19	19	27	27	35	35	43	43
D	4	4	12	12	20	20	28	28	36	36	nc	nc
E	5	5	13	13	21	21	29	29	37	37		
F	6	6	14	14	22	22	30	30	38	38		
G	7	7	15	15	23	23	31	31	39	39		
H	8	8	16	16	24	24	32	32	40	40		

Preparation of mastermix for duplicates

		MM
No samples	44,3	32
Sample copy	2	
universal mix [2x]	10	886
AoD	1	88,6
Nuclease free-H2O	8	708,8
50ng cDNA/well	1	
Total (ul)	20	1683,4

Relative to young chow fasted

Kidney

Ldhb										Ldhb					
	Diet	Nutstat	Sample	Mouse	Ct	Mean	SD mean	Group mea	SD group	18S	dCt	mean of dCt	DDct	2^DDct	mean of 2^DDct
			1	4409	15,57839	15,9	0,5	16,0	0,4	5,60	10,3	10,4573	-0,12	1,09	1,07
			1		16,28261										
		Fasted	2	4468	15,06709	15,3	0,4			5,61	9,7		-0,73	1,66	
			2		15,60335										
			3	4487	16,39079	16,5	0,1			5,42	11,0		0,59	0,66	
			3		16,55019										
			4	4478	15,68161	16,0	0,5			5,38	10,6		0,17	0,89	
			4		16,32192										
		Adlib	5	4910	15,63152	15,9	0,4			5,44	10,4		-0,01	1,01	
			5		16,15075										
			6	4911	16,24548	16,3	0,1			5,22	11,1		0,61	0,65	
			6		16,33342										
Young	Chow		7	4470	15,44622	15,2	0,4	15,8	0,6	5,50	9,7	10,5	-0,77	1,70	0,99
			7		14,93564										
		Adlib	8	4475	15,77412	15,8	0,1			5,03	10,8		0,33	0,80	
			8		15,86726										
			9	4476	16,5855	16,3	0,4			5,64	10,6		0,19	0,88	
			9		15,9846										
			10	4488	15,70719	16,0	0,5			5,34	10,7		0,24	0,85	

			10		16,3526										
		Fasted	11	4930	16,3101	16,5	0,3			5,36	11,1		0,67	0,63	
			11		16,66684										
			12	4932	14,98666	15,1	0,2			5,33	9,8		-0,64	1,55	
			12		15,31137										
			13	3935	15,98296	16,0	0,1	15,8	0,4	5,34	10,7	10,4	0,24	0,85	1,11
			13		16,0945										
			14	3938	16,90018	16,3	0,8			5,46	10,9		0,43	0,74	
			14		15,79132										
			15	4461	15,47478	15,5	0,0			5,20	10,3		-0,20	1,15	
		Fasted	15		15,43637										
			16	4462	14,95018	15,3	0,4			5,60	9,7		-0,79	1,73	
			16		15,57139										
			17	4464	15,3132	15,5	0,3			5,44	10,0		-0,41	1,33	
			17		15,6677										
			18	B71	15,87915	16,1	0,3			5,41	10,7		0,25	0,84	
Old	Chow		18		16,35904										
			19	3931	16,04697	16,1	0,0	16,1	0,3	5,47	10,6	10,8	0,14	0,91	0,81
			19		16,07129										
			20	3933	16,35089	16,1	0,3			5,30	10,8		0,39	0,76	
			20		15,94134										
			21	4459	16,31819	16,2	0,2			5,14	11,1		0,60	0,66	
		Adlib	21		16,06772										
			22	4460	16,37446	16,5	0,2			5,45	11,1		0,62	0,65	
			22		16,6697										
			23	4466	16,71876	16,3	0,6			5,24	11,1		0,60	0,66	
			23		15,88141										
			24	B83	15,55064	15,5	0,0			5,34	10,2		-0,26	1,20	
			24		15,51751										
			25	4366	15,79848	16,1	0,5	15,7	0,6	5,55	10,6	10,1	0,12	0,92	1,40
			25		16,44128										
		Fasted	26	4367	16,04929	15,9	0,1			5,61	10,3		-0,11	1,08	
			26		15,85029										
			27	4354	14,8962	15,0	0,2			5,68	9,3		-1,13	2,19	
			27		15,12771										
Short	HFD		28	4302	16,01428	16,1	0,1	16,0	0,3	5,51	10,6	10,6	0,10	0,94	0,89
			28		16,11373										
			29	4352	15,249	15,7	0,7			5,42	10,3		-0,16	1,11	
		Adlib	29		16,19114										
			30	4364	16,09314	16,4	0,5			5,50	10,9		0,47	0,72	
			30		16,76313										
			31	4365	16,32081	16,0	0,5			5,19	10,8		0,33	0,80	
			31		15,63184										
			32	3929	15,89758	16,0	0,1	16,1	0,4	5,36	10,6	10,6	0,14	0,91	0,94
			32		16,00922										
			33	3934	15,63033	15,9	0,3			5,31	10,6		0,09	0,94	
			33		16,09847										
			34	4166	16,05351	15,9	0,2			5,51	10,4		-0,05	1,04	
		Fasted	34		15,77584										
			35	4484	15,42305	15,6	0,2			5,62	10,0		-0,49	1,40	

Long	HFD		35		15,75313										
			36	4485	16,48082	16,7	0,3			5,68	11,0		0,52	0,70	
			36		16,8393										
			37	4486	16,60291	16,6	0,0			5,56	11,0		0,56	0,68	
			37		16,56152										
		Adlib	38	3939	16,03133	16,0	0,1	16,1	0,4	5,51	10,5	10,6	0,00	1,00	0,96
			38		15,91519										
			39	4167	16,48765	16,6	0,1			5,48	11,1		0,64	0,64	
			39		16,66357										
			40	4169	15,91888	15,6	0,5			5,22	10,4		-0,09	1,06	
			40		15,26142										
			41	4472	15,51192	15,8	0,4			5,42	10,4		-0,08	1,05	
			41		16,09435										
			42	4413	16,32543	15,9	0,7			5,75	10,1		-0,35	1,27	
			42		15,39649										
			43	4414	16,52777	16,5	0,0			5,60	10,9		0,48	0,72	
			43		16,55012										

Heart

cDNA-Number	samples: Mouse nr	Age	Diet	Nut.stat	conc. [µg/µl] RNA [ng/µl]	1900 ng motsvarar:	H_2O add 10 µl
1	4409	Young	Chow	Fasted	390,42	4,87	5,13
2	4468				540,721	3,51	6,49
3	4487				424,103	4,48	5,52
4	4488				394,911	4,81	5,19
5	4930				295,594	6,43	3,57
6	4932				323,046	5,88	4,12
7	4470	Young	Chow	Adlib	376,601	5,05	4,95
8	4475				472,651	4,02	5,98
9	4476				529,681	3,59	6,41
10	4478				409,163	4,64	5,36
11	4910				193	9,84	0,16
12	4911				272,966	6,96	3,04
13	3935	Old	Chow	Fasted	248,083	7,66	2,34
14	3938				260,961	7,28	2,72
15	4461				288,648	6,58	3,42
16	4462				406,609	4,67	5,33
17	4464				412,589	4,61	5,39
18	B71				503,872	3,77	6,23
19	3931	Old	Chow	Adlib	430,758	4,41	5,59
20	3933				624,146	3,04	6,96
21	4459				615,676	3,09	6,91
22	4460				476,564	3,99	6,01
23	4466				454,87	4,18	5,82
24	B83				439,941	4,32	5,68
25	4366	Short	HFD	Fasted	768,903	2,47	7,53
26	4367				549,992	3,45	6,55
27	4354				733,673	2,59	7,41
28	4302	Short	HFD	Adlib	755,019	2,52	7,48
29	4352				780,118	2,44	7,56
30	4364				659,761	2,88	7,12
31	4365				764,222	2,49	7,51
32	3929	Long	HFD	Fasted	466,858	4,07	5,93
33	3934				465,473	4,08	5,92
34	4166				796,668	2,38	7,62
35	4484				575,352	3,30	6,70
36	4485				581,317	3,27	6,73
37	4486				624,479	3,04	6,96
38	3939	Long	HFD	Adlib	339,776	5,59	4,41
39	4167				656,988	2,89	7,11
40	4169				914,077	2,08	7,92
41	4472				400,485	4,74	5,26
42	4413				389,041	4,88	5,12
43	4414				324,146	5,86	4,14

Date:2019-01-08

**Reverse transcription:**	**RT+**	
Number of samples	43	
Number of control RNA	0	
Margin of safety	1,04	
Total of tubes to prepare	44,72	
	volume / tube	**total volume to prepare**
5xRT buffer (5X)	4,00 µl	**178,88 µl**
dNTPs Mix (10mM)	2,00 µl	**89,44 µl**
Random primers (p(dN)6)	1,00 µl	**44,72 µl**
transcriptase (200U/µL)	1,00 µl	**44,72 µl**
Rnase Inhibitor [Rnasin], 40 U/µ	0,20 µl	**8,94 µl**
Nuclease-free (DEPC-treated) H	1,80 µl	**80,50 µl**
Sub-total	**10,00 µl**	**447,20 µl**
Sample RNAs	**10 µl**	
**Total**	**20 µl**	
**Programm.Nummer:**	10 min	25 °C
**Direction 5, Programm 1**	60 min	42 °C
	10 min	70 °C
		4 °C

2019-01-08

AoD	assay-#	Lot
18S	Mm03928990_g1	

	1	2	3	4	5	6	7	8	9	10	11	12
A	1	1	9	9	17	17	25	25	33	33	41	41
B	2	2	10	10	18	18	26	26	34	34	42	42
C	3	3	11	11	19	19	27	27	35	35	43	43
D	4	4	12	12	20	20	28	28	36	36	nc	nc
E	5	5	13	13	21	21	29	29	37	37		
F	6	6	14	14	22	22	30	30	38	38		
G	7	7	15	15	23	23	31	31	39	39		
H	8	8	16	16	24	24	32	32	40	40		

Preparation of mastermix for duplicates (18S)

		MM
No samples	44,1	
Sample copy	2	
universal mix [2x]	10	882
AoD	1	88,2
Nuclease free-H2O	8	705,6
50ng cDNA/well	1	
Total (ul)	20	1675,8

Heart

18S

	Diet	Nutstat	Mouse	Sample	Ct	Mean	SD mean	Group me	SD group	Group me	SD group
Young	Chow	Fasted	4409	1	7,587208	7,72	0,18	7,82	0,07	7,82	0,083637
				1	7,848033						
			4468	2	7,697373	7,75	0,08				
				2	7,809522						
			4487	3	7,800341	7,84	0,06				
				3	7,886456						
			4488	4	7,842236	7,89	0,07				
				4	7,936348						
			4930	5	7,856767	7,85	0,00				
				5	7,852789						
			4932	6	7,781911	7,83	0,07				
				6	7,881787						
		Adlib	4470	7	7,76662	7,74	0,03	7,82	0,11		
				7	7,721881						
			4475	8	7,86217	7,90	0,06				
				8	7,947788						
			4476	9	7,567562	7,64	0,10				
				9	7,704166						
			4478	10	7,82384	7,87	0,06				
				10	7,908776						
			4910	11	7,823604	7,86	0,06				
				11	7,901444						
			4911	12	7,895573	7,89	0,01				
				12	7,8816						
Old	Chow	Fasted	3935	13	8,423025	8,42	0,00	8,09	0,22	8,01	0,234346
				13	8,417401						
			3938	14	8,311993	8,28	0,04				
				14	8,251579						
			4461	15	8,020835	8,09	0,10				
				15	8,167278						
			4462	16	7,888019	7,92	0,05				
				16	7,9603						
			4464	17	7,960157	7,99	0,05				
				17	8,024102						
			B71	18	7,809274	7,83	0,03				
				18	7,84708						
		Adlib	3931	19	8,303211	8,33	0,03	7,93	0,23		
				19	8,349249						
			3933	20	8,044236	8,09	0,06				
				20	8,126367						
			4459	21	7,702295	7,80	0,14				
				21	7,893685						
			4460	22	7,740252	7,84	0,14				
				22	7,94391						
			4466	23	7,747119	7,74	0,01				
				23	7,730945						
			B83	24	7,70818	7,76	0,07				
				24	7,813957						
Short	HFD	Fasted	4366	25	7,55396	7,57	0,03	7,72	0,1	7,84	0,233907
				25	7,590641						
			4367	26	7,787832	7,81	0,03				
				26	7,827433						
			4354	27	7,763977	7,79	0,03				
				27	7,812878						
		Adlib	4302	28	7,749157	7,74	0,02	7,93	0,3		
				28	7,724519						
			4352	29	8,028291	8,03	0,01				
				29	8,038531						
			4364	30	7,670552	7,70	0,04				
				30	7,72466						
			4365	31	8,283464	8,27	0,02				
				31	8,256012						
Long	HFD	Fasted	3929	32	8,203238	8,31	0,15	7,91	0,20	7,83	0,185776
				32	8,409836						
			3934	33	7,843247	7,90	0,09				
				33	7,966599						
			4166	34	7,798188	7,82	0,03				
				34	7,841745						
			4484	35	7,840745	7,85	0,01				
				35	7,858839						
			4485	36	7,768039	7,77	0,01				
				36	7,779371						
			4486	37	7,752253	7,80	0,06				
				37	7,838688						
		Adlib	3939	38	7,974019	7,99	0,03	7,75	0,14		
				38	8,011399						
			4167	39	7,664246	7,63	0,04				
				39	7,605568						
			4169	40	7,628479	7,58	0,07				
				40	7,533154						
			4472	41	7,749927	7,74	0,01				
				41	7,732273						
			4413	42	7,748008	7,78	0,04				
				42	7,804924						
			4414	43	7,759934	7,76	0,00				
				43	7,762494						

2019-01-18

AoD	assay-#	Lot
Slc2a1	Mm00441480_m1	

	1	2	3	4	5	6	7	8	9	10	11	12
A	1	1	9	9	17	17	25	25	33	33	41	41
B	2	2	10	10	18	18	26	26	34	34	42	42
C	3	3	11	11	19	19	27	27	35	35	43	43
D	4	4	12	12	20	20	28	28	36	36	nc	nc
E	5	5	13	13	21	21	29	29	37	37		
F	6	6	14	14	22	22	30	30	38	38		
G	7	7	15	15	23	23	31	31	39	39		
H	8	8	16	16	24	24	32	32	40	40		

Preparation of mastermix for duplicates

		MM
No samples	44,1	
Sample copy	2	
universal mix [2x]	10	882
AoD	1	88,2
Nuclease free-H2C	8	705,6
50ng cDNA/well	1	
Total (ul)	20	1675,8

Relative to young (fasted)

Heart

Slc2a1

	Diet	Nutstat	Sample	Mouse	Ct	Mean	SD mean	Group mea	SD group	18S	dCt	mean of d	DDct	2^DDct	mean of 2^DDct
			1	4409	25,89636	25,9	0,0	25,8	0,2	7,72	18,2	17,9414	0,26	0,83	1,01
			1		25,94547										
			2	4468	25,92385	25,9	0,0			7,75	18,1		0,21	0,87	
			2		25,87996										
			3	4487	25,74031	25,8	0,1			7,84	18,0		0,03	0,98	
		Fasted	3		25,88422										
			4	4488	25,32336	25,5	0,3			7,89	17,6		-0,31	1,24	
			4		25,7198										
			5	4930	25,86163	25,9	0,1			7,85	18,0		0,10	0,93	
			5		25,93985										
			6	4932	25,42941	25,5	0,1			7,83	17,6		-0,29	1,22	
Young	Chow		6		25,53337										
			7	4470	26,33948	26,4	0,1	26,3	0,2	7,74	18,6		0,69	0,62	0,71
			7		26,41212										
			8	4475	26,05541	26,2	0,2			7,90	18,3		0,35	0,79	
			8		26,33476										
			9	4476	26,06268	26,1	0,1			7,64	18,5		0,53	0,69	
		Adlib	9		26,14334										
			10	4478	26,56961	26,5	0,1			7,87	18,7		0,72	0,61	
			10		26,48375										
			11	4910	26,4364	26,5	0,1			7,86	18,6		0,68	0,62	
			11		26,53224										
			12	4911	25,93127	26,0	0,0			7,89	18,1		0,12	0,92	
			12		25,97213										
			13	3935	26,92764	26,9	0,0	26,4	0,4	8,42	18,5	18,3	0,56	0,68	0,77
			13		26,92054										
			14	3938	26,5246	26,6	0,1			8,28	18,3		0,35	0,79	
			14		26,61504										
			15	4461	26,47996	26,5	0,1			8,09	18,4		0,48	0,72	
		Fasted	15		26,55179										
			16	4462	26,38982	26,4	0,0			7,92	18,5		0,55	0,69	
			16		26,4323										
			17	4464	26,40768	26,4	0,0			7,99	18,4		0,48	0,72	
			17		26,41965										
			18	B71	25,68631	25,7	0,1			7,83	17,9		-0,03	1,02	
Old	Chow		18		25,78882										
			19	3931	26,93437	26,9	0,0	26,4	0,3	8,33	18,6	18,5	0,65	0,64	0,70
			19		26,91055										
			20	3933	26,46605	26,5	0,1			8,09	18,4		0,49	0,71	
			20		26,56607										
			21	4459	26,10571	26,2	0,1			7,80	18,4		0,44	0,74	
		Adlib	21		26,24888										
			22	4460	26,06318	26,1	0,1			7,84	18,3		0,32	0,80	
			22		26,14309										
			23	4466	26,24892	26,3	0,1			7,74	18,6		0,62	0,65	
			23		26,34423										
			24	B83	26,20072	26,3	0,1			7,76	18,5		0,59	0,66	
			24		26,38347										
			25	4366	26,29009	26,3	0,0	26,3	0,2	7,57	18,7		0,80	0,57	0,66
			25		26,34657										
		Fasted	26	4367	26,3894	26,4	0,0			7,81	18,6		0,67	0,63	
			26		26,4459										
			27	4354	26,0346	26,1	0,1			7,79	18,3		0,35	0,78	
			27		26,12811										
Short	HFD		28	4302	26,52097	26,5	0,0	26,7	0,3	7,74	18,8		0,84	0,56	0,56
			28		26,52273										
			29	4352	26,53096	26,6	0,0			8,03	18,5		0,59	0,67	
		Adlib	29		26,59156										
			30	4364	26,61037	26,6	0,0			7,70	18,9		0,94	0,52	
			30		26,5478										
			31	4365	27,251	27,2	0,0			8,27	19,0		1,02	0,49	
			31		27,20681										
			32	3929	26,9489	27,0	0,1	26,5	0,3	8,31	18,7	18,6	0,79	0,58	0,66
			32		27,11914										
			33	3934	26,33641	26,5	0,2			7,90	18,5		0,61	0,66	
			33		26,57132										
			34	4166	26,32269	26,3	0,0			7,82	18,5		0,59	0,67	
		Fasted	34		26,37586										
			35	4484	26,61056	26,6	0,0			7,85	18,8		0,82	0,57	
			35		26,61073										
			36	4485	26,20404	26,2	0,1			7,77	18,5		0,53	0,69	
			36		26,28806										
			37	4486	26,05031	26,1	0,0			7,80	18,3		0,33	0,80	
Long	HFD		37		26,0792										
			38	3939	26,74511	26,8	0,0	26,6	0,3	7,99	18,8	18,9	0,84	0,56	0,53
			38		26,81121										
			39	4167	26,53171	26,6	0,1			7,63	18,9		1,00	0,50	
			39		26,62051										
			40	4169	26,68587	26,7	0,0			7,58	19,1		1,18	0,44	
		Adlib	40		26,71853										
			41	4472	26,67732	26,8	0,2			7,74	19,0		1,10	0,47	
			41		26,88991										
			42	4413	26,90417	26,9	0,0			7,78	19,2		1,21	0,43	
			42		26,95625										
			43	4414	25,95202	26,1	0,2			7,76	18,3		0,37	0,77	
			43		26,19939										

2019-01-18

AoD	assay-#	Lot
Slc2a4		

	1	2	3	4	5	6	7	8	9	10	11	12
A	1	1	9	9	17	17	25	25	33	33	41	41
B	2	2	10	10	18	18	26	26	34	34	42	42
C	3	3	11	11	19	19	27	27	35	35	43	43
D	4	4	12	12	20	20	28	28	36	36	nc	nc
E	5	5	13	13	21	21	29	29	37	37		
F	6	6	14	14	22	22	30	30	38	38		
G	7	7	15	15	23	23	31	31	39	39		
H	8	8	16	16	24	24	32	32	40	40		

Preparation of mastermix for duplicates

		MM
No samples	44,1	
Sample copy	2	
universal mix [2x	10	882
AoD	1	88,2
Nuclease free-H2(	8	705,6
50ng cDNA/well	1	
Total (ul)	20	1675,8

Relative to young (fasted)

Heart

GLUT4

	Diet	Nutstat	Sample	Mouse	Ct	Mean	SD mean	Group mea	SD group	18S	dCt	mean of d	DDct	2^DDct	mean of 2^DDct
Young	Chow	Fasted	1	4409	21,94634	22,0	0,1	21,8	0,1	7,72	14,3	14,0149	0,26	0,83	1,00
			1		22,03913										
			2	4468	21,79992	21,8	0,0			7,75	14,1		0,07	0,96	
			2		21,86706										
			3	4487	21,68597	21,8	0,1			7,84	13,9		-0,10	1,07	
			3		21,83231										
			4	4488	21,68095	21,7	0,1			7,89	13,8		-0,18	1,13	
			4		21,77466										
			5	4930	21,76988	21,8	0,1			7,85	14,0		-0,03	1,02	
			5		21,91406										
			6	4932	21,7876	21,8	0,1			7,83	14,0		-0,02	1,02	
			6		21,86213										
		Adlib	7	4470	21,87056	21,9	0,0	21,9	0,1	7,74	14,1		0,12	0,92	0,95
			7		21,88395										
			8	4475	21,72764	21,8	0,1			7,90	13,9		-0,12	1,08	
			8		21,88139										
			9	4476	21,95114	22,0	0,0			7,64	14,3		0,31	0,81	
			9		21,96729										
			10	4478	21,92439	21,9	0,0			7,87	14,1		0,06	0,96	
			10		21,94841										
			11	4910	21,94455	21,9	0,0			7,86	14,1		0,07	0,95	
			11		21,94529										
			12	4911	21,93762	22,0	0,0			7,89	14,1		0,05	0,96	
			12		21,97254										
Old	Chow	Fasted	13	3935	22,52197	22,6	0,1	22,2	0,3	8,42	14,1	14,1	0,12	0,92	0,96
			13		22,59318										
			14	3938	22,28728	22,3	0,1			8,28	14,0		0,03	0,98	
			14		22,36767										
			15	4461	21,78461	21,8	0,1			8,09	13,7		-0,27	1,20	
			15		21,89702										
			16	4462	22,23997	22,3	0,0			7,92	14,3		0,33	0,80	
			16		22,29373										
			17	4464	22,38085	22,4	0,0			7,99	14,4		0,35	0,78	
			17		22,34204										
			18	B71	21,76364	21,8	0,0			7,83	13,9		-0,07	1,05	
			18		21,77931										
		Adlib	19	3931	22,3841	22,4	0,0	22,1	0,2	8,33	14,1	14,2	0,04	0,97	0,91
			19		22,37781										
			20	3933	22,12579	22,2	0,0			8,09	14,1		0,06	0,96	
			20		22,18749										
			21	4459	21,82322	21,9	0,1			7,80	14,1		0,08	0,95	
			21		21,95947										
			22	4460	21,99371	22,0	0,1			7,84	14,2		0,18	0,88	
			22		22,07674										
			23	4466	21,99659	22,0	0,0			7,74	14,2		0,23	0,85	
			23		21,97954										
			24	B83	21,97919	22,0	0,1			7,76	14,3		0,25	0,84	
			24		22,08164										
Short	HFD	Fasted	25	4366	22,16259	22,2	0,1	22,1	0,1	7,57	14,6		0,63	0,65	0,77
			25		22,27577										
			26	4367	22,06873	22,1	0,0			7,81	14,3		0,28	0,82	
			26		22,1346										
			27	4354	22,05581	22,1	0,0			7,79	14,3		0,27	0,83	
			27		22,09239										
		Adlib	28	4302	22,10056	22,1	0,1	22,3	0,3	7,74	14,4		0,39	0,76	0,76
			28		22,17927										
			29	4352	22,35719	22,4	0,0			8,03	14,4		0,34	0,79	
			29		22,41171										
			30	4364	22,10895	22,2	0,1			7,70	14,5		0,44	0,74	
			30		22,19306										
			31	4365	22,69674	22,7	0,0			8,27	14,5		0,44	0,74	
			31		22,75145										
Long	HFD	Fasted	32	3929	22,99943	23,0	0,0	22,4	0,3	8,31	14,7	14,5	0,70	0,61	0,72
			32		23,04744										
			33	3934	22,29978	22,3	0,0			7,90	14,4		0,39	0,76	
			33		22,32226										
			34	4166	22,16342	22,2	0,1			7,82	14,4		0,37	0,77	
			34		22,24478										
			35	4484	22,34087	22,4	0,0			7,85	14,5		0,49	0,71	
			35		22,37359										
			36	4485	22,24602	22,3	0,1			7,77	14,5		0,52	0,70	
			36		22,36876										
			37	4486	22,21953	22,2	0,0			7,80	14,4		0,43	0,74	
			37		22,26916										
		Adlib	38	3939	22,30786	22,3	0,0	22,3	0,1	7,99	14,3	14,5	0,33	0,79	0,71
			38		22,37645										
			39	4167	22,2252	22,3	0,1			7,63	14,6		0,63	0,65	
			39		22,32484										
			40	4169	21,99571	22,0	0,0			7,58	14,4		0,43	0,74	
			40		22,06087										
			41	4472	22,29618	22,4	0,1			7,74	14,6		0,61	0,66	
			41		22,43573										
			42	4413	22,31975	22,4	0,1			7,78	14,6		0,59	0,66	
			42		22,44742										
			43	4414	22,15943	22,2	0,0			7,76	14,4		0,40	0,76	
			43		22,18576										

2019-01-18

AoD	assay-#	Lot
Slc27a6		

	1	2	3	4	5	6	7	8	9	10	11	12
A	1	1	9	9	17	17	25	25	33	33	41	41
B	2	2	10	10	18	18	26	26	34	34	42	42
C	3	3	11	11	19	19	27	27	35	35	43	43
D	4	4	12	12	20	20	28	28	36	36	nc	nc
E	5	5	13	13	21	21	29	29	37	37		
F	6	6	14	14	22	22	30	30	38	38		
G	7	7	15	15	23	23	31	31	39	39		
H	8	8	16	16	24	24	32	32	40	40		

Preparation of mastermix for duplicates

		MM
No samples	44,1	
Sample copy	2	
universal mix [2x	10	882
AoD	1	88,2
Nuclease free-H2(	8	705,6
50ng cDNA/well	1	
Total (ul)	20	1675,8

Relative to young (fasted)

Heart

Cd36

	Diet	Nutstat	Sample	Mouse	Ct	Mean	SD mean	Group mea	SD group	18S	dCt	mean of d(	DDct	2^DDct	mean of 2^DDct
			1	4409	33,4456	33,4	0,1	33,6	0,3	7,72	25,7	25,7433	-0,08	1,05	1,02
			1		33,32287										
			2	4468	33,75488	33,7	0,1			7,75	26,0		0,21	0,86	
			2		33,65836										
			3	4487	33,89459	33,7	0,3			7,84	25,8		0,10	0,93	
		Fasted	3		33,48815										
			4	4488	32,97346	33,1	0,2			7,89	25,2		-0,54	1,46	
			4		33,20457										
			5	4930	33,66155	33,7	0,1			7,85	25,9		0,12	0,92	
			5		33,7715										
			6	4932	33,6564	33,8	0,1			7,83	25,9		0,19	0,88	
Young	Chow		6		33,86824										
			7	4470	32,88265	33,0	0,2	33,1	0,4	7,74	25,3		-0,46	1,38	1,37
			7		33,17175										
			8	4475	32,97207	33,0	0,1			7,90	25,1		-0,60	1,52	
			8		33,12216										
			9	4476	32,96312	33,1	0,2			7,64	25,4		-0,30	1,23	
		Adlib	9		33,18836										
			10	4478	32,95948	33,2	0,3			7,87	25,3		-0,42	1,33	
			10		33,42954										
			11	4910	33,84509	33,8	0,0			7,86	26,0		0,24	0,85	
			11		33,83722										
			12	4911	32,59851	32,7	0,1			7,89	24,8		-0,94	1,92	
			12		32,78848										
			13	3935	33,91978	34,3	0,5	34,0	0,3	8,42	25,9	25,9	0,13	0,92	0,88
			13		34,6581										
			14	3938	34,12046	34,4	0,4			8,28	26,1		0,38	0,77	
			14		34,68916										
			15	4461	33,49334	33,9	0,5			8,09	25,8		0,02	0,98	
		Fasted	15		34,22879										
			16	4462	34,05432	34,2	0,2			7,92	26,3		0,52	0,70	
			16		34,32015										
			17	4464	33,70944	33,7	0,0			7,99	25,7		0,00	1,00	
			17		33,75172										
			18	B71	33,65583	33,7	0,0			7,83	25,9		0,12	0,92	
Old	Chow		18		33,71869										
			19	3931	34,11886	34,1	0,0	33,8	0,4	8,33	25,8	25,9	0,06	0,96	0,92
			19		34,14035										
			20	3933	33,42868	33,4	0,1			8,09	25,3		-0,45	1,37	
			20		33,32194										
			21	4459	34,64064	34,4	0,4			7,80	26,6		0,84	0,56	
		Adlib	21		34,11241										
			22	4460	33,76759	33,8	0,0			7,84	26,0		0,21	0,86	
			22		33,8247										
			23	4466	33,85752	33,8	0,1			7,74	26,0		0,30	0,81	
			23		33,70917										
			24	B83	33,51251	33,6	0,1			7,76	25,8		0,06	0,96	
			24		33,60686										
			25	4366	33,37168	33,4	0,0	33,0	0,4	7,57	25,8		0,07	0,95	1,48
			25		33,40917										
		Fasted	26	4367	32,83604	32,8	0,0			7,81	25,0		-0,70	1,63	
			26		32,86025										
			27	4354	32,39793	32,6	0,3			7,79	24,8		-0,90	1,87	
			27		32,85798										
Short	HFD		28	4302	32,92845	32,9	0,0	32,9	0,4	7,74	25,2		-0,54	1,45	1,77
			28		32,95491										
			29	4352	32,43379	32,5	0,1			8,03	24,4		-1,29	2,45	
		Adlib	29		32,53001										
			30	4364	32,84714	32,7	0,1			7,70	25,0		-0,70	1,62	
			30		32,63811										
			31	4365	33,29829	33,4	0,1			8,27	25,1		-0,62	1,53	
			31		33,49208										
			32	3929	35,03032	34,7	0,4	33,4	0,7	8,31	26,4	25,5	0,69	0,62	1,22
			32		34,45221										
			33	3934	33,55809	33,7	0,2			7,90	25,8		0,03	0,98	
			33		33,79877										
			34	4166	32,90133	33,0	0,2			7,82	25,2		-0,53	1,45	
		Fasted	34		33,16105										
			35	4484	33,64367	33,5	0,2			7,85	25,6		-0,11	1,08	
			35		33,32573										
			36	4485	32,81444	32,8	0,0			7,77	25,1		-0,69	1,61	
			36		32,84473										
			37	4486	32,85312	32,9	0,0			7,80	25,1		-0,65	1,57	
Long	HFD		37		32,92212										
			38	3939	33,55787	33,7	0,3	33,4	0,4	7,99	25,8	25,6	0,01	0,99	1,11
			38		33,93503										
			39	4167	33,13818	33,2	0,0			7,63	25,5		-0,23	1,17	
			39		33,16751										
			40	4169	33,19378	33,1	0,1			7,58	25,5		-0,20	1,15	
		Adlib	40		33,04604										
			41	4472	33,74544	33,8	0,0			7,74	26,0		0,29	0,82	
			41		33,79413										
			42	4413	33,71889	33,7	0,0			7,78	25,9		0,17	0,89	
			42		33,67051										
			43	4414	32,8877	32,8	0,1			7,76	25,0		-0,71	1,64	
			43		32,70041										

2019-01-08

AoD	assay-#	Lot
Cd36	Mm00432403_m1	

	1	2	3	4	5	6	7	8	9	10	11	12
A	1	1	9	9	17	17	25	25	33	33	41	41
B	2	2	10	10	18	18	26	26	34	34	42	42
C	3	3	11	11	19	19	27	27	35	35	43	43
D	4	4	12	12	20	20	28	28	36	36	nc	nc
E	5	5	13	13	21	21	29	29	37	37		
F	6	6	14	14	22	22	30	30	38	38		
G	7	7	15	15	23	23	31	31	39	39		
H	8	8	16	16	24	24	32	32	40	40		

Preparation of mastermix for duplicates (Cd36)

		MM
No samples	44,1	
Sample cop	2	
universal mix [2x	10	882
AoD	1	88,2
Nuclease free-H2	8	705,6
50ng cDNA/well	1	
Total (ul)	20	1675,8

Relative to young (fasted)

Heart

Cd36

	Diet	Nutstat	Sample	Mouse	Ct	Mean	SD mean	Group me	SD group	18S	dCt	mean of dCt	DDct	2^DDct	mean of 2^DDct
Young	Chow	Fasted	1	4409	18,26715	18,4	0,1	18,4	0,1	7,72	10,7	10,5848	0,07	0,96	1,00
			1		18,46852										
			2	4468	18,33382	18,4	0,0			7,75	10,6		0,03	0,98	
			2		18,3987										
			3	4487	18,46168	18,5	0,0			7,84	10,7		0,07	0,95	
			3		18,53223										
			4	4488	18,35267	18,4	0,0			7,89	10,5		-0,10	1,07	
			4		18,39668										
			5	4930	18,43355	18,5	0,1			7,85	10,6		0,06	0,96	
			5		18,5715										
			6	4932	18,38035	18,3	0,1			7,83	10,5		-0,13	1,09	
			6		18,20202										
		Adlib	7	4470	18,77064	18,7	0,0	18,8	0,1	7,74	11,0		0,41	0,75	0,77
			7		18,71551										
			8	4475	18,73744	18,8	0,1			7,90	10,9		0,29	0,82	
			8		18,82931										
			9	4476	18,5571	18,6	0,1			7,64	11,0		0,37	0,77	
			9		18,63163										
			10	4478	18,76606	18,8	0,0			7,87	10,9		0,34	0,79	
			10		18,81622										
			11	4910	18,73991	18,8	0,1			7,86	11,0		0,39	0,76	
			11		18,93612										
			12	4911	18,93278	18,9	0,0			7,89	11,0		0,43	0,74	
			12		18,87058										
Old	Chow	Fasted	13	3935	19,17992	19,1	0,1	18,6	0,3	8,42	10,7	10,5	0,08	0,94	1,03
			13		18,99864										
			14	3938	18,79484	18,7	0,1			8,28	10,4		-0,14	1,11	
			14		18,6494										
			15	4461	18,55911	18,6	0,1			8,09	10,5		-0,04	1,03	
			15		18,72364										
			16	4462	18,54993	18,6	0,0			7,92	10,7		0,07	0,95	
			16		18,60295										
			17	4464	18,40704	18,4	0,0			7,99	10,4		-0,16	1,11	
			17		18,43453										
			18	B71	18,36494	18,4	0,0			7,83	10,6		-0,03	1,02	
			18		18,40286										
		Adlib	19	3931	18,94296	19,0	0,1	18,6	0,3	8,33	10,7	10,7	0,10	0,94	0,95
			19		19,07157										
			20	3933	18,78503	18,9	0,1			8,09	10,8		0,20	0,87	
			20		18,95229										
			21	4459	18,43375	18,6	0,2			7,80	10,8		0,21	0,87	
			21		18,74534										
			22	4460	18,38154	18,4	0,0			7,84	10,6		-0,01	1,01	
			22		18,4486										
			23	4466	18,38724	18,3	0,1			7,74	10,6		0,00	1,00	
			23		18,2509										
			24	B83	18,21907	18,3	0,1			7,76	10,5		-0,06	1,04	
			24		18,34815										
Short	HFD	Fasted	25	4366	17,95991	18,0	0,1	18,1	0,2	7,57	10,4		-0,16	1,11	1,15
			25		18,0439										
			26	4367	17,87011	18,0	0,1			7,81	10,2		-0,43	1,35	
			26		18,05465										
			27	4354	18,32032	18,4	0,1			7,79	10,6		0,03	0,98	
			27		18,48357										
		Adlib	28	4302	18,16483	18,2	0,0	18,5	0,4	7,74	10,4		-0,15	1,11	1,00
			28		18,18582										
			29	4352	18,81613	18,9	0,1			8,03	10,8		0,24	0,85	
			29		18,89491										
			30	4364	18,17934	18,2	0,0			7,70	10,5		-0,07	1,05	
			30		18,24331										
			31	4365	18,82605	18,8	0,0			8,27	10,6		-0,01	1,01	
			31		18,85953										
Long	HFD	Fasted	32	3929	18,94919	19,0	0,0	18,4	0,3	8,31	10,7	10,5	0,08	0,95	1,07
			32		18,99203										
			33	3934	18,27822	18,3	0,1			7,90	10,4		-0,15	1,11	
			33		18,41113										
			34	4166	18,20287	18,3	0,1			7,82	10,5		-0,12	1,09	
			34		18,3636										
			35	4484	18,2172	18,3	0,1			7,85	10,5		-0,11	1,08	
			35		18,42397										
			36	4485	18,0762	18,1	0,0			7,77	10,3		-0,26	1,19	
			36		18,1301										
			37	4486	18,28419	18,3	0,1			7,80	10,5		-0,04	1,03	
			37		18,40084										
		Adlib	38	3939	18,45943	18,5	0,0	18,2	0,2	7,99	10,5	10,5	-0,10	1,07	1,07
			38		18,4879										
			39	4167	18,35194	18,4	0,0			7,63	10,7		0,15	0,90	
			39		18,38496										
			40	4169	18,10276	18,2	0,2			7,58	10,6		0,06	0,96	
			40		18,34865										
			41	4472	17,97857	18,0	0,1			7,74	10,3		-0,31	1,24	
			41		18,05436										
			42	4413	18,15651	18,2	0,0			7,78	10,4		-0,19	1,14	
			42		18,19499										
			43	4414	18,09288	18,2	0,2			7,76	10,5		-0,13	1,10	
			43		18,33168										
